# Scale evolution in Paraphysomonadida (Chrysophyceae): Sequence phylogeny and revised taxonomy of *Paraphysomonas*, new genus *Clathromonas*, and 25 new species

**DOI:** 10.1016/j.ejop.2014.08.001

**Published:** 2014-10

**Authors:** Josephine Margaret Scoble, Thomas Cavalier-Smith

**Affiliations:** Department of Zoology, University of Oxford, South Parks Road, Oxford OX1 3PS, UK

**Keywords:** *Clathromonas*, Chrysophyte, 18S rDNA phylogeny, Heterokont, *Paraphysomonas vestita*, Scale ultrastructure

## Abstract

Heterotrophic chrysomonads of the genus *Paraphysomonas* are ubiquitous phagotrophs with diverse silica scale morphology. Over 50 named species have been described by electron microscopy from uncultured environmental samples. Sequence data exist for very few, but the literature reveals misidentification or lumping of most previously sequenced. For critically integrating scale and sequence data, 59 clonal cultures were studied light microscopically, by sequencing 18S ribosomal DNA, and recording scale morphology by transmission electron microscopy. We found strong congruence between variations in scale morphology and rDNA sequences, and unexpectedly deep genetic diversity. We now restrict *Paraphysomonas* to species with nail-like spine scales, establishing 23 new species and eight subspecies (Paraphysomonadidae). Species having base-plates with dense margins form three distinct subclades; those with a simple margin only two. We move 29 former *Paraphysomonas* species with basket scales into a new genus, *Clathromonas*, and describe two new species. *Clathromonas* belongs to a very distinct rDNA clade (Clathromonadidae fam. n.), possibly distantly sister to *Paraphysomonas*. Molecular and morphological data are mutually reinforcing; both are needed for evaluating paraphysomonad diversity and confirm excessive past lumping. Former *Paraphysomonas* species with neither nail-like nor basket scales are here excluded from *Paraphysomonas* and will be assigned to new genera elsewhere.

## Introduction

Colourless chrysomonads of the genera *Paraphysomonas* and *Spumella* are major phagotrophs in freshwater and soil food webs, and *Paraphysomonas* is also widespread in marine environments (Charvet et al., 2011, del Campo and Massana, 2011, Massana et al., 2004, Massana et al., 2006, Massana et al., 2014, Richards and Bass, 2005). These important feeders on bacteria have received considerable experimental study (Jürgens et al., 1997, Lim et al., 1999, Pfandl et al., 2004, Simek et al., 1997, Zwirglmaier et al., 2009), but their taxonomy is unsatisfactory and needs major revision. Ribosomal DNA phylogeny showed that *Spumella* is certainly polyphyletic; about five non-scaly chrysophyte lineages independently lost photosynthesis and thus became *Spumella*-like in morphology (Boenigk et al., 2005, Boenigk, 2008); eventually they must be divided into several genera. *Paraphysomonas* differs from *Spumella* by having numerous silica scales on its cell body, but it is easy to confuse them by light microscopy, which does not reveal the scales of most species; so many strains and their sequences have merely been called ‘*Spumella*-like’ (Boenigk et al. 2005). Traditionally, *Paraphysomonas* was grouped with three photosynthetic genera *Chrysosphaerella*, *Spiniferomonas*, and *Polylepidomonas* in family Paraphysomonadaceae (Preisig 1995); more recently these were excluded, *Paraphysomonas* alone constituting a distinct chrysomonad order Paraphysomonadales (Cavalier-Smith and Chao 2006), which sequence trees often place as sister to all other chrysophytes (Škaloud et al. 2013). We focus here on the biodiversity and taxonomy of *Paraphysomonas* and show that several genera are needed to encompass their diversity, and more species than hitherto realised can be distinguished from *Spumella* in the light microscope.

Ultrastructural differences in scale morphology currently distinguish 56 – 57 *Paraphysomonas* species (Lucas, 1967, Lucas, 1968). The type species, *P. vestita*, is the only one not originally thus defined, having been discovered before electron microscopy (Stokes 1885 as *Physomonas vestita*). De Saedeleer (1929) changed its name to *Paraphysomonas vestita* because the type species (*Physomonas socialis*) was removed to another genus, *Monas*, now abandoned as a nomen dubium roughly corresponding with *Spumella* (see Silva 1960); however *Spumella* is itself polyphyletic and requires major revision (Boenigk 2008). *Paraphysomonas vestita* spine scales were first drawn by Korshikov (1929) as ‘nails with relatively large flat heads’. Houwink (1952) published the first electron micrographs of *Paraphysomonas* ‘*vestita’* spine scales, showing their circular base-plate and long central pointed spine. Subsequent ultrastructural studies and environmental surveys of silica-scaled protists have shown a great variety of broadly similar, yet distinctly different, nail-like scales under the umbrella name *P. vestita* (Manton and Leedale, 1961, Takahashi, 1976, Cronberg and Kristiansen, 1980, Thomsen et al., 1981, Santos and Leedale, 1993, Bergesch et al., 2008, Petronio and Rivera, 2010). It is unclear which, if any, of these structurally quite diverse scales are actually from *P vestita* or from undescribed species (see Scoble and Cavalier-Smith 2013). Hardly any *Paraphysomonas* species were described from clonal cultures, nearly all being named from a few cells collected directly from the environment and dried on electron microscope grids. There is therefore almost no knowledge of the range of variation of scales within a strain, still less a single species, causing identification problems.

Ribosomal DNA sequences are available for only five named *Paraphysomonas* species (Scoble and Cavalier-Smith 2013). Unfortunately, some sequences labelled as the same species (*P. vestita* and *P. foraminifera*) are so far apart on the trees and radically different that some sequenced strains must have been seriously misidentified; moreover one *P.* ‘*foraminifera*’ sequence (AB022864) is almost the same as one *P.* ‘*vestita*’ sequence (Z28335: Rice et al. 1997), differing in one inserted T. No ultrastructure was provided for most strains so their true identity is unknown and cultures no longer available for study. Some *Paraphysomonas* sequences were fortunately published together with electron micrographs of scales (Caron et al., 1999, Rice et al., 1997); in all cases their detailed structure differs from that of the type strains, suggesting that none was correctly identified. These mistakes and the rarity of combined sequence and morphological data are totally confusing for *Paraphysomonas* scale evolution. From environmental sequencing more different sequences are already known in the *Paraphysomonas* spine-scale clade than the total number of named spine-scaled species, so the assertion that most *Paraphysomonas* species are already known (Finlay and Clarke 1999a) was overconfident.

It has been claimed that *P. vestita* is the commonest and most widespread *Paraphysomonas* (Finlay and Clarke 1999b), but that could be an artefact of an excessively loose species definition (see Scoble and Cavalier-Smith 2013). The identity of the type species *P. vestita* is loosely defined: the original description tells us scarcely more than it was ∼15 μm with projecting spines, but strains under that name range from 8 to 26 μm and exhibit such a large range in scale morphology that they probably represent numerous species. Loose definition may also apply to some extent to the ‘second commonest’ species *P. imperforata* (Finlay and Clarke 1999b), whose relatively non-descript spine scales differ obviously from those attributed to *P. vestita* only by lacking a dense base-plate margin and from *P. foraminifera* merely by lacking holes on the base-plate, i.e. *P. imperforata* is negatively defined. The literature has not been critically reviewed until recently, but there are clearly subtle and some more obvious differences in broadly similar scale types for both ‘*P. vestita*’*-*like and *P. imperforata-*like scales, as noted by Scoble and Cavalier-Smith (2013).

To clarify these problems, and put *Paraphysomonas* taxonomy on a sounder footing, we studied 59 clonal cultures (mostly newly isolated) by light and electron microscopy and 18S rDNA sequencing; we describe 23 new species with spine scales (four based on previously published work), and show how differences in scale morphology map onto the 18S rDNA tree. In addition to eight previously known *Paraphysomonas* species with spine scales (i.e. *P. vestita, P. imperforata, P. foraminifera, P. bandaiensis, P. antarctica, P. circumforaminifera, P. porosa, P. oligocycla*), we include *P. cylicophora*, whose scales we regard as modified spine scales, and raise a former subspecies (*P. vestita truncata*) to species status. Thus spine-scale species now total 32 and constitute *Paraphysomonas* sensu stricto, which we make a much more homogeneous genus by excluding all species with other scale types.

Lucas (1968), in describing the first *Paraphysomonas* with latticed not spine scales, thought it might merit a separate genus, but unfortunately did not erect one. Others later suggested that the large array of ‘*Paraphysomonas*’ species with ever more diverse open-mesh scales may deserve generic separation (Leadbeater, 1972, Pennick and Clarke, 1973, Takahashi, 1976), but all conservatively left them in *Paraphysomonas* making it excessively heterogeneous. Unlike *Paraphysomonas* sensu stricto, species with latticed scales have two different scale types forming two layers: flat plate scales with perforations close to the plasma membrane and tiered crown scales outside them. We establish a new genus *Clathromonas* for 31 such species; they are part of an environmental DNA clade very distinct from the huge spine-scale clade (*Paraphysomonas* sensu stricto), though sometimes weakly group with it; we therefore keep both in Paraphysomonadida (=Paraphysomonadales; we use ICZN not IBN for this purely phagotrophic order of non-algae). We exclude all the numerous ‘*Paraphysomonas’* species having yet other, very different, scale types (most without spines, some with an open lattice as in *P. butcheri* (Pennick and Clarke, 1972)) from both *Paraphysomonas* and *Clathromonas*, placing them in new genera in another paper.

As many clades of chrysomonad DNA sequences of unknown phenotype were recently discovered (Charvet et al., 2011, del Campo and Massana, 2011), our trees include numerous representatives of them all to clarify their relationships to paraphysomonads and other chrysomonads, and to test the monophyly of Paraphysomonadida. We include representatives of all major chrysophyte clades and significant ochrophyte outgroups to provide a more comprehensive, more reliably rooted, chrysophyte tree than hitherto. We found seven deeply branching clades of Chrysophyceae containing known organisms, plus either one or two huge environmental clades of unknown phenotype, though 18S rDNA trees do not robustly establish relationships amongst these 8 – 9 major clades.

## Material and Methods

### Obtaining *Paraphysomonas* isolates

Clonal cultures of *Paraphysomonas* were obtained from soil, freshwater, and marine environments. Ten to 20 g of soil, sand or sediment and water were collected and a few grammes put into Petri dishes along with media (Artificial Salt Water for Protists (ASWP CCAP media recipes http://www.ccap.ac.uk/media/) or Volvic^®^ for freshwater samples) and were enriched with barley grain juice (tablespoon of barley grain in 100 ml Volvic^®^ bring to boil and filter water through 0.22 μm filter – put a few drops in the culture to encourage general growth of protists via bacterial food bloom) and left at ambient temperature for 48 h. These enriched cultures were examined by phase microscopy for the presence of *Paraphysomonas*-like cells; if present, 10 μl of the culture was serially diluted up to eight times in 96-well Plates – 12 copies of each dilution. Fourty eight hours later the 96 wells were checked for *Paraphysomonas*-like cells, further serial dilutions were performed at least another four times (every two days), and once a well was thought to contain a pure colony it was serially diluted twice more to give more chance of a pure clone being selected. Cell selection was initially based on size and basic features: large (∼≥7 μm) completely round cells with two visible cilia (one long one short), colourless and with a stalked stage. After these preliminary efforts yielded 100% spine-scaled *Paraphysomonas*, smaller cells were then targeted, adhering to the same other criteria as before, which is when *P. lucasi*, *P.* aff. *imperforata* and *Clathromonas butcheri* were found. Only round cells were chosen, often mainly those stalked to the substratum. Eight strains (JBM01, JBM02, WA20KP, WI34KN, WA28KT, PR26KB, PR26KA and AU30KV) were kindly provided by Jens Boenigk.

### DNA extraction

As soon as the new clonal culture was established one 9 cm Petri dish of the culture was extracted using UltraClean^®^ Soil DNA Isolation Kit. Whatman GF/F glass fibre (0.2 μm) filters were used to filter the cells and the filter chopped up and put into the soil extraction bead tube of the kit.

### PCR and sequencing

The same eukaryote-wide primers, targeting the 18S rDNA gene, were used in PCR and sequencing: 25F (forward: 5′-CATATGCTTGTCTCAAAGATTAAGCCA-3′), 1801R (reverse: 5′-TGATCCTTCTGCAGGTTCACCT-3′); these plus a third internal primer were used for sequencing: 3NDF (forward: 5′-GGCAAGTCTGGTGCCAG-3′). PCR reactions were mixed in 25 μl (using Invitrogen™ reagents). Denaturation (5 min at 95 °C) was followed by 35 cycles: 95 °C for 32 s; 60 °C for 30 s; 72 °C for 2 min. Final extension was for 7 min at 72 °C. Five microlitres of the PCR product was subjected to 1% agarose gel electrophoresis, and after ethidium bromide staining viewed under UV. If there were multiple bands the correct size PCR fragment (∼1800 kbp) was cut out and cleaned using a GE Healthcare GFX™ extraction kit. If there was a single band at the correct size, the PCR reaction was cleaned using polyethylene glycol (PEG): 25 μl PEG and 1 μl of 3 μM NaCl is added to each 25 μl reaction and mixed by vortex, kept 30 min at ambient temperature, and pelleted by centrifugation at 1500 RCF for 30 min. Supernatant was discarded, the pellet washed with 25 μl 70% EtOH, centrifuged again for 10 min before removing supernatant. Pellets were left to dry before resuspending in deionised water and storage at −20 °C. Sequencing used dye terminators and an automated ABI-377 sequencer. Editing was via free program Sequence Scanner v. 1.0 (http://www.appliedbiosystems.com); contig assembly was by BioEdit, CAP Contig Assembly Program (Hall 1999).

### Phylogenetic analysis

All new 18S sequence fragments were blasted (http://www.ncbi.nlm.nih.gov/BLAST) to determine whether they were from a *Paraphysomonas* culture or a non-scaly *Spumella* before spending time fixing cells for TEM. Unidentified environmental sequences related to *Paraphysomonas* were obtained from GenBank both by BLAST-based selection and from published work on chrysophytes (Richards et al., 2005, Shi et al., 2009, Charvet et al., 2011, del Campo and Massana, 2011, Tarbe et al., 2011). A very extensive alignment of over 500 18S rDNA sequences was made manually with the help of Macgde (http://macgde.bio.cmich.edu) for chrysophytes and representatives of all major heterokont outgroups, from which we selected two representative taxon samples for detailed analysis: an ochrophyte-wide alignment of 329 sequences and 1672 nucleotide positions and a smaller one restricted to 239 chrysophyte sequences plus four belonging to their closest outgroup Picophagea (1681 positions). Trees for each were calculated by RAxML v.7.0.4 (Stamatakis 2006) using the GTRGAMMAMIX model with eight rate categories and by Mr Bayes (Ronquist and Huelsenbeck, 2003) and the covarion and adgamma options with four rate categories and five million generations (1 M generations discarded as burnin).

### Fixation and transmission electron microscopy (TEM)

Fresh cultures of each strain were prepared for TEM as they have less detritus than the stock cultures; excess medium was filtered out before fixation. EM grade 25% glutaraldehyde was added directly to the filtered culture to a working concentration of 2.5%. The fix was washed after 1 h, rinsed with distilled water, again by filtration, to a final volume of ∼2 μl. The entire sample was never allowed to pass completely through the filter; vacuum pressure being released before all liquid passed through; the remaining concentrated-with-cells fluid was recovered by disposable pipette. Cells were allowed to settle before pipetting ∼8 μl of the concentrate (from the bottom of the Eppendorf tube) onto a formvar-coated 200 mesh copper grid. The sample was allowed to practically dry-out, then washed in distilled water. Samples were viewed as unstained whole mounts with an FEI Tecnai 12 electron microscope.

### Light microscopy

All cultures were recorded live using Sony HDV 1080 Handycam^®^ via an adapter fitted to Nikon Eclipse 80i microscope and viewed using a differential interference contrast water immersion lens (X60 NA 1.0). Cell measurements were all made by videoing live specimens and calibrating the measurements by a micrometer scale videoed using the same settings. Video footage was uploaded to computer using FinalCut Express HD 3.5.1 from which still images were exported and transferred to Adobe Photoshop CS4 11.0.2 to make plates.

## Results

About 75 putative *Paraphysomonas* cultures were obtained from freshwater, soil, and marine environments. We sequenced 18S rDNA for phylogenetic analysis from 59 genetically distinct cultures, measured cells and cilia in the light microscope, and took transmission electron micrographs of scale structure using whole mounts. All cultures but one examined ultrastructurally had simple spine scales with a broad, almost flat, usually circular unperforated base plate and relatively slender unbranched central spine broadly like those of *P. vestita* as interpreted by Korshikov (1929) and Manton and Leedale (1961) or *P. imperforata* (Lucas 1967). Yet their genetic diversity was huge and scale structure differs in fine details between strains of different sequence, so these are not merely two species. We found just one *Spumella* sp. (JQ967332 strain CH3). Relative dimensions and detailed structure of *Paraphysomonas* scales’ base-plate and spine varied systematically amongst strains in ways that correlate with their position on the tree, enabling us to establish 19 new species with spine scales from our observations on clonal cultures plus four more by reinterpreting existing literature. The culture lacking spine scales had latticed plate and crown scales and was identified as *Paraphysomonas butcheri*, here sequenced for the first time and transferred to the new genus *Clathromonas* along with 29 other (former *Paraphysomonas*) species, to which we add two further species by reinterpreting the literature that incorrectly identified them as *P. butcheri*.

## Phylogeny

Phylogenetic analyses used a large alignment with 329 ochrophyte 18S rDNA sequences including 239 chrysophytes in the hope that we could not only see where ‘*Paraphysomonas*’ sequences branch within Chrysophyceae but also clarify the uncertain relationships of the chrysophyte orders and positions of chrysophyte-related environmental DNA sequences.

**Fig. 1:**
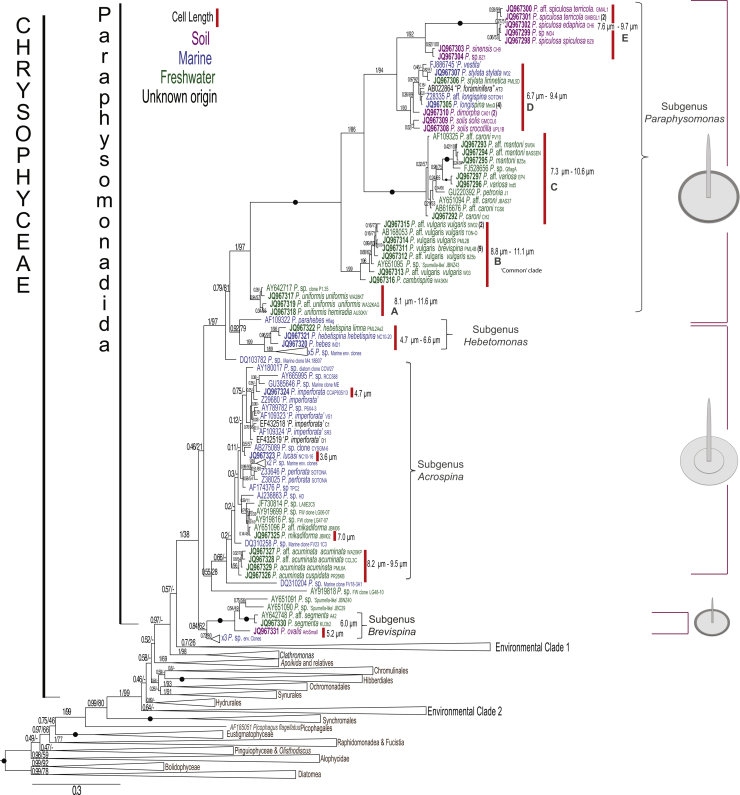
MrBayes covarion tree for 329 ochrophyte 18S rDNA sequences showing only the branching order of *Paraphysomonas* sensu stricto in detail (1672 nucleotide positions). Support values are MrBayes posterior probabilities (left) and RAxML bootstrap percentages for 1000 pseudoreplicates to the right. Black dots mean maximal support for both, i.e. 1/100. All new sequences are in bold type (starting with ‘JQ’). The number of identical sequences obtained in this study from different isolates is shown in parentheses; the common clade included the most commonly found 18S rDNA sequence. The schematic sketches indicate typical scale structure for each of the four subgenera, each corresponding to a single reproducible clade; note how isolates with dense rim to the base-plate of the spine scale group separately from those lacking a prominent rim; scale sizes are arbitrary. The ranges of cell length measurements (from this study only) are indicated beside the red lines. Sequences from freshwater strains are green, from marine strains blue, and soil strains purple. Branching order within the collapsed non-*Paraphysomona*s chrysophyte taxa are shown in Supplementary Fig. S3, and the outgroups in S2.

Fig. 1 shows that the large *Paraphysomonas* clade is maximally supported as a clade on the Bayesian tree but only very weakly by maximum likelihood (ML); it has four major subclades of distinctly different scale structure, treated here as subgenera, plus four sparsely represented environmental lineages of unknown scale structure (three with only one sequence) that branch outside them. Subgenera *Paraphysomonas* and *Hebetomonas* are each a consistently strongly supported clade in both Bayesian posterior probability (PP) and ML bootstrap (BS) support (PP 1/BS 97% and PP 0.92/BS 79%, respectively). They are sisters with strong support (0.79/81); this joint clade is sister to subgenus *Acrospina*, but this relationship is not strongly supported (PP 0.46/BS 21%). Subgenus *Brevispina* is the most divergent. Three of the four deeply branching environmental lineages are specifically related to subgenera *Hebetomonas* and *Acrospina* and thus likely to be of similar phenotype; one marine clone DQ103782 is robustly sister to the *Paraphysomonas*/*Hebetomonas* clade*.*

The largest clade (subgenus *Paraphysomonas*) includes 13 new named species that have spine scales with unperforated base-plates with a dense margin and relatively long, typically simply pointed, spines similar to those lumped in the literature as *P. vestita* from Houwink (1952) and Manton and Leedale (1961) onwards. Clearly their genetic diversity is immensely greater than can reasonably be accommodated in one species. This large clade has five speciose major subclades (A – E), whose relative branching order is robust and strongly supported by both methods; the three basal subclades (A – C) are all exclusively freshwater, suggesting that was the ancestral habitat for subgenus *Paraphysomonas*. The two derived subclades (D, E) with somewhat shorter spines are robustly sisters; subclade E, itself with two robust subclades, is exclusively from soil and subclade D has a mixture of soil, freshwater and marine species. The two marine isolates in D previously identified as *P. vestita* are almost certainly misidentified (FJ886745/Z28335, see discussion). They are genetically different from each other and both extremely distant from the third freshwater *P. vestita*, now *P*. aff. *caroni* (subclade C); the authors did not specify which strain (PV10 or DB1) was used for TEM (Lim et al., 2001), but their picture shows a scale with a spine of 2.9 μm and a base-plate of 1.4 μm. In subclade D “*P. foraminifera*” is probably also misidentified as *P. foraminifera* scale base-plates lack a dense margin and are multi-perforated (Lucas 1967) unlike any of the 13 species in subgenus *Paraphysomonas* that we sequenced and studied ultrastructurally. Subclades B – E with very long branches all share numerous insertions in 18S rDNA absent from other *Paraphysomonas* (and chrysophytes), exemplifying a common correlation between extra-rapid sequence substitution and insertionally expanded molecules (von der Heyden et al., 2004); they share a common sequence signature AT (*P. vulgaris brevispina* (strain PML4B pos. 762-763) where all other Chrysophyceae in this alignment have TC. 18S rDNA sequence signatures were also found for the two smaller *Paraphysomonas* subgenera (see taxonomy section).

Sister to the major long-spine, dense-margin clade (subgenus *Paraphysomonas*) is a small predominantly (probably ancestrally) marine clade (subgenus *Hebetomonas*) with relatively small cells and dramatically smaller scales, whose shorter spines are always blunt-ended and emerge centrally from comparatively narrower base-plates. The *Hebetomonas* clade has five marine environmental sequences and three new marine species plus a new freshwater subspecies of one of them); the *P. hebes* subclade of two new species lacks a dense base-plate margin but sometimes has a faint annular fold on the base-plate absent from subgenus *Paraphysomonas* or *P. parahebes*.

The second most speciose clade (subgenus *Acrospina*) comprises species lacking a dense base-plate margin, and whose base-plate that is either imperforate (most species, formerly lumped as *P. imperforata*) or with numerous holes (species formerly lumped as *P. foraminifera*). The *Acrospina* clade is predominantly marine, but has two substantial phyletically distinct freshwater subclades. Strains with a perforate base-plate form a small subclade within the predominantly and almost certainly ancestrally imperforate lineages. This large clade is mostly short-spined, spines barely tapering with a short dull to rounded tip, but the deep-branching *P. acuminata* subclade has characteristically long barely tapering spines with short very pointed tip (as long as in subgenus *Paraphysomonas*). The fourth morphologically defined subclade (subgenus *Brevispina*) consists of freshwater or soil lineages (e.g. *P. ovalis*, *P. segmentata*) with small cells and scales, short spines, and dense base-plate margins. Thus, three clades have dense margined base-plates (subgenera *Paraphysomonas* and *Brevispina*, and *P. parahebes*) and two have plain base-plate margins (subgenus *Acrospina* and the main subclade of subgenus *Hebetomonas*). It is not possible to decide which of these states is ancestral for *Paraphysomonas* sensu stricto.

**Fig. 2:**
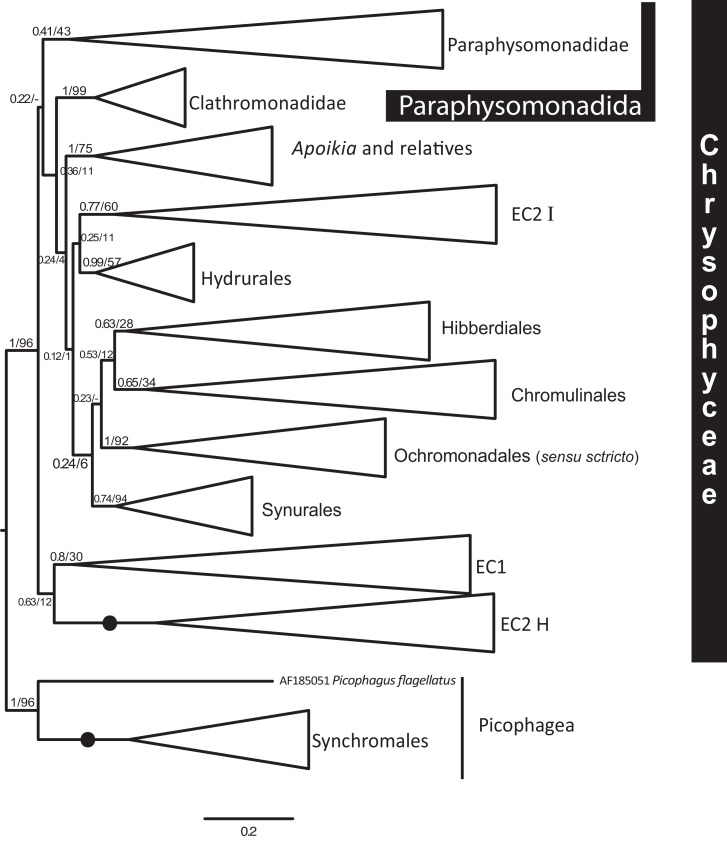
MrBayes covarion tree for 239 chrysophyte 18S rDNA sequences (1681 nucleotide positions). To emphasize the tree's main features, and fit it onto one page, internal branches of all major clades are collapsed. Support values are MrBayes posterior probabilities (left) and RAxML bootstrap percentages for 1000 pseudoreplicates to the right. Black dots mean maximal support for both, i.e. 1/100. Rooted on Picophagea (Synchromales and *Picophagus*)*.*

To test whether the poorly supported basal chrysophyte tree topology is sensitive to taxon sampling amongst ochrophyte outgroups, we also ran chrysophyte-only trees (Fig. 2) after removing the most distant 90 outgroup taxa, i.e. all except *Picophagus flagellatus* and Synchromales, leaving 239 Chrysophyceae. This did not significantly affect the internal branching order of most chrysophyte clades (not shown) or the monophyly and separateness of both Paraphysomonadidae and Clathromonadidae, but it did disrupt the previously robust grouping of Paraphysomonadidae, environmental clade 1 (EC1), and Clathromonadidae, and caused environmental clade 2 (EC2) to split into two subclades (Fig. 2). Subclade EC2H remained at the base of Chrysophyceae, where EC1 joined it to form a new weakly supported joint clade; EC2H, moved slightly to become weakly sister to Hydrurales (0.25/11), no longer the deepest branching order of Chrysophyceae. This instability to outgroup taxon sampling means that we cannot say whether EC2H is sister to EC2I (Supplementary Fig. S3) or to EC1 (Fig. 2), or whether Paraphysomonadidae and Clathromonadidae are really mutually related as Supplementary Fig. S1 indicated. In either case, there are only two major environmental clades. The apparently large difference in branching order of Fig. 2 and Supplementary Fig. S1 is deceptive, the main problem being not conflicting tree topology within Chrysophyceae but correctly determining its root.

We suspect that Supplementary Fig. S1 with its more extensive and balanced outgroup selection may be closer to the truth for the chrysophyte root position and that the apparent rearrangement of its deepest branches in Fig. 1 may arise from long-branch attraction of EC1 and Paraphysomonadidae towards the base of the tree by the remaining sparsely sampled picophagean outgroups; multigene trees are required to test this. If this interpretation is correct, Paraphysomonadida as circumscribed here is probably holophyletic and probably includes EC1 (possibly also EC2H). In both Fig. 2 and Supplementary Fig. S1 Hibberdiales and Chromulinales are sisters and group with Ochromonadales and Synurales as a weakly supported four-order clade; exclusion of both Paraphysomonadidae and Clathromonadidae from this reproducible clade is consistent with the exclusion of Paraphysomonadida from both Chromulinales and Ochromonadales as a distinct non-photosynthetic order (Cavalier-Smith and Chao 1996). The aforementioned sister relationsip between Hibberdiales and Chromulinales is only recovered by Bayes and never ML methods in Fig. 1, so these methods never agree even with more distant outgroups (Supplementary Fig. 3). And they remain contradictory with respect to the possible sister relationship between Synurales and Ochromonadales sensu stricto.

Supplementary Fig. S1 shows the large-scale structure of the chrysophyte tree: Chrysophyceae has 10 major clades, eight including known organisms and two only exclusively environmental sequences of unknown phenotype. Six organismally defined clades are predominantly (for Hydrurales entirely) algal (i.e. photosynthetic), whereas two comprise purely heterotrophic scaly phagotrophs: Paraphysomonadidae and Clathromonadidae. Paraphysomonadidae, Clathromonadidae, and environmental clade 1 form a robust clade (Paraphysomonadida) in both Bayesian and maximum likelihood (ML) analyses, but the relative branching order of these three is essentially unresolved although both methods weakly place environmental clade 1, not Clathromonadidae, as sister to Paraphysomonadidae. The branching order of Paraphysomonadida, the other seven orders, and environmental DNA clade 2 is weakly supported and inconsistent between methods. Thus, single-gene analysis is inadequate to establish the basal branching in Chrysophyceae, even though seven orders were consistently monophyletic, several with strong support. Moreover, the purely photosynthetic, scale-bearing Synurales branches within Chrysophyceae and is thus not sister to all the other branches. Clathromonadidae is a strongly supported clade in both Bayesian and ML trees. With this large taxon sample environmental clade 2 invariably groups strongly with Chrysophyceae, and Chrysophyceae are consistently sisters to Synchromales, with Picophagea apparently paraphyletic. Individual clades are collapsed in Supplementary Fig. S1 to emphasise overall tree structure. The internal branching order of Paraphysomonadidae is shown in Fig. 1 and of Clathromonadidae and all other chrysophyte clades in Supplementary Fig. S2; the internal branching order of all outgroups is in Supplementary Fig. S3.

### Taxonomy: revised classification of Paraphysomonadida

The chrysomonad order Paraphysomonadales Cavalier-Smith, 1996 was established to include both *Paraphysomonas* and *Spumella* (Cavalier-Smith and Chao 1996), but the polyphyletic *Spumella* was transferred to Ochromonadales in the light of sequence evidence that all *Spumella* clades nest within photosynthetic Ochromonadales and none are related to *Paraphysomonas* (Cavalier-Smith and Chao 2006). Thereafter Paraphysomonadales included just the family Paraphysomonadidae (=Paraphysomonadaceae) Preisig and Hibberd, 1983. This family is often included in Chromulinales, but our trees reproducibly confirm earlier sequence evidence showing that to be incorrect (Andersen, 2007, Cavalier-Smith and Chao, 2006), and that *Paraphysomonas* and *Clathromonas* are distinct deep-branching clades of Chrysophyceae, both genetically more distant from Chromulinales than is the photosynthetic scale-bearing Synurales. Thus, Paraphysomonadales clearly merits its separate ordinal status. However, especially following the inclusion of more purely phagotrophic phyla in kingdom Chromista (Cavalier-Smith 2010), the convention of treating all Chromista nomenclaturally as plants (Cavalier-Smith 1981) must be discontinued. As paraphysomonads are totally heterotrophic and protozoan-like in phagotrophic nutrition and thus not algae (Cavalier-Smith 2007), we here treat them under ICZN as order Paraphysomonadida, as phagotrophic suprageneric chromist taxa that consist exclusively or almost exclusively of heterotrophs should be treated under the zoological code of nomenclature (Cavalier-Smith 2007).

The improved classification below is based on our new ultrastructural data and sequence phylogeny jointly. Our trees showed that every *Paraphysomonas* strain with nail-like spine scales (i.e. a round or rarely oval base-plate and single unbranched slender spine) is part of a very large robust clade devoid of any strains with contrasting scale types. Here we remove all former *Paraphysomonas* species without plain spines centrally protruding from an oval or round baseplate from the genus; those that have basket and additional perforated plain plate scales are placed in a new genus, *Clathromonas*. All former *Paraphysomonas* that do not fit into either genus as defined here are assigned to new genera in a separate paper; some belong in Paraphysomonadida, others do not (Cavalier-Smith and Scoble unpublished). We now restrict family Paraphysomonadidae (=Paraphysomonadaceae) Preisig and Hibberd, 1983 to *Paraphysomonas* sensu stricto**:**

***Paraphysomonas***De Saedeleer, 1929 em.: **Revised diagnosis:** biciliate, non-amoeboid, unicellular, heterotrophic chrysomonads; cell body covered by numerous spine scales with usually circular, rarely oval, base-plate approximately orthogonal to a long thin central spine; spine unbranched, unwinged, many times narrower than base-plate even at its base; base-plate entire or with small perforations, of varying distribution but no large lacunae; spine length varies from just longer than to several times base-plate width; separate plate scales generally absent, but if present closely resemble spine-scale base-plate but with spine missing, usually larger in diameter and no distinctive morphology; slender posterior stalk anchors cell to substratum or trails behind swimming cell. Plastid a colourless leucoplast without stigma. Contractile vacuole in freshwater species. Posterior cilium lateral, much shorter than forward-directed anterior cilium. Four single nucleotide 18S rDNA signatures: A (position 1387); T (position 1465); C (position 1474); G (position 1476); all positions for reference strain ‘Arb’ *P. ovalis* (JQ967331) from the deepest clade. These sequence signatures exclude all other Chrysophyceae, except for position 1465 where one clone sequence ‘Marine Biosope T3′ (FJ537322) showed a G and all other chrysophytes A; this unique difference could be a sequencing error. Type species *P. vestita* (Stokes) De Saedeleer, 1929.

We make 23 new *Paraphysomonas* species below, including raising *P. vestita truncata* sub-species to species Preisig and Hibberd (1982a), but retain only nine existing ones in the genus: *P. vestita* (Stokes) De Saedeleer, 1929, *P. imperforata* Lucas (1967), *P. foraminifera* Lucas (1967), *P. bandaiensis* Takahashi (1976), *P. antarctica* Takahashi (1987), *P. porosa* Dürrschmidt and Cronberg (1989), *P. circumforaminifera* Wujek (1983), *P. oligocycla* Takahashi (1987), and *P. cylicophora* Leadbeater (1972) with scales with a solid base-plate bearing a perforated goblet that we postulate may be a highly modified spine scale. We now recognise 32 *Paraphysomonas* species (Table 1) and are assigning 48 former nominal *Paraphysomonas* to other genera: 29 reassigned to *Clathromonas* herein, the rest to other genera described in another paper.

**Table 1:** All known species of *Paraphysomonas* sensu stricto in alphabetical order: nine previously named species are in bold and the former subspecies now raised to species level and the other 22 are entirely novel.

1	*P. acuminata acuminata* and *acuminata cuspidata*
2	***P. antarctica***Takahashi (1987)
3	***P. bandaiensis***Takahashi (1976)
4	*P. cambrispina*
5	*P. caroni*
6	***P. circumforaminifera***Wujek (1983)
7	***P. cylicophora***Leadbeater (1972)
8	*P. dimorpha*
9	***P. foraminifera***Lucas (1967)
10	*P. hebes*
11	*P. hebetispina hebetispina* and *hebetispina limna*
12	***P. imperforata***Lucas (1967)
13	*P. longispina*
14	*P. lucasi*
15	*P. mantoni*
16	*P. mikadiforma*
17	***P. oligocycla***Takahashi (1987)
18	*P. ovalis*
19	*P. parahebes*
20	*P. perforata*
21	*P. petronia*
22	***P. porosa***Dürrschmidt and Cronberg (1989)
23	*P. segmenta*
24	*P. sinensis*
25	*P. solis solis* and *solis crocotilla*
26	*P. spiculosa* and *spiculosa edaphica* and *spiculosa terricola*
27	*P. stylata stylata* and *stylata limnetica*
28	***P**. **truncata***Preisig and Hibberd (1982a)**stat. n.**
29	*P. uniformis uniformis* and *uniformis hemiradia*
30	*P. variosa*
31	***P. vestita*** (Stokes 1885) De Saedeleer (1929)
32	*P. vulgaris vulgaris* and *vulgaris brevispina*

**New subgenus*****Paraphysomonas***De Saedeleer, 1929. **Diagnosis:** round to slightly oval unperforated base-plate with inflection at edge, edge therefore appearing denser by electron microscopy, inner annular pattern absent; central spine prominently tapers completely to a blunt or rounded tip or to a short oblique blunt tip (spine averages ≥3.2 μm and cell length typically ≥7 μm). Type species *Paraphysomonas vestita* (Stokes) De Saedeleer, 1929.

**New subgenus*****Hebetomonas*** Cavalier-Smith. **Diagnosis:** round imperforate base-plate, either inrolled at edge (appears denser by electron microscopy) or with inner annular pattern, not both; central spine barely tapering, if at all, to truncate or blunt tip (spine typically ≤1.4 μm. Cell small (typically ≤6.6 μm). Type species *Paraphysomonas hebetispina hebetispina* Scoble and Cavalier-Smith. **Etymol.** *hebes* L. blunt, referring to blunt ends of scale spines; *monas* Gk unit. **Comment**: 18S rDNA sequence signature GGTTC at position 583 – 587 of *P. hebes* (JQ967320).

**New subgenus*****Acrospina*** Cavalier-Smith. **Diagnosis:** round or oval base-plate, no obvious denser margin, sometimes with inner annular pattern; base-plate imperforate or perforated by many small holes; central spine non- or barely tapered, tip short rounded, pointed or acuminate. Wide range of cell sizes, 3.6 – 9.5 μm, and spine lengths, 0.79 – 5.4 μm. Long cilium typically more than 2.5× cell length. Type species *Paraphysomonas acuminata acuminata* Scoble and Cavalier-Smith. **Etymol.** *acer*, *acr*- L. sharp; *spina* L. thorn, because of sharp scale spines.

**New subgenus*****Brevispina*** Cavalier-Smith. **Diagnosis:** round or oval unperforated base-plate typically with denser margin, without inner annular pattern; central spine short (<1.5 μm), either non- or barely tapering spine, sometimes segmented, tip blunt. Type species *Paraphysomonas ovalis* Scoble and Cavalier-Smith. **Etymol.** *brevis* L. short; *spina* L. thorn, referring to short scale spines. **Comment**: 18S rDNA sequence signature CAAGA at position corresponding to 559 – 563 of *P. segmenta* JQ967330.

**Family Clathromonadidae** Cavalier-Smith fam. n. **Diagnosis:** As in *Paraphysomonas*, cells stalked, non-photosynthetic, with leucoplast, without stigma, but differing in scale structure. Scales non-perforated dishes with narrow margins or (more often) one or two types of open meshwork scales. Simple spine scales with entire bases absent, unlike most *Paraphysomonas;* perforated spine scales if present (rarely) never the sole scale type as in *Paraphysomonas*, but have an open-mesh base-plate, unlike the numerous small perforations of *Paraphysomonas foraminifera*, as well as a meshwork broad base to the spine itself. Type genus ***Clathromonas*** **gen. n. Diagnosis:** usually with two types of scales: inner holey plate scales, round to oval, with large holes of varied shape relative to intervening material; more complex three dimensional, basket-like scales built of a very open meshwork, of varied shapes, often present in addition to or instead of holey plate scales – these may be crown scales, chair-like or tower-like. In one species with dimorphic scales (*diademifera*) plate scales unperforated. **Etymol:** *clathri* L. lattice; *monas* Gk. unit. Type species *Clathromonas butcheri* comb. n. basionym *Paraphysomonas butcheri* (Pennick and Clarke 1972).

We make 28 other new combinations for former *Paraphysomonas* and describe two new species, making 31 *Clathromonas* species in all; at least 10 are known to have leucoplasts:

*Clathromonas bisorbulina* comb. n. basionym *Paraphysomonas bisorbulina* (Yu et al. 1993). Yu et al. (1993) compared *C. bisorbulina* to *stephanolepis*, which has one type of crown/basket scale and no baseplate was shown intact, unlike what Yu et al. (1993) suggested for *P. bisorbulina*. We think that the ‘broken’ ‘spines’ (struts) reported by Yu et al. (1993) are actually broken crown/basket scales fallen alongside a distinct plate scale, which they misinterpreted as a ‘base-plate’ of a spine scale. Plate 2D, E are poor images of scales, but F, G and H are clear and show a plate scale separate from a broken basket scale. Gao et al. (1993) misinterpret the description of *C. stephanolepis*, stating ‘the scales of *P. stephanolepis* have only base-plates and no apical plate’, which is wrong because they are basket-like. In Yu et al. (1993) the schematic Fig. 2 legend is confused; Fig. 2K is actually *P. simplexocorbita* and Fig. 2M is *P. bisorbida.* The TEM images of *C. bisorbulina* seem most similar to *P. butcheri* of Thomsen 1975 (their Figures 16 – 19), which has separate plate scales and crown/basket scales. Plate scales of *C. bisorbulina* resemble those of *C. homolepis* (Preisig and Hibberd 1982a, particularly Fig. 1E).

*Clathromonas cancellata* comb. n. basionym *Paraphysomonas cancellata* (Preisig and Hibberd 1982b)

*Clathromonas canistrum* comb. n. basionym *Paraphysomonas canistrum* (Preisig and Hibberd 1982b). Leucoplast.

*Clathromonas corbidifera* comb. n. basionym *Paraphysomonas corbidifera* (Pennick and Clarke 1973)

*Clathromonas coronata* comb. n. basionym *Paraphysomonas coronata* Moestrup and Zimmerman in (Thomsen et al. 1981)

*Clathromonas cribosa* comb. n. basionym *Paraphysomonas cribosa* (Lucas 1968)

*Clathromonas diademifera* comb. n. basionym *Ochromonas diademifera* (Takahashi, 1972). Synonyms *Lepidochromonas diademifera* Kristiansen, 1980; *Paraphysomonas diademifera* (Preisig and Hibberd 1982a). Leucoplast.

*Clathromonas eiffellii* comb. n. basionym *Paraphysomonas eiffellii* Thomsen in (Thomsen et al. 1981)

*Clathromonas elegantissima* comb. n. basionym *Paraphysomonas elegantissima* (Kling and Kristiansen 1983)

*Clathromonas faveolata* comb. n. basionym *Paraphysomonas faveolata* (Rees et al. 1974)

*Clathromonas homolepis* comb. n. basionym *Paraphysomonas homolepis* (Preisig and Hibberd 1982b)

*Clathromonas ignivoma* comb. n. basionym *Paraphysomonas ignivoma* (Preisig and Hibberd 1982b). Leucoplast.

*Clathromonas inconspicua* comb. n. basionym *Paraphysomonas inconspicua* (Takahashi 1976). We do not accept its synonymization with *P. butcheri* (Preisig and Hibberd 1982b), though agree that interpretation of crown scale structure is not easy (they appear to differ); its plate scales are very distinct, with much greater contrast between large holes and tiny ones than in *C. butcheri*. Moreover, *C. inconspicua* is from freshwater, not brackish like *C. butcheri* (however *P. butcheri* from Cambridgeshire freshwater ponds (Preisig and Hibberd 1982b) seems correctly identified and is very similar to our brackish *C. butcheri* strain – see below). We agree with Preisig and Hibberd (1982b) that *P. butcheri* of Takahashi (1976) was misidentified, as was his *P. foraminifera*; however we do not accept that Takahashi's ‘*butcheri*’ was *P. morchella*, as the small-mesh holes of *morchella* were much less evident; it may be an undescribed species somewhat similar to *P. morchella* with a less evident chair-back and fewer small holes.

*Clathromonas manubriata* comb. n. basionym *Paraphysomonas manubriata* (Preisig and Hibberd 1982b) stat. n. (Vørs et al. 1990)

*Clathromonas morchella* comb. n. basionym *Paraphysomonas morchella* (Preisig and Hibberd 1982b). Leucoplast.

**Fig. 3:**
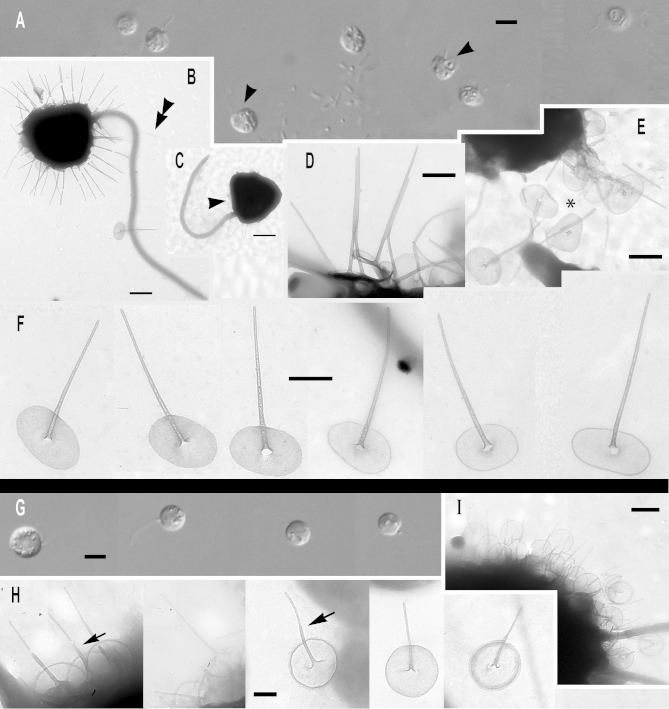
Micrographs of two new species of subgenus *Brevispina*: *Paraphysomonas ovalis, P. segmenta.***(A – F)***P. ovalis*, **(G – I)***P. segmenta*. **(A)** DIC light micrographs of live *P. ovalis* cells. **(B – F)***P. ovalis* TEMs. **(B)** Mastigonemes (double arrowheads) visible on whole cell. **(C)** Flattened side of cell at base of cilia (arrowhead). **(D)** Side view of spine scales near cell surface. **(E)** Aberrant base-plate forms (*). **(F)** Single scales showing variation in density of base-plate rim. **(G)** DIC of live *P. segmenta* cells. **(H)** Scales, showing the ‘shoulder’ of thickened shaft as it meets thinner end of spine (arrow). Thickness of prominent dense base-plate margin is variable. **(I)** Scales on surface of cell. Scale bars: A and G, 5 μm. B and C, 1 μm. H, 0.2 μm. D – F and I, 0.5 μm.

**Fig. 4:**
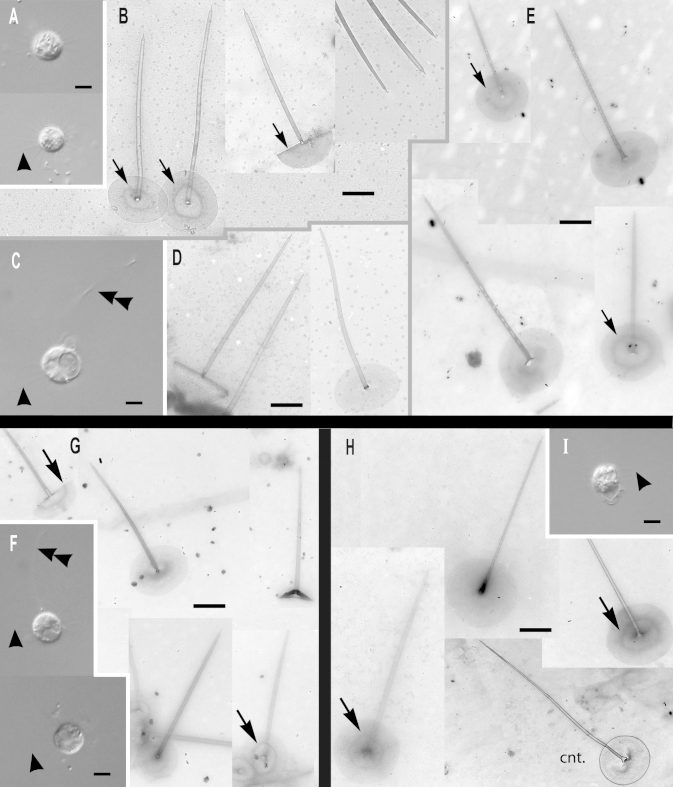
Micrographs of three new species of subgenus *Acrospina: Paraphysomonas acuminata acuminata*, *P. acuminata cuspidata*, *P. mikadiforma*. **(A,B)***P. a. acuminata*. (PML6A). **(C,D)***P.* aff. *a. acuminata* (WA20KP). **(E)***P.* aff. *a. acuminata*. (CCL3C). **(F,G)***P. a. cuspidata* (PR26KB). **(H,I)***P. mikadiforma*. **(A)** DIC live cells of *P. a. acuminata*, spines visible (arrowhead). **(B)** TEM of spine scales with oblique pointed tip and dense annulus on base-plate (arrows). **(C)** DIC live cell *P.* aff. *a. acuminata* (WA20KP), spines visible (arrowhead) and typical long posterior cilium. **(D)** TEM of spine scales with no clear annulus visible. **(E)***P.* aff. *a. acuminata* (CCL3C) TEM of scales, showing annulus (arrowhead). **(F)** DIC of *P. a. cuspidata*, long AF (double arrowhead) and spines visible (arrowhead). **(G)** TEM of spine scales with oblique pointed tip and annulus on base-plate (arrow). **(H)** TEM of three spine scales of *P. mikadiforma* with no dense base-plate rim and one contaminant scale (cnt.) with dense margin and different spine tip. Annulus clearly visible on two scales (arrows). **(I)** DIC of live *P. mikadiforma* with visible spines (arrowhead). Scale bars; A, C, F and I, 5 μm. B, D, E, G and H, 1 μm.

**Fig. 5:**
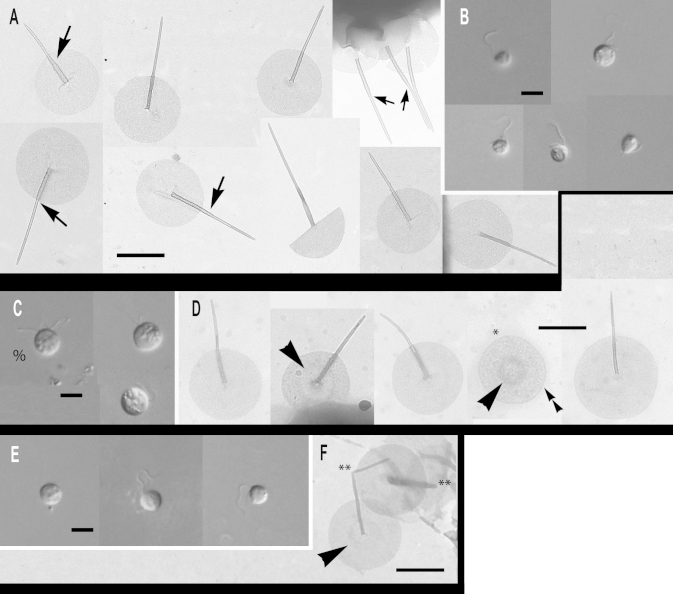
Further micrographs of subgenus *Acrospina*: *Paraphysomonas lucasi, P.* aff*. imperforata* (EP1) and *P. imperforata* (CCAP 935/13). **(A, B)***P. lucasi*. **(C, D)***P.* aff. *imperforata* (EP1). **(E,F)***P. imperforata* (CCAP 935/13). **(A)** TEM of detached scales showing the point at which the spine thins to a slight shoulder (arrow). **(B)** DIC of live *P. lucasi* cells. **(C)** DIC of live EP1 and larger dividing cell, shows four cilia (%). **(D)** TEM EP1 scales showing annulus (arrowhead) and one uncommon scale with very faintly denser rim on the base-plate (double arrowhead). **(E)** DIC of CCAP 935/13. **(F)** Scale showing base-plate annulus (arrowhead), broken spines (**). Scale bars: A – F, 0.5 μm.

**Fig. 6:**
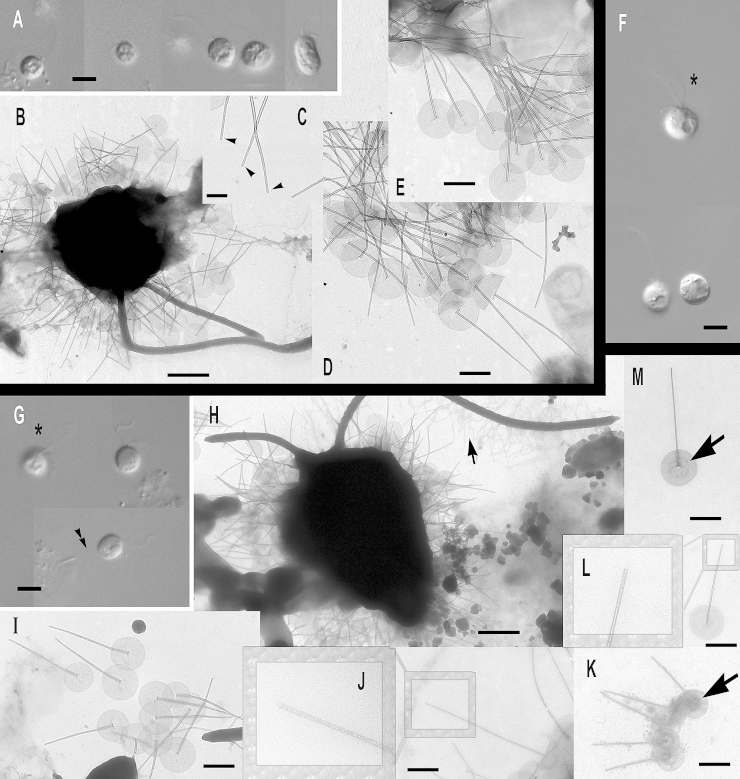
Micrographs of two new species of subgenus *Hebetomonas: Paraphysomonas hebes*, *P. hebetispina limna*, *P. hebetispina hebetispina*. **(A – E)***P. hebes*. **(F)***P. hebetispina limna*. **(G – M)***P. hebetispina hebetispina*. **(A)** DIC of live *P. hebes* cells. **(B – E)***P. hebes* TEMs. **(B)** Whole cell with both cilia and scales. **(C)** Blunt tips of scales (arrowhead). **(D, E)** Typical P. hebes scales. **(F)** DIC of live *P. h. limna* cells, one with obvious beating envelope (*). **(G)** DIC of live *P. h. hebetispina* cells, one with visible stalk (double arrowhead) and another with beating envelope (*). **(H – M)***P. h. hebetispina* TEMs. **(H)** Whole cell showing mastigonemes (small arrow), both cilia and scales. **(I)** Loose scales showing range of size, gentle taper of spine, and base-plate with no dense rim. **(J)** Close-up of blunt tip of spine. **(K)** A selection of scales showing obvious base-plate annulus (large arrow). **(L)** Another example of blunt tip of spine. **(M)** Single scale with base-plate annulus (large arrow). Scale bars: A, F and G, 5 μm. B and H, 1 μm. C, 0.2 μm. D, E, I, J, K, L, and M, 0.5 μm.

**Fig. 7:**
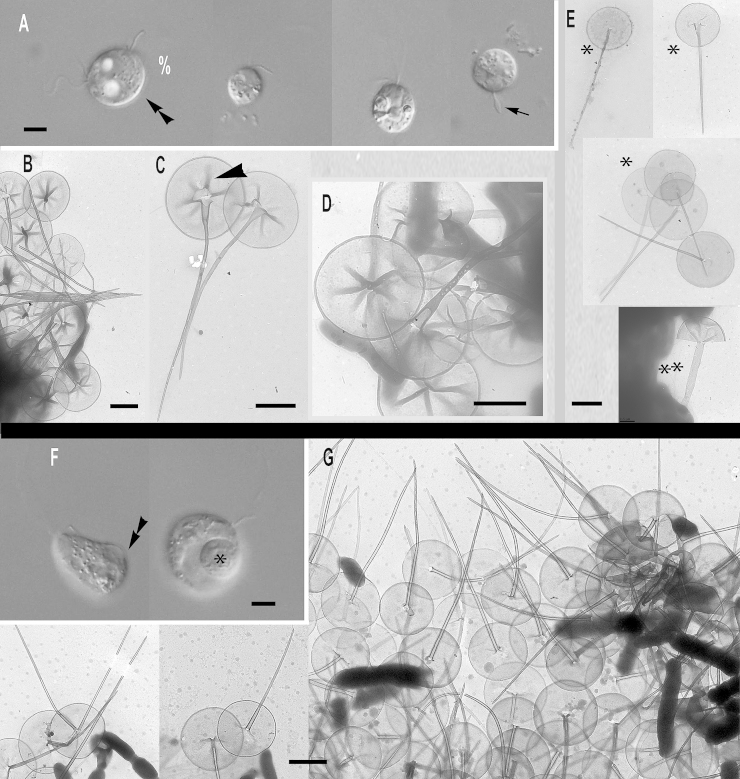
Micrographs of new species of the most divergent subclade of subgenus *Paraphysomonas*: *Paraphysomonas uniformis hemiradia, P. uniformis uniformis.***(A – E)***P. u. hemiradia*. **(F, G)***P. u. uniformis*. **(A)** DIC of live *P. u. hemiradia*. Scale-base layer visible (double arrowhead) on dividing cell (%). Unknown protrusion from one cell (arrow). **(B – E)***P. u. hemiradia* TEMs. **(B)** Collection of closely grouped scales with radial ribs. **(C)** Two scales with radial ribs (arrowhead). **(D)** Close up of base-plate with radial ribs. **(E)** A selection of aberrant scale-types without radial ribs (*) and one with radial ribs and a very wide spine (**). **(F)** DIC of live *P. u. uniformis*, pyriform cell with visible scale-base layer (double arrowhead), large cell phagocytosed possibly smaller *Paraphysomonas* cell (*). **(G)** TEM of *P. u. uniformis.* Detached scales. Scale bars: A and F, 5 μm. B, C, D, E and G, 1 μm.

**Fig. 8:**
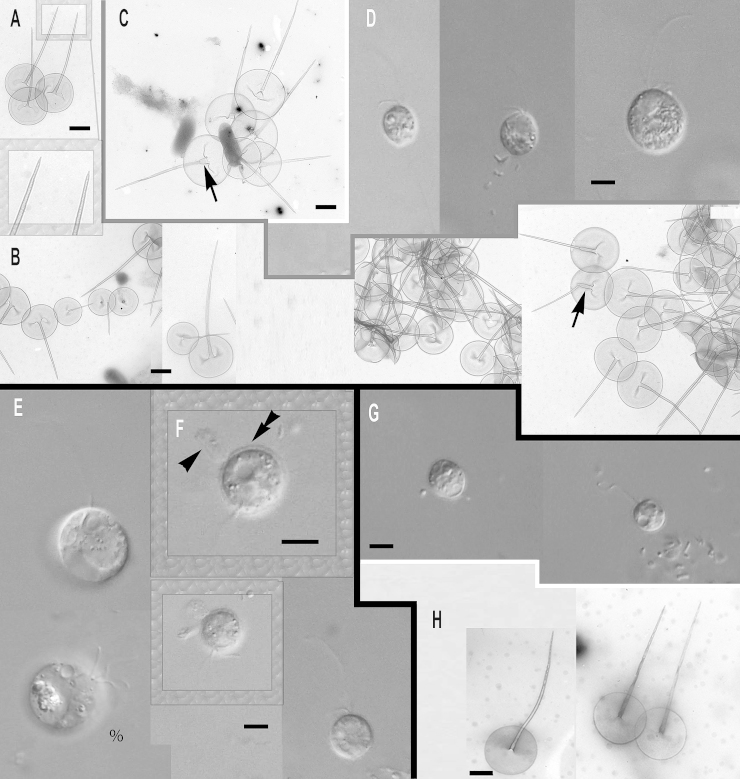
Micrographs of two new species of the freshwater ‘common’ subclade of subgenus *Paraphysomonas*: *Paraphysomonas vulgaris vulgaris, P.* aff. *vulgaris vulgaris* (W03)*, P. cambrispina.***(A, B)***P.* aff. *v. vulgaris* (SW02). **(C, D)***P. v. vulgaris*. **(E, F)***P. v. vulgaris* (W03). **(G, H)***P. cambrispina*. **(A, B)***P.* aff. *v. vulgaris* TEMs. **(A)** A group of detached scales with a close-up of the spine tips. **(B)** A selection of *P.* aff. *vulgaris* scales with varying spine lengths as well as inflated spine bases (arrow). **(C)***P. v. vulgaris* TEM. Detached scales with inflated spine base and crease (arrow). **(D)** DIC of live *P. vulgaris* cells of varying sizes. **(E, F)** DIC of live *P.* aff. *v. vulgaris* (W03) cells; one dividing (%). **(F)** Enlarged image of boxed cell to show obvious scale-base layer (double arrowhead) and possible extrusome (arrowhead). **(G)** DIC of live *P. cambrispina* cells. (H) TEM of detached *P. cambrispina* scales, N.B. scales are almost twice as small as the other species in this plate. Scale bars: C, D, E, F and G, 5 μm. A and B, 1 μm. H, 0.5 μm.

**Fig. 9:**
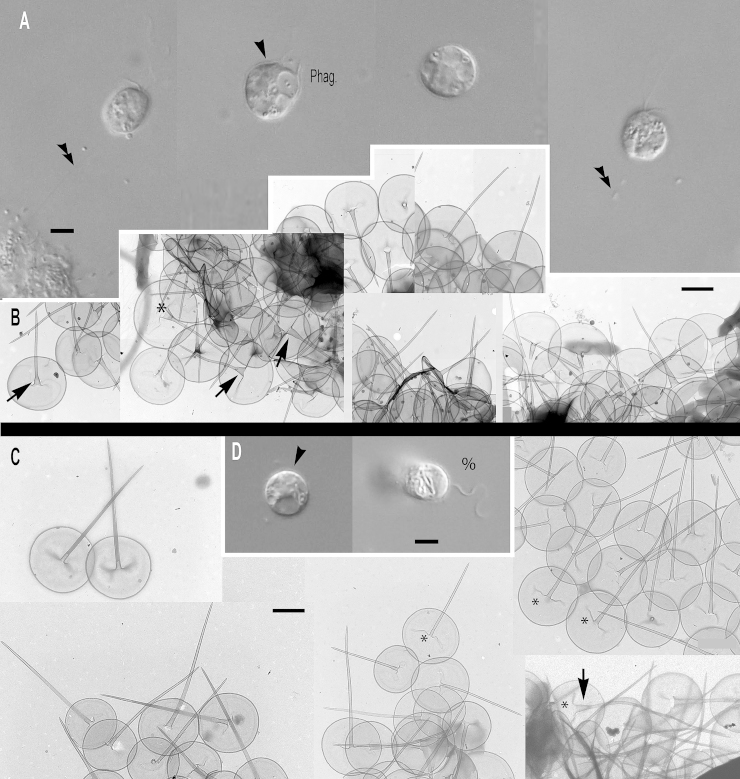
Micrographs of two *Paraphysomonas vulgaris* strains with the same 18S rDNA but contrasting spine lengths. **(A, B)***P. vulgaris brevispina*. **(C, D)***P. v. vulgaris* (PML8). **(A)** DIC of live *P. v. brevispina* cells with stalk (double arrowhead) and visible scale-base layer (arrowhead) and beginning to phagocytose (Phag.). **(B)***P. v. brevispina* TEMs showing detached scales with prominent inflated spine base (arrow) and dense margin (*). **(C)***P. v. vulgaris* (PML8) TEMs showing detached scales with base-plate crease (*) and some with inflated base of spine (arrow). **(D)** DIC of live *P. v. vulgaris* (PML8) cells showing scale-base layer (arrowhead) and dividing cell (%). Scale bar: A and D, 5 μm. B and C, 1 μm.

**Fig. 10:**
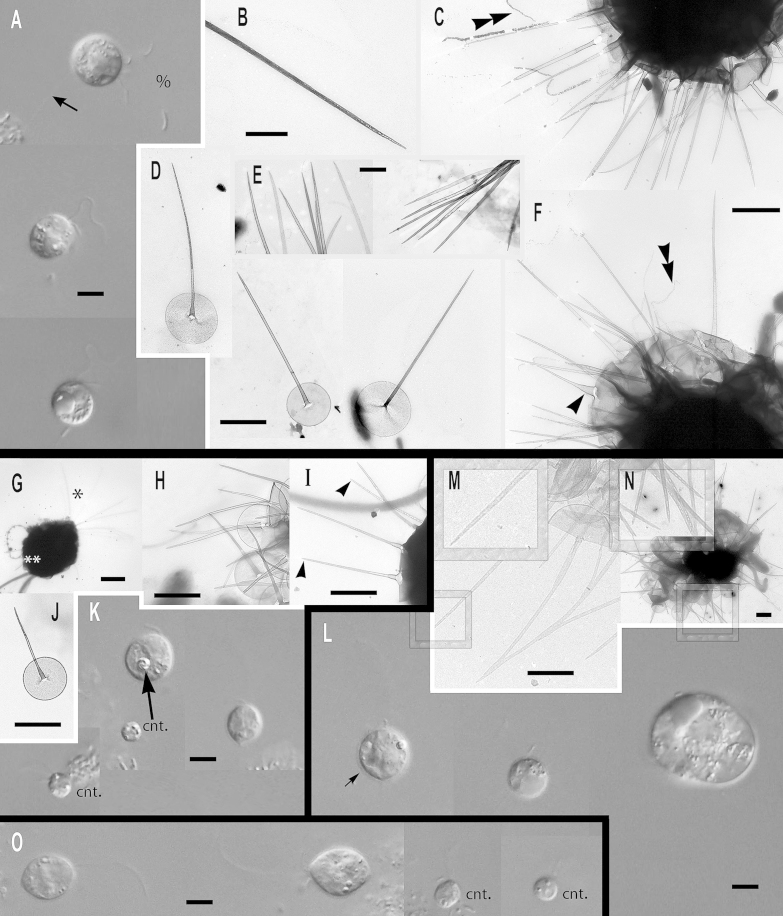
Micrographs of new species in the third freshwater clade of subgenus *Paraphysomonas*: *Paraphysomonas variosa, P. caroni, P. mantoni, P.* aff. *mantoni* (Bassen)*.***(A – F)***P. variosa*. **(G – K)***P. caroni*. **(M – L)***P. mantoni*. **(O)***P.* aff. *mantoni* (Bassen). **(A)** DIC of live *P. variosa* cells, dividing (%) and with stalk (arrow). **(B – F)***P. variosa* TEMs. **(B)** Close-up of tapering scale spine tip. **(C)** Whole cell with attached scales, possible malformed spines (double arrowhead). **(D)** Three detached scales showing diversity of size. **(E)** Close-up of tapering spine tips. **(F)** Attached scales showing detached mastigonemes or possibly malformed spines (double arrowhead) and example of shorter scale spine with wide inflated base (arrowhead). **(G – J)***P. caroni* TEMs. **(G)** Whole cell with few cells attached (**) and long filaments, possibly discharged extrusomes (*). (H) Scales at surface of cell. **(I)** Scales at surface of cell showing slight oblique point at tapered spine tip. **(J)** Detached scale. **(K)** DIC of live *P. caroni* cells and contaminant (cnt.). Contaminant cells as food (arrow). **(L)** DIC of live *P. mantoni* cells, scale-base layer visible. **(M,N)***P. mantoni* TEMs showing tapering scale tips and close-up of tapering spine tip (boxes). **(O)** DIC of live *P.* aff. *mantoni* (Bassen) cells and possible contaminant (cnt.). Scale bar: A, K, L and O, 5 μm. B and E, 0.5 μm. D, F, H, I, J, M and N, 1 μm. G, 2 μm.

**Fig. 11:**
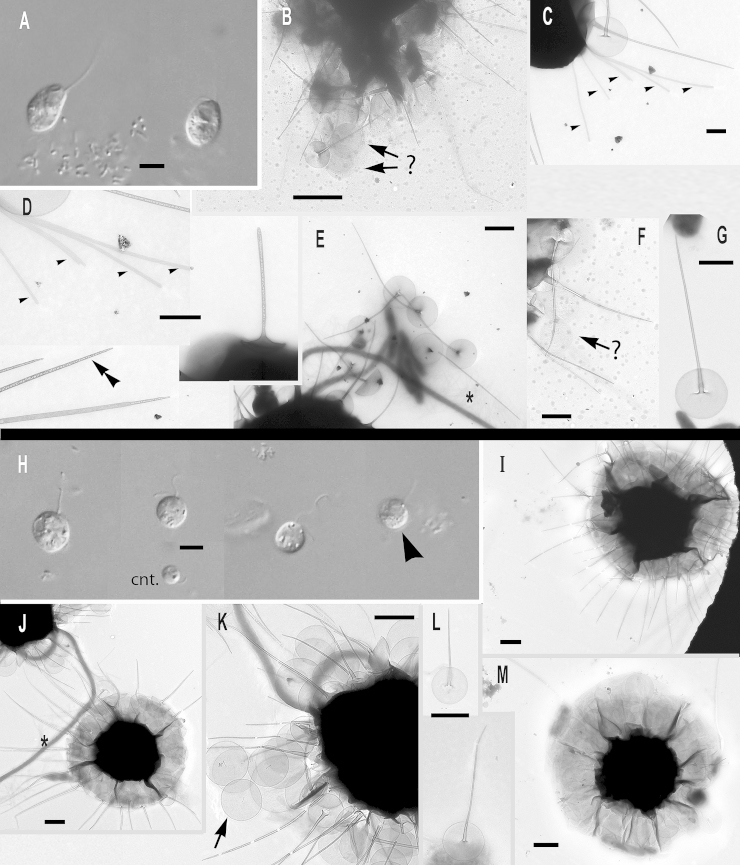
Micrographs of two new species of subgenus *Paraphysomonas* from soil: *Paraphysomonas solis crocotilla*, *P. dimorpha*. **(A – G)***P. solis crocotilla*. **(H – M)***P. dimorpha*. **(A)** DIC of live *P. solis crocotilla* cells. **(B – G)***P. solis crocotilla* TEMs. **(B)** Scales attached to cell, possible non-spine scales (arrows). **(C)** Possible extruded extrusomes (arrowheads). **(D)** Close-up of possible extrusomes and tapering tip of spine scales (double arrowhead) and aberrant scale form with bulbous tip. **(E)** Group of detached scales and visible mastigonemes (*). **(F)** Scales showing swollen spine bases; shadowy marks similar to scale base-plates possibly caused by scales washed off the grid during preparation (arrow). **(G)** Detached scale. (H) DIC of live *P. dimorpha* cells and one possible contaminant (cnt.) or dormant cell (see description), base-layer of scales visible (arrowhead). **(I – M)***P. dimorpha* TEMs. **(I)** Whole cell with apparently just spine scales. **(J)** Whole cells with a mix of spined and plate scales. Mastigonemes (*). **(K)** Detached plate scales (arrow) from whole cell. **(L)** Detached spine scales. **(M)** Whole cell with only plate scales. Scale bar: A and H, 5 μm. **(B)** 2 μm. (C and D) 0.5 μm. E, F, G, I, J, K, L and M, 1 μm.

**Fig. 12:**
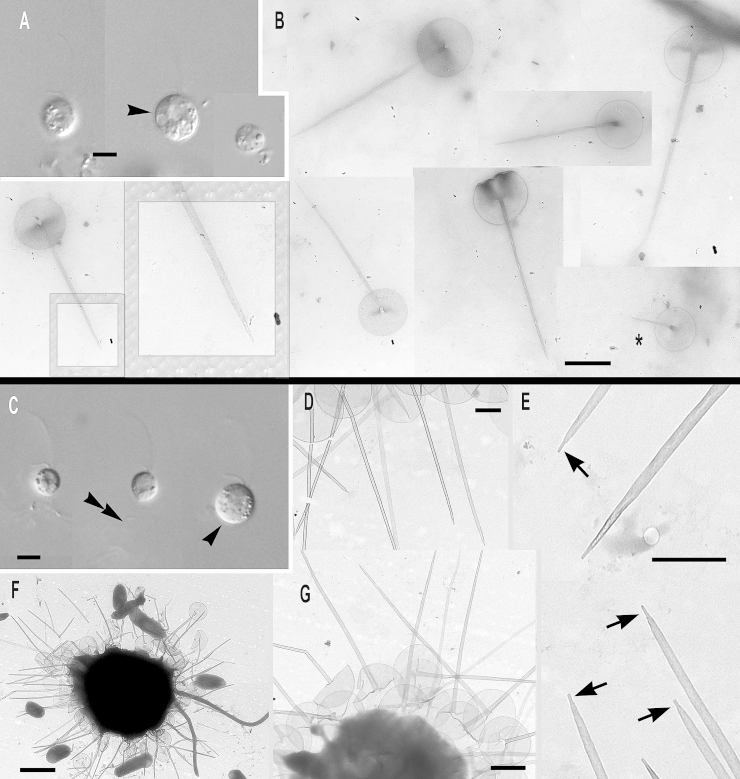
Micrographs of two new species of subgenus *Paraphysomonas* from the subclade with mixed habitat preferences: *Paraphysomonas solis solis* from soil, marine *P. stylata stylata*. **(A,B)***P. solis solis*. **(C – G)***P. stylata stylata*. **(A)** DIC of live *P. solis solis* cells one with visible scale-base layer (arrowhead). **(B)***P. solis solis* TEMs of detached scales showing tapering spine and tip (enlarged box) and aberrant small scale (*). **(C)** DIC of live *P. stylata stylata* cells showing stalk (double arrowhead) and scale-base layer (arrowhead). **(D – G)***P. stylata stylata* TEMs. **(D, G)** Scales. **(E)** Close-up of pinched spine tips (arrow). **(F)** Whole cell with scales. Scale bars: A and C, 5 μm. B and G, 1 μm. D and E, 0.5 μm. F, 2 μm.

**Fig. 13:**
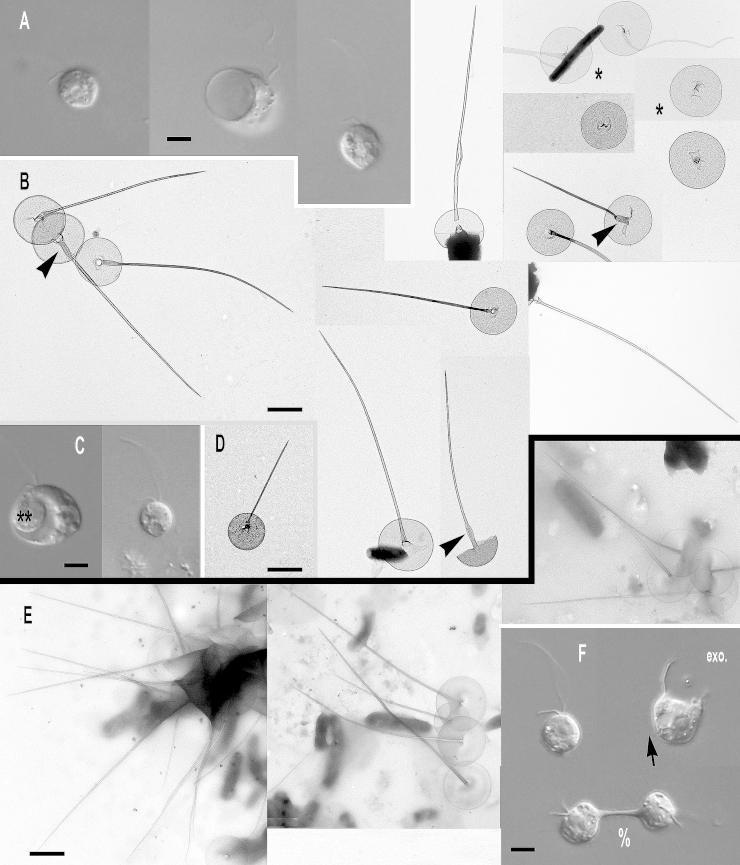
Micrographs of two further new species of subgenus *Paraphysomonas* from the subclade with mixed habitat preferences: *Paraphysomonas longispina, P. stylata limnetica*. **(A,B)***P. longispina* (Mex3 – freshwater). **(C,D)***P*. *longispina*, (Mex1 – marine). **(E,F)***P. stylata limnetica*. **(A)** DIC of *P. longispina* (Mex3) live cells. **(B)***P. longispina* (Mex3) TEMs of detached scales, some with inflated spine bases (arrowhead). Aberrant scales (*) with lost or malformed spines. **(C)** DIC of live *P*. *longispina* (Mex1) cells, one having ingested another smaller of its own kind (**), other cell attached to floating detritus via stalk. **(D)***P*. *longispina* (Mex1) TEM of a single scale. **(E)***P. stylata limnetica* TEMs of detached spine scales. **(F)** DIC of live *P. stylata limnetica* cells. Scale-base layer and spines just visible (arrow). Exocytosis observed (exo.) and late stage division (%). Scale bar: A, C and F, 5 μm. B, D and E, 1 μm.

**Fig. 14:**
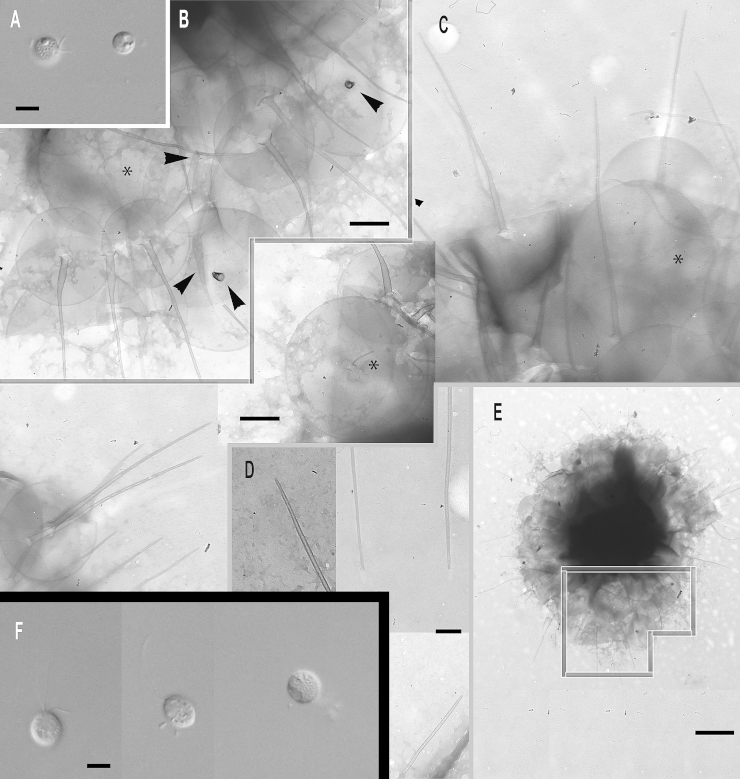
Micrographs of two closely related subspecies of *Paraphysomonas spiculosa* from small soil subclade: *Paraphysomonas sinensis, P.* sp. (BZ1). **(A – E)***P. sinensis*. **(F)***P.* sp. (BZ1)*.***(A)** DIC of *P. sinensis* culture showing granule-like cells. **(B – E)***P. sinensis* TEMs. **(B)** Scales with short spines or short protrusions (arrowheads). Large plate discs (*) with or possibly without central marking. **(C)** Spined scales and non-spined plate scale (*). **(D)** Tips of spines. **(E)** Whole cell with boxed area indicating from where Fig. 16B is taken. **(F)** DIC of live *P.* sp. (BZ1) cells. Scale bar: A and F, 5 μm. B and C, 0.5 μm. D, 0.2 μm. E, 2 μm.

**Fig. 15:**
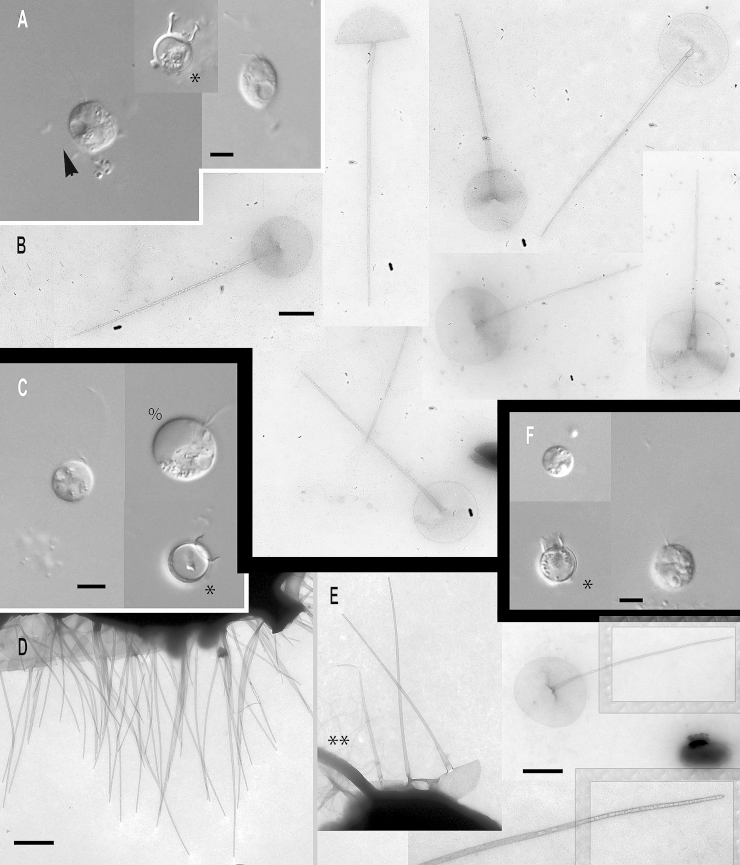
Micrographs of three closely related new species of subgenus *Paraphysomonas* from soil, showing stomatocysts of differing morphology: *Paraphysomonas spiculosa terricola, P. spiculosa spiculosa, P. spiculosa edaphica.***(A, B)***P. s. terricola*. **(C – E)***P. spiculosa spiculosa*. **(F)***P. spiculosa edaphica.***(A)** DIC of live *P. spiculosa terricola* cells with just visible scales (arrowhead) and plugged stomatocyst (*). **(B)***P. spiculosa terricola* TEMs of detached scales. **(C)** DIC of live *P. spiculosa spiculosa* cells, one dividing (%) and plugged stomatocyst (*). **(D, E)***P. spiculosa spiculosa* TEMs. **(D)** Scales attached to cell surface. **(E)** Side view of scales and cilium hairs (**) and single scale including close-up of spine tip (box). **(F)** DIC of live *P. spiculosa edaphica* motile cell with cilia (on right), smaller more shrunken cell at top left (perhaps starved, apparently non-ciliate) and stomatocyst (*). Scale bar: A, C and F, 5 μm. B, 0.5 μm. D and E, 1 μm.

*Clathromonas poteriophora* comb. n. basionym *Paraphysomonas poteriophora* Moestrup and Kristiansen in Thomsen et al. (1981). We strongly disagree with its inclusion within *C. coronata* (Vørs et al. 1990), as their scales are very distinct. We think Fig. 1, Fig. 2, Fig. 3, Fig. 4, Fig. 5, Fig. 6, Fig. 7, Fig. 8, Fig. 9, Fig. 10, Fig. 11, Fig. 12, Fig. 13, Fig. 14, Fig. 15, Fig. 16 of Vørs et al. are not *coronata*, but a third, undescribed species more closely related to *coronata* than to *poteriophora*, and are not intermediate between *coronata* and *poteriophora*, and do not justify their merger. Their claim that Preisig and Hibberd (1982b) showed intermediates is disputable; in our view, Fig. 19 I, L-O of Preisig and Hibberd (1982b) are neither *C. poteriophora*, nor intermediates between *poteriophora* and *coronata* as Vørs et al. apparently assumed, but a fourth (undescribed) species closer to *poteriophora* than to *coronata*.

*Clathromonas preisigii* comb. n. basionym *Paraphysomonas preisigii* (Wujek 2013)

*Clathromonas quadrispina* comb. n. basionym *Paraphysomonas quadrispina* Thomsen and Kristiansen in (Thomsen et al. 1981). Leucoplast.

*Clathromonas runcinifera* comb. n. basionym *Paraphysomonas runcinifera* (Preisig and Hibberd 1982b)

*Clathromonas sideriophora* comb. n. basionym *Paraphysomonas sideriophora* (Thomsen 1975)

*Clathromonas sigillifera* comb. n. basionym *Paraphysomonas sigillifera* Moestrup in Thomsen et al. (1981)

*Clathromonas simplexocorbida* comb. n. *Paraphysomonas simplexocorbida* (Yu et al. 1993)

*Clathromonas stelligera* comb. n. basionym *Paraphysomonas stelligera* (Preisig and Hibberd 1982b)

*Clathromonas stephanolepis* comb. n. basionym *Paraphysomonas stephanolepis* (Preisig and Hibberd 1982b). Leucoplast.

*Clathromonas subquadrangularis* comb. n. basionym *Paraphysomonas subquadrangularis* (Preisig and Hibberd 1982b). Leucoplast.

*Clathromonas subrotacea* comb. n. basionym *Paraphysomonas subrotacea* Thomsen in Thomsen et al. (1981). Leucoplast.

*Clathromonas takahashii* comb. n. basionym *Paraphysomonas takahashii* Cronberg and Kristiansen in Thomsen et al. (1981)

*Clathromonas undulata* comb. n. basionym *Paraphysomonas undulata* (Preisig and Hibberd 1982b). Leucoplast.

### Taxonomy: 23 new *Paraphysomonas* species, eight new subspecies, and strain descriptions

All new isolates described below are colourless biciliate cells with tubular hairs on the long undulating anterior cilium (LC) and a smooth shorter, largely passive, ‘posterior’ cilium (SC). They all swim with anterior cilium leading and a trailing stalk used to attach to the substrate when feeding (sessile). All new species had spine scales and imperforate base-plates. Diagnoses do not repeat these shared characters. Except where stated otherwise all base-plates are round. Cell length (CL) measurements and estimates of cilium length were on live cells; mean cell length is given first followed in brackets by the range and number of cells measured. Scale base-plate diameter measured across the widest point, and spine-length to plate-width ratio (S/P ratio) is important in distinguishing species. For basally thicker spines we sometimes give spine-base widths above the base-plate (not to be confused with the far greater width of the whole base-plate), average values being followed by the range in parentheses. In some strains the scale spines are visible individually on living cells in the light microscope (LM), mainly in those with unusually thick spines, but in most they are not. Even when one cannot see spines, the base-plates may collectively form a visible layer seen as a dense line around the main cell surface, which we refer to as a ‘scale-base layer’ since its LM visibility or not is constant for each strain.

Diagnoses/descriptions are grouped by species positions on the tree (Fig. 1), which usually placed those with more similar scales mutually closer. When we designate type sequences, strains, and illustrations, or any combination of these, all are to be regarded as part of a syntype (Cavalier-Smith and Chao 2010). To save space we have not prepared both comprehensive descriptions of new strains and separate diagnoses focusing solely on those characters that distinguish each species from its closest relatives. Our decisions about species boundaries were made primarily using scale ultrastructural and rDNA sequence differences, which generally mutually agree well; either or both these features (and for three species stomatocyst morphology) can be used in future to reidentify reliably all new species and distinguish them from close relatives. Features like cell size and ciliary length are included as necessary features for properly describing most new species (summarised in Table 2), but though they map in a meaningful way onto the phylogenetic tree, and therefore are more stable evolutionarily and genetically than some might have anticipated, they cannot generally be used to discriminate between close species, and are thus corroborative rather than diagnostic characters for correct identification.

**Table 2:** *Paraphysomonas* and *Clathromonas* species and strains light microscope and TEM data, new species names in bold.

**Species name (strain code)**	**GenBank 18S**	**Cell length (CL)**	**Long cilium (xCL)**	**Short cilium (xCL)**	**Spine Scale**			**Base-plate**		
					**Spine length (μm)**	**Base width (μm)**	**S/P ratio**	**Dense margin?**	**Annulus?**	**Spine tip shape**
***P. (B.) ovalis*** (ARB)	JQ967331	5.2	**1.5 – 2**	**0.75**	1.5	0.8	1.9	y	n	Rounded
***P. (B.) segmenta*** (Ku3b2)	JQ967330	6.0	**2**	**0.5**	0.65	0.44	1.4	y	n	Rounded
*P.* (*A*.) *imperforata*, Lucas 1967	/	4.5	**3 – 4**	**1**	1.0	0.77	1.3	n	y	Unknown
*P.* (*A*.) aff*. imperforata* (CCAP 935/13)	Identical to EF4232518 (C1) pos. 70-737	4.5	**3 – 3.5**	**1**	1.1	0.8	1.3	n	y/n	Rounded
*P.* (*A*.) aff*. imperforata* (EP1)		4.7	**2.5**	**0.75 – 1**	0.79	0.71	1.1	n	y/n	Rounded
***P.*****(*****A*****.)*****lucasi*** (NC10-16)	JQ967323	3.6	**2.5 – 3**	**0.75**	0.92	0.7	1.3	n	n	Rounded
***P. (A.) mikadiforma*** (JBM02)	JQ967325	7.0	**3.5 – 5.0**	**1**	5.2	2.1	2.5	n	y/n	Acutely pointed
***P. (A.) acuminata acuminata*** (PML6A)	JQ967329	9.0	**3.5 – 4**	**0.75 – 1**	5.2	1.9	2.8	n	y	Acutely pointed
***P. (A.) acuminata cuspidata*** (PR26KB)	JQ967326	9.2	**2.5 – 3**	**0.75**	4.7	1.6	3.0	n	y	Acutely pointed
*P.* (*A*.) aff. *acuminata acuminata* (CCL3C)	JQ967328	8.2	**3.5**	**0.75**	5.4	2.1	2.6	n	y/n	Acutely pointed
*P.* (*A*.) aff*. acuminata acuminata* (WA20KP)	JQ967327	9.5	**3.5**	**0.75**	5.3	2	2.6	n	y/n	Acutely pointed
***P. (H.) hebes*** (Ind1)	JQ967320	4.7	**2.0**	**0.75**	1.4	0.6	2.4	n/y	n	Truncate
***P. (H.) hebetispina hebetispina*** (NC10-20)	JQ967321	5.3	**2**	**0.5 – 0.75**	1.2	0.5	2.5	n/y	y/n	Truncate slightly rounded
***P. (H.) hebetispina limna** (*PML2A-e2*)*	JQ967322	6.6	**2**	**0.75**	/	/	/	/	/	/
***P. (P.) uniformis uniformis*** (WA28KT)	JQ967317	11.6	**1.5 – 2**	**0.5**	4.5	1.8	2.6	y	n	Small oblique blunt
*P.* (*P*.) aff. *uniformis uniformis* (WA32KAG)	JQ967319	8.1	**/**	**/**	/	/	/	/	/	/
***P. (P.) uniformis hemiradia*** (AU30KV)	JQ967318	9.9	**2**	**0.5 – 0.75**	4.6	1.8	2.6	y	n	Small oblique blunt
***P. (P.) cambrispina*** (WI34KN)	JQ967316	9.0	**2.0**	**0.5 – 0.75**	2.7	1.2	2.3	y	n	Small oblique blunt
***P. (P.) vulgaris vulgaris*** (PML2B)	JQ967314	9.0	**1.5 – 2**	**0.5**	3.9	2.1	2.1	y	n	Small oblique blunt
*P.* (*P*.) aff. *vulgaris vulgaris* (W03)	JQ967313	11.3	**2**	**0.5**	/	/	/	/	/	/
*P.* (*P*.) aff. *vulgaris vulgaris* (SW02)	JQ967315	/	**/**	**/**	3.3	1.8	1.8	y	n	Small oblique blunt
***P. (P.) vulgaris brevispina*** (PML4B)	JQ967311	10.4	**1.5 – 2**	**0.5**	2.4	1.9	1.2	y	n	Small oblique blunt
*P.* (*P*.) *vulgaris vulgaris* (PML8)		8.8	**1.5 – 2**	**0.5**	3.6	1.9	1.9	y	n	Small oblique blunt
***P. (P.) caroni*** (CH2)	JQ967292	7.1	**?**	**?**	1.9	1.0	1.9	y	n	Small oblique blunt
***P. (P.) petronia*** (J1)	GU220392	/	**/**	**/**	2.9	1.8	1.6	y	n	Small oblique blunt
***P. (P.) variosa*** (Ind5)	JQ967296	8.3	**1.5**	**0.5**	2.9	1.2	2.5	y	n	Tapered – blunt
***P. (P.) mantoni*** (BZ5a)	JQ967295	9.7	**1 – 4**	**0.5 – 1.0**	3.5	1.3	2.5	y	n	Tapered and oblique
*P.* (*P*.) aff. *mantoni* (Bassen)	JQ967294	10.1	**1.5 – 2**	**0.5 – 0.75**	/	/	/	/	/	/
***P. (P.) solis solis*** (GMCCL6)	JQ967309	8.3	**1.5 – 2**	**0.75**	2.6	1.1	2.6	y	n	Tapered – blunt
***P. (P.) solis crocotilla*** (UPL1B)	JQ967308	8.0	**1.5**	**0.5**	3.7	1.2	3.1	y	n	Tapered – blunt
***P. (P.) dimorpha*** (CA01)	JQ967310	6.7	**1.5 – 2**	**0.5 – 0.75**	3.2	1.1	3.0	y	n	Tapered – blunt
***P. (P.) longispina*** (MEX3)	JQ967305	8.8	**2 – 2.5**	**0.5**	5.7	1.4	4	y	n	Tapered – blunt
***P. (P.) stylata limnetica*** (PML5D)	JQ967306	9.4	**2 – 2.5**	**0.75**	5.4	1.3	4.0	Y	n	Tapered to fine tip?
***P. (P.) stylata stylata*** (W02)	JQ967307	6.9	**2.5**	**0.75 – 1**	3.6	1.3	2.8	y	n	Tapered – pinched
***P. (P.) sinensis*** (CH9)	JQ967303	9.8	**2.0**	**0.5 – 0.75**	2.6	1.3	2.1	y	n	Tapered with dull tip
***P. (P.) spiculosa edaphica*** (CH6)	JQ967302	9.1	**1.5**	**0.5 – 0.75**	/	/	/	/	/	/
***P. (P.) spiculosa terricola*** (GMBGL1)	JQ967301	9.7	**1.5 – 2**	**0.5**	3.2	1.0	3.1	y	n	Rounded
***P. (P.) spiculosa spiculosa*** (BZ8)	JQ967298	8.4	**2.0**	**0.5**	3.2	1.3	2.8	y	n	Rounded
*C. butcheri* (MD03)	JQ967291	3.3	**1.5 – 2**	**0.5 – 0.75**	n/a	n/a	n/a	n/a	n/a	n/a

Species are grouped in clades as seen in the molecular analyses. All numbers are means for each strain. Light microscope measurements are for live cells. As many measurements as possible were made for each criterion and averaged. These data show definite patterns corresponding to particular clades, especially, Long Cilium, Annulus and Dense Rim. *Paraphysomonas* subgenera are indicated by the letter in brackets in species name: (*Brevispina/Acrospina*/*Hebetomonas*/*Paraphysomonas*).

We cannot precisely compare new species with the type species *P. vestita* because its scale type is unknown. As the discussion explains more fully, cultures previously identified as ‘*P. vestita*’ have been repeatedly studied ultrastructurally since Houwink (1952) and Manton and Leedale (1961), but their scale structure differs as greatly as many species described here that have radically different sequences, so we cannot know which if any are really the same species as Stokes’ *P. vestita*. Either no data were given to enable identification to be checked (e.g. Houwink 1952) or those given strongly suggest that the organism studied was not *P. vestita* but an undescribed species (e.g. Manton and Leedale 1961). Ideally we would have liked to establish a neotype to end that confusion, but no isolate was sufficiently similar (by light microscopy) to Stokes’ (see discussion). Therefore it is unlikely that any new species described here for which we give LM data can be *vestita*. We formally raise subspecies *Paraphysomonas vestita truncata* (Preisig and Hibberd 1982a) to a full species, as its spine scales are distinct enough from other electron microscopically studied strains to merit that, its spines being too short for *P. vestita*: ***Paraphysomonas truncata*** Preisig and Hibberd, 1982a stat. n.; their diagnosis and type applies.

For brevity, many additional comments and information on most of the following 22 new species, including descriptions of separate isolates related to the type strains detailed below, are given only in Supplementary Information 1. Many studied strains are described only in the Supplementary material as aff. plus a specific epithet to indicate their likely closest relative, even though a few of them are shown in the figures or Table 2; some environmental sequences are also similarly identified there.

### Subgenus *Brevispina*: two new species

***Paraphysomonas ovalis*** sp. n. Type Fig. 3A – F. **Diagnosis:** CL 5.2 μm (4.1 – 6.4 *N* = 17); LC 1.5 – 2 × CL; SC 0.75 × CL. LC beats constantly. Attached cells round, sometimes flattened on one side. Swimming cells elongate to pyriform, sometimes round, swim in slow spiral and direct trajectories. Stalked cell close to substratum or attached to detritus. One type of spine scale with oval to irregular base plate. Spine 1.5 μm (1.1 – 1.9) tapers gently to rounded tip, slightly flared out at very base; base-plate 0.8 μm (0.7 – 0.95) with prominent dense margin. S/P ratio 1.9 (range 1.4 – 2.4). Type strain **ARB**: CCAP 935/15. (2010; Harcourt Arboretum, Oxfordshire, UK. JMS). Soil. Type 18S rDNA sequence GenBank JQ967331. **Etymology:** *ovalis* L. oval. **Comment:** *P. ovalis* is most similar to *P. bandaiensis*, *truncata*, and *porosa*; all have a base-plate with thickened margin. *P. ovalis* differs from them all by its base-plate being oval to irregular, not regularly circular; it is unperforated, unlike *P. porosa*. *P. ovalis* has a rounded spine tip; that of *P. truncata* is truncated. *P. bandaiensis* spine tip is also rounded but its shaft is non-tapered, unlike *P. ovalis*.

***Paraphysomonas segmenta*** sp. n. Type Fig. 3G – I. **Diagnosis:** CL 6.0 μm (5 – 7.3 *N* = 22); LC 2 × CL; SC 0.5 × CL. LC sometimes static. Round to oval cell attached via short stalk to substratum or detritus. Swimming cell oval to pyriform, stalk often trailing. One type of spine scale, spine 0.65 μm (0.52 – 0.73) usually in two non-tapering segments, distal half thinner than proximal half, ending in a rounded tip, sometimes more prominently flared at base than in *ovalis*. Round to oval base-plate 0.44 μm (0.40 – 0.53) with prominently thickened rim. S/P ratio 1.4 (1.3 – 1.7). Type strain: **KU3b2**. (Keele University, Staffordshire, UK. JMS). Fresh, pond water. 18S rDNA differs from *ovalis* by 31 nucleotide substitutions and a single-nucleotide indel: type sequence GenBank JQ967330. **Etymol:** *segmenta* L. segmented. **Comment:** *P. segmenta* is most similar to *P. bandaiensis*, also with very small scales, but with a thicker base-plate dense rim; *P. segmenta* spines lack the lateral striation of *P. truncata*. *P. bandaiensis* non-tapered spines have rounded tips, but its scales are much smaller; the spine is nearly a third shorter than in *P. segmenta*, base-plate diameter nearly half (Takahashi 1976).

### Subgenus *Acrospina*: four new species and one subspecies

***Paraphysomonas acuminata acuminata*** sp. n. Type Fig. 4A, B. **Diagnosis:** CL 9.0 μm (6.4 – 10 *N* = 22); LC 2.5 – 3 × CL; SC 0.75 – 1 × CL. Round bright cell commonly attached to substratum/detritus. Swimming cells common and slow. LC long, moves constantly; but often slows greatly, then undulating asymmetrically. Scales and spines conspicuous in LM. One form of spine scale covers cell. Spine 5.2 μm (4.2 – 6.7), non-tapering to barely tapering ending in strongly oblique short pointed tip. Spine base width 0.191 μm (0.132 – 0.228), spine tip width 0.133 (0.094 – 0.158). Oval to rounded and irregular-shaped base-plate 1.9 μm (1.5 – 2.1), no/barely visible dense margin, commonly with broad medium density annulus (i.e. a denser ring on the base-plate centred on the spine base) midway on base-plate. S/P ratio 2.8 (2.4 – 3.2). Type strain **PML6A** CCAP 935/18. (Port Meadow, Oxford, UK. JMS). Freshwater lake. 18S rDNA sequence GenBank JQ967329. **Etymol.** *Acumen* L. point.

***Paraphysomonas acuminata cuspidata*** subsp. n. Type Fig. 4F, G. **Diagnosis:** CL 9.2 μm (8.2 – 10.5 *N* = 16); LC 2.5 – 3 × CL; SC 0.5 – 0.75 × CL. Long LC, undulates often asymmetrically fast to gentle sometimes almost to a stop. Round to oval cells with spines obvious in LM especially at high magnification. Slow swimming stage common, commonly foraging at substratum. Swimming cell often pyriform with trailing stalk. One form of spine scale 4.7 μm (4.2 – 5.0), barely tapering spine to a short oblique point starting from spine-shaft, base width 0.17 μm (0.15 – 0.20), tip width 0.089 μm (0.07 – 0.10). Spine positioned centrally from oval to irregular base-plate 1.6 μm (1.2 – 2.1) no dense margin, common mid-point annulus. S/P ratio 3.0 (2.2 – 3.4). Type strain **PR26KB**. (Freshwater, Austria. JB). 18S rDNA differs from nominal subspecies by two nucleotide substitutions and a single nucleotide deletion: type sequence GenBank JQ967326. **Etymol.** *cuspis* L. pointed. **Comment:** *P*. *acuminata cuspidata* spine tips may be blunter than *P. acuminata acuminata*, and *P. a. cuspidata* cells and scales are somewhat smaller than *P. a. acuminata*.

***Paraphysomonas mikadiforma*** sp. n. Type Fig. 4H – I. **Diagnosis:** CL 7.0 μm (6.4 – 8.2 *N* = 20); LC 3.5 – 5.0 × CL; SC 1 × CL. Stalked cell very round. LC can be very long, appearing like a dark hair; asymmetric undulation, fast to slow. Trailing stalk with detritus common. Swimming cell common, often elongate or pyriform. One form of spine scale, visible in LM. Spine 5.2 μm (3.8 – 5.9), non-tapering to barely tapering with small oblique pointed tip. Spine base width 0.13 μm (0.1 – 0.16), tip width 0.094 μm (0.053 – 0.123). Base-plate 2.1 μm (2 – 2.3) irregular oval to round, common midpoint annulus, no dense margin. S/P ratio 2.5 (1.8 – 3.0). Type strain **JBM02.** (Lake Mondsee, Austria. JB). Freshwater. 18S rDNA sequence has 10 nucleotide substitutions compared with *P. acuminata acuminata*, six differences from *lucasi*, but 14 substitutions and a single nucleotide indel compared with *perforata*. GenBank JQ967325. **Etymol.** *mikado* popular generic Japanese game of pick-up-sticks; spine resembles sticks. **Comment:** *P*. *mikadiforma* cells are notably smaller than its close freshwater relatives on the tree, *P*. *a*. *acuminata*, as well as the exceptionally longer LC, this difference associated with substantial molecular divergence.

***Paraphysomonas lucasi*** sp. n. Type Fig. 5A, B. **Diagnosis**: CL 3.6 μm (3.2 – 5.0 *N* = 25); LC 2.5 – 3 × CL; SC 0.75 × CL. Small bright round to oval or irregular cell, commonly attached to substratum via short stalk; evenly spaced cells. Fast swimming common. LC often static, held in a curved kinked position. One type of spine scale. Spine 0.92 μm (0.8 – 1.2), barely tapers to slight shoulder (not always visible) usually somewhat below half-way up spine (variable), continues to barely taper until small oblique rounded tip. Spine base width; 0.042 μm (0.035 – 0.049), spine tip width; 0.017 μm (0.011 – 0.23). Spine protrudes centrally from round to oval base-plate 0.7 μm (0.64 – 0.77); no dense margin or annulus. S/P ratio 1.3 (1.1 – 1.9). Type strain **NC10-16**. (Wrightsville Beach salt marsh, North Carolina, USA. JMS). Marine. 18S rDNA sequence GenBank JQ967323. **Etymol.** *lucasi*, after I. A. N. Lucas for contributions to *Paraphysomonas* research. **Comment**: *P. lucasi* is similar to *P. imperforata* (Lucas 1967), but despite both being marine *P. lucasi* is smaller on average and cilia lengths differ slightly. The scales of *P. lucasi* have marginally smaller base-plates and no annulus; *P. imperforata* always exhibits an annulus.

***Paraphysomonas perforata*** sp. n. Type illustration Fig. 1C of Rice et al. (1997). **Diagnosis:** One type of spine scale with perforated base-plate (0.73 μm) with uninterrupted disordered perforation over the entire base except for a small unperforated non-dense margin. Central, non-tapering spine with rounded tip (1.1 μm). Original strain isolated by S. Tong from Southampton Water, U.K. (Rice et al. 1997). 18S rDNA differes from the closest imperforate species *lucasi* by nine substitutions and an indel. Type sequence GenBank Z38025; SOTON A. **Etymol.** Perforated base-plate. **Comment**: The original *P. foraminifera* Lucas (1967) spine scale was larger on average than in *P. perforata*, which does not even reach the lower ranges of *P. foraminifera* scale measurements (spine 1.46 – 1.63 μm; base-plate 0.97 – 1.12 μm).

### Subgenus *Hebetomonas*: three new species and one new subspecies

***Paraphysomonas hebes*** sp. n. Type Fig. 6A – E. **Diagnosis**: CL 4.7 μm (3.2 – 5.9 *N* = 37); LC 2.5 – 3 × CL; SC 0.5 – 1 × CL. LC beats constantly at various speeds. Small cell round, oval sometimes appearing irregular, stalked close to substratum, often swimming with trailing stalk and detritus. Often congregates with other cells. Scale-base layer just visible using X60 LM objective. One type of spine scale, spine 1.4 μm (1.3 – 5.6) varies in length and barely tapers to truncate tip; basal width of spine 0.034 μm (0.028 – 0.043), width of spine tip 0.018 μm (0.012 – 0.024). Spine often curved or bent, smooth, no bulges or inflation. Round to oval base plate 0.6 μm (0.5 – 0.7), barely noticeably denser margin; no annulus. S/P ratio 2.4 (2.0 – 2.8). 18S rDNA sequence has 15 substitutions and two deletions compared with *hebetispina*. Type seunce GenBank JQ967320. Type strain **Ind1:** CCAP 935/17 (India, Goan sandy beach; coll TCS, isol. JMS). Marine surf. **Etymol.** *hebes* L. blunt: **Comment:** *P. hebes* differs from all former *Paraphysomonas* species with its slightly tapering spine and truncate tip.

***Paraphysomonas hebetispina hebetispina*** sp. n. Type Fig. 6G – M. **Diagnosis**: CL 5.3 μm (3.2 – 5.9 *N* = 37); LC 2× CL; SC 0.5 × CL. Small oval to round cells with undulating LC moving fast to static, often twitching whole cell with fast ciliary movement. Sometimes seen in water column in groups joined together via short stalk and detritus. No scale evidence in LM except bacteria being stuck in position away from cell and faint scale-base layer halo. One form of spine scale; spine 1.2 μm (0.9 – 2.2) slender, barely tapers to blunt or slightly rounded tip. Spine base width 0.032 μm (0.023 – 0.037), spine tip width 0.02 μm (0.015 – 0.028). Base-plate width 0.5 μm (0.39 – 0.61), faint concentric annulus in some scales only; slightly denser rim. S/P ratio 2.5 (1.6 – 3.7). Type strain **NC10-20**. (Drainage ditch, North Carolina, Cape Fear River, Wilmington. JMS). Brackish – treated as marine. **Etymol.** *hebes* L. blunt: 18S rDNA sequence GenBank JQ967321. **Comment:** Scale spine tips vary from rounded to flatly truncate; some spines appear slightly thicker than others. Differs from *P. hebes* by 18S rDNA and presence of scale base-plate annulus and tip variation.

***Paraphysomonas hebetispina limna*** subsp. n. Type Fig. 6F. **Diagnosis**: CL 6.6 μm (6.4 – 6.8 *N* = 7); LC 2 × CL; SC 0.75 × CL. Small round to irregular-shaped cells, most on substratum. Occasional jerky movement from strong LC beat. Scale presence not obvious in LM except occasional faint scale-base layer. Type 18S rDNA sequence GenBank JQ967322 differs from *P. h. hebetispina* by five substitutions. Strain **PML2A-e2** (Port Meadow, Oxford, UK. JMS). Freshwater still stream. **Etymol.** *limna* Gk Lake. **Comments.** The culture died before it could be observed in the electron microscope; distinct from *P. hebes* and *P. hebetespina hebetespina* in being from fresh water, and might deserve species rank if scales clearly differ.

***Paraphysomonas parahebes*** sp. n. Type figure: Fig. 1A, B of Caron et al. (1999). **Diagnosis**: One type of spine scale; spine 1 μm, barely tapering to rounded or blunt tip, base-plate with dense margin, 0.6 μm. (strain not stated from which of two; HFlag, WH1). Type sequence GenBank AF109322. **Etymol**: *para* Gk. beside *hebes* L. blunt. **Comment**. This cell, misidentified as *P. bandaiensis* (Caron et al. 1999), had very different scales from the original *P. bandaiensis* (Takahashi 1976), which had an extremely dense base-plate margin and spine 0.3 μm and base-plate diameter only 0.3 μm. The spine scale in Caron et al. (1999) is over twice as large and its base plate dense margin is much less thick, as in the *Hebetomonas* clade to which it is sister.

### Subgenus *Paraphysomonas*, clades A – E: 13 new species and six subspecies

**Clade A**, the most divergent short-branch clade of subgenus *Paraphysomonas*, is exclusively freshwater and comprises *Paraphysomonas uniformis* and its subspecies, plus several morphologically uncharacterised lineages:

***Paraphysomonas uniformis uniformis*** sp. n. Type Fig. 7F, G. **Diagnosis.** CL 11.6 μm (6.8 – 16.4 *N* = 31); LC × 1.5 – 2 CL, SC × 0.5 CL. SC difficult to see in LM. Extremely plastic cells, round to oval and pyriform when swimming, often with trailing stalk and attached detritus. Mostly attached to substratum, swimmers common. Scales visible in LM: obvious scale-base layer, spines less clear. One type of spine scale, spine 4.5 μm (4 – 5.3), often gently tapered crooked/straight spine with small oblique blunt point. Spine base width 0.116 μm (0.088 – 0.153), spine tip width 0.054 μm (0.042 – 0.071). Round to oval base-plate 1.8 μm (1.5 – 1.9) with thin dense margin. S/P ratio 2.6 (2.2 – 2.9). Base of spine sometimes inflated; has transverse crease across centre. Type strain **WA28KT (**Wallersee, Austria. JB Freshwater lake.). Type sequence GenBank JQ967317. **Etymol.** *uniformis* L. uniform.

***Paraphysomonas uniformis hemiradia*** sp. n. Type Fig. 7A – E. **Diagnosis:** CL 9.9 μm (7.3 – 14.1 *N* = 27); LC 2 × CL; SC 0.5 – 0.75 × CL. Bright round to oval cell with obvious halo, spines sometimes visible in LM. Plastic, usually stalked cell. Swimming cell oval, elongate to pyriform often with trailing stalk and detritus. One type of spine scale; spine often bent; 4.6 μm (3.6 – 5.8) varying length and thickness, commonly has broad base 0.2 μm (0.07 – 0.3) and basal inflation/bulge, tapering to small oblique dull point, 0.046 μm (0.028 – 0.063). Round to oval base-plate 1.8 μm (1.5 – 2), very dense rim. S/P ratio 2.6 (2 – 2.9). Distinguished from *P. u. uniformis* by base of spine often being unusually broad, flaring onto plate, and by most base plates having about 8 prominent radial creases (Fig. 7B – D); some smaller scales (Fig. 7E) lack the creases. Type strain **AU30KV** (Lake Augstsee, Austria. JB). Freshwater lake. Type 18S rDNA partial sequence 9931) GenBank JQ967318, has only one substition compared with ssp. *uniformis*, which is in a rather conserved position so might be a sequencing error, so they might really be identical. **Etymol:** *hemi* Gk. half, *radius* L. radial. **Comment:** No other named species has such prominent radial ‘spokes‘, but they are evident in unnamed micrographs (Figures 11, 12 of Řezáčová and Škaloud 2004); whether they are artefactual creasing during TEM preparation or a permanent structure is unclear, but in either case its reproducibility must reflect a basic difference from *P*. *u. uniformis*, possibly in base-plate plasticity.

**Clade B**, also exclusively freshwater, comprises the next two species (plus morphologically similar or uncharacterised lineages closely related to *P. vulgaris*) with oblique spine tips, forming a longer-branch on Fig. 1.

***Paraphysomonas cambrispina*** sp. n. Type Fig. 8G, H. **Diagnosis:** CL 9.0 μm (7.3 – 11.4 *N* = 20); LC × 2 CL; SC × 0.5 – 0.75 CL, obvious in LM. Plastic oval to round cells. Pyriform when swimming, slowly, with stalk often trailing behind. Obvious scale-base layer in LM. One form of spine scale; spine 2.7 μm (2.0 – 3.1), tapering, sometimes curved, to oblique dull tip. Base-plate 1.2 μm (0.97 – 1.67) with dense rim. S/P ratio 2.3 (1.8 – 2.7). Type strain **WI34KN**. (Austria. JB). Freshwater lake. 18S rDNA sequence GenBank JQ967316 has 26 differences from *P. vulgaris*. **Etymol.** *camber* L. curved. **Comment.** Unlike the others in Clade B, *P. cambrispina* has no obvious transverse crease on the base plate.

***Paraphysomonas vulgaris vulgaris*** sp. and ssp. n. Type Fig. 8C, D. **Diagnosis.** CL 9.0 μm (7.7 – 12.7 *N* = 26); LC × 1.5 – 2 CL, SC × 0.5 – 0.75 CL. Round to oval, bright plastic cells; conspicuous scale-base layer in LM, sometimes spines visible. Commonly attached to detritus or substratum, often swimming with trailing stalk and detritus. Cell swims smoothly through water column. One type of spine scale; spine 3.9 μm (3.1 – 4.5), gently tapering from wide, usually bulbous, base to oblique dull pointed tip. Spine base width 0.2 μm (0.014 – 0.31), spine tip width 0.063 μm (0.052 – 0.068). Base-plate 2.1 μm (1.8 – 2.2), oval to round with dense rim; transverse crease across centre. S/P ratio 1.8 (1.6 – 2.0). Type strain **PML2B** (Non-flowing stream, Port Meadow, Oxford, UK. JMS). Freshwater. Type 18S rDNA sequence GenBank JQ967314. **Etymol.** *vulgaris* L. common, because 10 independent strains (including *P. v. brevispina*) had identical 18S rDNA, showing that this species is common and widespread in temperate and tropical habitats across the old world (details in Supplementary Material).

***Paraphysomonas vulgaris brevispina*** subsp. n. Type Fig. 9A, B. **Diagnosis:** CL 10.4 μm diameter (8.2 – 12.3 *N* = 24); LC 1.5 – 2 × CL; SC 0.5 × CL. Round to oval cells, often stalked to substratum/detritus. Swimming cell often elongate/pyriform. Scale-base layer visible in LM, spines inconspicuous. Plastic cell, especially when ingesting bacterial aggregates. One type of spine scale; spine 2.4 μm (1.8 – 3.1), commonly inflated at base ∼0.35 μm wide, above which spine sometimes crooked or bent, gently tapering to oblique dull point. Oval to round/irregular base-plate, obvious dense rim, base-plate 1.9 μm (1.7 – 2.2), S/P ratio 1.2 (1 – 1.6). Small base-plate crease beside spine base frequent. Type strain **PML4B** (Wetland temporary pond, Port Meadow, Oxfordshire, UK. JMS). Freshwater. 18S rDNA sequence GenBank JQ967311 identical to *P. vulgaris vulgaris*. **Etymol.** *brevispina* L. short spines, referring to its notably shorter spines than *P*. *v. vulgaris*.

**Clade C** is the second major long-branch freshwater clade in Fig. 1, comprising the next four species and less well characterised lineages described in Supplementary Material. All four are clearly different in rDNA and also distinguishable by scale dimensions and spine tip detail:

***Paraphysomonas variosa*** sp. n. Type Fig. 10A – F. **Diagnosis**: CL 8.3 μm (7.3 – 9.1 *N* = 14); LC 1.5 × CL; SC 0.5 × CL. LC constant motion, although slows to an asymmetrical undulation. Difficult to see LC and SC on stationary cells in LM because of orientation of cell. Round to oval cell, commonly attached to substratum or detritus. Swimming cells usually oval to elongate, swimming high in water column. One type of spine scale; spine 2.9 μm (1.4 – 3.7), tapering to blunt tip. Base width of spine 0.14 μm (0.08 – 0.24); tip width 0.027 μm (0.015 – 0.049). Round to oval base plate 1.2 μm (1 – 1.5); S/P ratio 2.5 (1.6 – 3.7), conspicuous to barely visible inflection at rim. Type strain **Ind5** (Freshwater, India. Coll. TCS. JMS). 18S rDNA sequence, GenBank JQ967296. **Etymol:** *variosa* L. various. **Comment:** *P. variosa*'s spine tip is most similar to *P. stylata stylata* in that some have a more prominent pinched tip.

***Paraphysomonas caroni*** sp. n. Type Fig. 10G – K. **Diagnosis**: CL 7.3 μm (5.9 – 9.6 *N* = 48); LC1.5 – 2 × CL; SC 0.5 × CL. Round to oval cell with scale-base layer sometimes visible on larger cells. Often trailing stalk when swimming. One type of spined scale; spine 1.9 μm (1.3 – 2.1) tapering to short oblique blunt pointed tip. Round base-plate with obvious dense margin 1.0 μm (0.9 – 1.1); S/P ratio 1.9 (1.4 – 2.3). Type strain **CH2**. (Marsh in zoo, Beijing, China. Coll TC-S. JMS). Freshwater. 18S rDNA sequence, GenBank JQ967292. **Etymol:** named after D. A. Caron for his contribution to *Paraphysomonas* research.

***Paraphysomonas mantoni*** sp. n. Type Fig. 10M – L. **Diagnosis:** CL 6.8 – 20.5 μm; modal and median value, 8.6. Average, 9.7 μm: *N* = 31. LC, 1 – 4 ×CL smaller cells than ∼8.6 μm appeared to have longer LC from 2.5 – 4 × CL. Spines 3.5 μm (2.2 – 5.2) taper to a dull point (Fig. 10 M) or else to a small oblique tip (Fig. 10 N). Oval base-plate 1.3 μm (1 – 1.6) with dense rim. S/P 2.5 (1.9 – 3.6). Cells with longer cilia seem to have more prominent spines in LM as well as obvious scale-base layer. In very plastic large cells with large vacuoles, scale presence not obvious. Type strain **BZ5a** (Freshwater, Brazil. JMS). Type 18S rDNA sequence GenBank JQ967295. **Etymol:** named after I. Manton for her contribution to *Paraphysomonas* research.

***Paraphysomonas petronia*** sp. n. Type illustration Fig. 2A of Petronio and Rivera (2010). **Diagnosis**: One type of spine scale covers cell; spine 2.9 μm (2.5 – 3.0), tapering to small oblique dull tip, centrally protruding from round to oval base plate 1.8 μm (1.6 – 2.0), with dense margin and no perforations. Type strain **J1** (Laguna de Bay, Philippines) (Petronio and Rivera 2010). Freshwater. Type 18S rDNA sequence: GenBank GU220392. **Etymol:** named after first author, JAG Petronio. **Comment:** Its unique large 137 nt insert (position 938 – 1075) in 18S rDNA was not seen in any other *Paraphysomonas*; when blasted against GenBank a match was only made to *Paraphysomonas*. The insert matches closely, not exactly, an adjacent part of its sequence, so is a fairly recent duplication.

**Clade D**. The next four long-branch species with relatively long spines and each with very distinct rDNA have much more varied habitat than other clades: soil, freshwater, and marine:

***Paraphysomonas solis solis*** sp. n. Type Fig. 12A, B. **Diagnosis:** CL 8.3 μm (6.4 – 11.4 *N* = 32); LC 1.5 – 2 × CL; SC 7.5 × CL. Oval to round cells; often oval when swimming. One type of spine scale; spine 2.6 μm (1.5 – 4.4) tapers strongly to dull point, base-plate 1.1 μm (0.8 – 1.3) oval to round with a varying dense rim. Type strain **GMCCL6** (Wet mud from end of stream, Christ Church Parks, Oxford, UK. JMS). Freshwater. 18S rDNA sequence GenBank JQ967309 has 19 nucleotide differences from *P. solis crocotilla*. **Etymol:** *solum* L. soil.

***Paraphysomonas solis crocotilla*** subsp. n. Type Fig. 11A – F. **Diagnosis:** CL 8 μm (6.8 – 9.1 *N* = 20) LC 1.5 × CL; SC 0.5 × CL. Round to oval and elongate cells, swim fast up and down water column. One form of spine scale; spine 3.7 μm (2.8 – 4.6) slender, sometimes slightly curved, gently tapers to a blunt tip. Spine base usually slightly inflated, width 0.13 μm (0.103 – 0.188), spine tip width 0.017 μm (0.011 – 0.021), larger inflation at the base rare (0.3 μm). Base-plate 1.2 μm (1.0 – 1.6 μm) with delicate but conspicuous dense rim. S/P ratio 3.1 (2.5 – 3.8). Type strain **UPL1B** (Soil from mole-hill under tree, University Parks, Oxford, UK. JMS). 18S rDNA sequence, GenBank JQ967308. **Etymol:** *crocotilla* L. slim. **Comment:** Some early micrographs suggest that it sometimes also has spineless scales; one round to oval measured 1.4 μm (Fig. 11B). Spineless scales were not observed when TEM was repeated.

***Paraphysomonas dimorpha*** sp. n. Type Fig. 11 H – M. **Diagnosis:** CL 6.7 μm (5 – 8.6 *N* = 22); LC 1.5 – 2 × CL; SC 0.5 – 7.5 × CL. LC moves constantly at varying rates, sometime slowing almost to a stop, exhibiting slow undulation. Cell round to oval, sometimes pyriform when swimming; thick scale-base layer conspicuous in LM. Stalked cell close to substratum/detritus. Two forms of scale; spine scales with round base-plate and dense rim; spine 3.2 μm (2.1 – 4.6) tapers gently slightly curved to a blunt end and often starts from base with slight bulge; base-plate 1.1 μm (1.0 – 1.2 μm), S/P ratio 3.0 (2.0 – 4.2). Spineless scales round to slightly oval, 1.3 (1.1 – 1.6 μm), sometimes have central short line (120 nm). Cell sometimes covered in one scale type but often covered in both forms. Type strain **CA01** CCAP 935/16. (Leaves, grass and mud, Monterey Bay, California, USA. JMS). Freshwater. 18S rDNA sequence, GenBank JQ967310. **Etymol:** *dimorpha* L. two forms, signifies two scale types.

***Paraphysomonas longispina*** sp. n. Type Fig. 13A, B. **Diagnosis:** CL 6.9 μm (6.8 – 13.6 *N* = 46); LC 2 – 2.5 × CL; SC 0.75 – 0.5 × CL. Plastic cells with large vacuoles, oval to round. Readily ingests cells of own kind. Scale-base layer visible in LM. One form of spine scale; spine 5.7 μm (2.2 – 7.3) tapering to a dull point, often slightly curved along whole length. Base-plate 1.4 μm (0.9 – 1.7), round to oval with prominent delicate inflexed rim. S/P ratio 4.0 (2 – 5.3). Type strain **MEX3** (Calzadas River, Calzadas Coatzacoalcos, Veracruz, Mexico. JMS). Freshwater. 18S rDNA sequence, GenBank JQ967305. **Etymol.** *longus* L. long, *spina* L. backbone/spine.

***Paraphysomonas stylata stylata*** sp. n. Type Fig. 12C – F. **Diagnosis:** CL 6.9 μm (5.5 – 8.2 *N* = 21); LC 2.5 × CL; SC 0.5 – 0.75 × CL. LC beats constantly at various speeds. Bright round to oval cell. Commonly attached to substratum and floating debris by stalk of various lengths. Swimming cells round to oval and elongate, sometimes pyriform with stalk trailing. Scale-base layer visible in LM. One type of scale; spine 3.6 μm (2.4 – 5.5) tapering to pinched tip. Spine base width, 0.079 μm (0.06 – 0.14). Round to oval base-plate 1.3 μm (1.0 – 1.6) with dense rim. Transverse central crease on base-plate often seen. S/P ratio 2.8 (1.7 – 3.7). Type strain: **W02.** (Wet beach sand, Pembrokeshire, UK. JMS). Marine. Type 18S rDNA sequence GenBank JQ967307. **Etymol.** *stylus* Gk writing instrument. **Comment:** The C at position 535 in *P. s. stylata* 18S rDNA is a T in all other chrysomonads and so is probably a PCR or sequencing error in this strain. If not studied carefully, the *P. s. stylata* spine tip could be confused with the oblique tips of clade B, but the blunt end of *P. s. stylata* is broader when unpinched, exhibiting little change or interruption in overall spine tapering. *P. s. stylata* is most similar to freshwater *P. stylata limnetica*, its sister sequence on Fig. 1, with much longer spines:

***Paraphysomonas stylata limnetica*** subsp. n. Type Fig. 13E, F. **Diagnosis:** CL 9.4 μm (7.7 – 12.3 *N* = 14); LC 2.0 – 2.5 × CL; SC 0.75 × CL. Plastic oval to round cell. Scale-base layer and nucleus conspicuous in LM. Swimming cells elongated and oval. One form of spine scale; spine 5.4 μm (3.8 – 7.2), gently tapering completely to a tip, spine base width 0.082 μm (0.06 – 0.12). Oval to round base-plate with delicate thickened inflexed margin, 1.3 μm (0.9 – 1.7). S/P ratio 4.0 (3 – 6.8). Type strain: **PML5D** (from water flooded over grassy riverbank, Port Meadow, Oxford, UK. JMS). 18S rDNA sequence GenBank JQ967306 differs from *P. stylata stylata* by two nucleotides (3 if position 535 of *P. s. stylata* is genuinely a C). **Etymol:** *stylus* Gk writing instrument. **Comment:** tapering is stronger and comes to a more slender end in *P. s. limnetica* than in marine *P. s. stylata*, which commonly comes to a pinched tip; their spine base widths match closely.

**Fig. 16:**
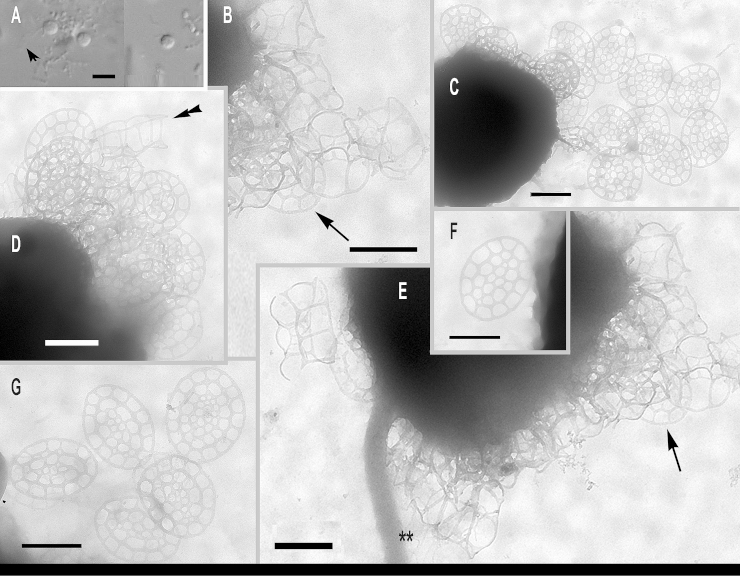
Micrographs of ***Clathromonas butcheri*** strain MD03. **(A)** DIC of live *C. butcheri* cells, beat envelope visible (arrowhead). **(B – G)** TEMs. **(B)** Scales, mostly crown scales but one plate scale (arrow). **(C)** Cell with only plate scales. **(D)** Cell with mostly plate scales but one crown scale seen from the side (double arrowhead). **(E)** Cell with mostly crown scales and a plate scale (arrow), some mastigonemes visible (**). **(F, G)** Plate scales. Scale bar: A, 5 μm. B – G, 0.5 μm.

**Clade E**, exclusively from soil, comprises the next two species, distinctly different in rDNA, with a tendency to form shrunken smaller cells in culture. The subspecies of *P. spiculosa* form a very distinct longer branch subclade (Fig. 1) and readily encyst as collared stomatocysts of subspecies-specific morphology:

***Paraphysomonas sinensis*** sp. n. Type Fig. 14A – E. **Diagnosis:** CL 9.8 μm (7.7 – 13.6 *N* = 25); LC 2.0 × CL; SC 0.5 – 0.75 × CL. Round to oval large plastic cell. Base layer of scales just visible in LM. Short stalk, sometimes thicker nearer cell end. Scales heterogeneous, commonly spine scales with rounded base-plate and dense rim; base-plate width 1.3 μm (1.1 – 1.5); spine length 2.62 μm (1.8 – 3.4) S/P ratio 2.1 (1.5 – 2.7). Spineless larger plate scales present, sometime with a central stub or stump, usually much larger than base-plate of spined scales, 2.1 μm (1.5 – 2.7). Aberrant scale forms common; mostly spine is hyper-inflated and/or obscurely shaped. Large spineless scales commonly have marks/scarring in the centre. Strain: **CH9**. (Soil, Yunnan, China. JMS). 18S rDNA sequence GenBank JQ967303. **Etymol.** *sinensis* L. Chinese. **Comment:** Only pictures of the dormant-looking cells captured in the light microscope (Fig. 16A) before the culture died.

***Paraphysomonas spiculosa spiculosa*** sp. n. Type Fig. 15C – E. **Diagnosis:** CL 8.4 μm (6.4 – 12.3 *N* = 36); LC 2.0 × CL: SC 0.5 × CL. LC in constant motion, slows to symmetrical undulation. Round to oval cell, attached to substrate/detritus; swimming cells oval to elongate, trail stalk and detritus. Plugged stomatocysts common, width ∼5.2 μm, collar height ∼2.1 μm. One form of spine scale; spine 3.2 μm (2.4 – 3.6) tapers to subtly rounded tip, spine base width 0.11 μm (0.088 – 0.118), spine tip width 0.027 μm (0.022 – 0.029). Round to oval base-plate 1.3 μm (1 – 1.8) delicate conspicuous dense rim. S/P ratio 2.8 (1.33 – 3.6). Type strain **BZ8** CCAP 935/19 (Soil and leaf litter, Brazil. JMS). 18S rDNA sequence, GenBank JQ967298. **Etymol**. *spiculosa*, as Stokes (1885) called the radial projections of *P. vestita* spicules. **Comment:** Stomatocyst similar size to *P. spiculosa terricola* but collar much shorter, about half the height of *terricola*, and curved in side view (unlike *P*. *spiculosa edaphica*). Its closest sister on Fig. 1 is *P. s. edaphica*.

***Paraphysomonas spiculosa terricola*** subsp. n. Type Fig. 15A, B. **Diagnosis:** CL 9.7 μm (7.3 – 10.9 *N* = 11); LC 1.5 – 2.0 × CL: SC 0.5 × CL. Oval to round cells, sometimes elongate when swimming, often trail stalk. Scale-base layer, sometimes spines, visible in LM. Plugged stomatocysts common (6.8 – 10 μm); refractile collar around stoma tall (3.7 – 4.4 μm), almost as high as its basal width, slightly undulating (less straight than in *edaphica*) and with thicker margin. One scale form. Spine 3.2 μm (2.2 – 5.4) tapering to a subtle rounded tip from a sometimes inflated spine base, spine base width 0.083 μm (0.05 – 0.12), spine tip width 0.021 μm (0.02 – 0.03). Oval to round base-plate, 1.0 μm (0.8 – 1.3) with obvious, sometimes delicate dense rim. Type strain: **GMBGL1** (Soil, Botanic Gardens, Oxford, UK. JMS). Freshwater. Type 18S rDNA sequence GenBank JQ967301 differs from *P. spiculosa spiculosa* by 13 substitutions and three indels. **Etymol.** *terra* L. earth *cola* inhabit.

***Paraphysomonas spiculosa edaphica*** subsp. n. Type Fig. 15F. **Diagnosis:** CL 9.1 μm (9.1 – 9.1 *N* = 1); LC 1.5 × CL; SC 0.5 – 0.75 × CL. Round to oval cells. Scale-base layer visible in LM. Plugged stomatocysts (diameter ∼8 μm) common; collar around stoma straight sided, height ∼3.1 μm, 4.4 μm diameter at base. Swimming cell often elongated with trailing stalk and detritus. Type strain: **CH6** (Soil, Beijing, China. JMS). Type 18S rDNA sequence GenBank JQ967302 differs from *P. spiculosa spiculosa* by 3 substitutions and four indels. **Etymol:** *edaphos* Gk ground.

### Two new *Clathromonas* species

We designated *C. butcheri* the type of *Clathromonas* because it was the only species we were able to culture and thus obtain DNA sequences to define its phylogenetic position. Two sequences attributed previously to *C.* (=*Paraphysomonas*) *butcheri* (Rice et al., 1997, Caron et al., 1999) cannot be from that species as the scale micrographs included in the sequencing papers show distinct morphological differences from the original culture (Pennick and Clarke 1972) that died before DNA sequencing was invented. The two sequences also differ significantly from each other and from our strain, but all three are part of the same clathromonad subclade (Supplementary Fig. S3). As the scales of those two earlier strains also differ from all other described species we make them new species, but first describe our new isolate to demonstrate that it is indistinguishable from the original *P. butcheri.*

We isolated an arguably genuine ***Clathromonas butcheri*** (Strain MD03, CCAP 936/1) in 2010 from brackish waters in Chesapeake Bay, Queenstown, Maryland, USA. 18S sequence GenBank JQ967291 (Fig. 16). **Description:** CL 3.3 μm (2.7 – 6.3: *N* = 14); LC 1.5 – 2.0 × CL. SC 0.5 – 0.75 × CL. LC beats constantly. Round, often bright cell stalked close to substratum/detritus. Jerky movement common from LC temporarily changing/stopping movement. SC clearly seen by LM. Swimming stage common with trailing stalk, often high in water column, pyriform cell. Swimming slow, cell body rotating vaguely in situ with LC flailing outward. Two scale forms; mesh plate and basket. Plate scales 0.7 × 0.6 μm (0.52 – 0.87 × 0.40 – 0.72), bear 11 – 16 holes in the outer ring and 9 – 13 on the inner ring and a central area of irregularly placed holes the centre of the plate scale. Sometimes cell predominantly has more of one scale type or the other, sometimes equal amounts. **Comment:** The basket scales of our strain have the same structure and size (0.5 μm width) as the originals but we see more 6-strutted than 5-strutted basket scales, the reverse of the original. Even so, we consider MD03 an authentic live strain of *C. butcheri.* Its sequence is very different from the two other, supposedly ‘*butcheri*’, strains here made new species:

***Clathromonas tongi*** sp. n. **Diagnosis:** Two forms of scale; plate scale oval to round open mesh 0.55 μm (0.35 – 0.77 *N* = 4), with 5 – 9 outer holes of fairly regular size, not 11 – 16 as in *C. butcheri*. Internal holes are fewer than in *C. butcheri*: ∼2 – 6. Sizes of plate scales vary a lot. Basket scale 0.69 μm (0.71 – 0.67 *N* = 2), open mesh very angular shape, no curves 5 – 6 struts 0.28 μm (0.23 – 0.31). Type illustration: Fig. 1d in Rice et al. (1997); all measurements taken from illustration. Type 18S rDNA sequence GenBank Z29679. Original strain: **SOTONA** (Southampton Water, England, UK. Marine) no longer available. **Etymol.** named after its isolator S. M. Tong. **Comment:** It is morphologically distinct from *C. caroni* in scale dimensions, and outer plate scale hole numbers do not overlap with the range for *C. butcheri*; the basket scales are less angular. *C. tongi* has similar dimensions to *C. butcheri* but has plate scales more diverse in size with many fewer holes.

***Clathromonas caroni*** sp. n. **Diagnosis:** CL, at least 3.3 μm LC = ×2 CL (*N* = 1, Lim et al., 2001). Two scale forms: open mesh plate and basket scales; all descriptions based on Caron et al. (1999) and Lim et al. (2001). Plate scale, oval to round, length 0.92 μm (0.67 – 1.25); peripheral ring of holes (8 – 12) sometimes with smaller perforations at joins as well as irregular holes in centre. One plate scale with inner ring of ∼9 holes, but seven plate scales showed irregular size and shape of central holes. Basket scale (one) 0.9 μm wide, five struts with curved upper tier on oval to round ring, strut ∼ 0.16 μm. Unclear number of holes in upper tier, but appear irregular in shape and size, similar to holes in plate scales. Type illustrations; Fig. 1f from Caron et al. (1999) and Fig. 3a,b from Lim et al. (2001). Type strain: **DB4** (Patuxent River, Maryland, USA). Marine. Type 18S rDNA sequence GenBank AF109326. **Etymol.** *caroni* after D. A. Caron, author of both papers on this species. **Comment:** The single basket scale looks more like *C. inconspicua* (Takahashi, 1976), but plate scales are clearly different and sometimes resemble the top tier of the basket scale in *C. corbidifera* (Pennick and Clarke, 1973). The basket scale is larger than *C. butcheri* with shorter struts. The variety of hole-shapes and sizes is similar but scales are bigger than in *C. butcheri* and plate-scale holes fewer on average, though ranges overlap.

## Discussion

Our major conclusion is that *Paraphysomonas* was formerly a heterogeneous repository for all non-photosynthetic scaly chrysophytes irrespective of their vastly differing scale types and was far too diverse morphologically and genetically to be accepted as a single genus. The revised *Paraphysomonas* and *Clathromonas* are now relatively uniform in scale structure. As we specifically targeted heterotrophic cells for culturing it is unsurprising that we obtained representatives only of two purely heterotrophic scaly families, Paraphysomonadidae and Clathromonadidae, both in Paraphysomonadida. Former *Paraphysomonas* species with very different scales, now excluded from these morphologically homogenous and phylogenetically strongly supported genera, are being reassigned to new genera in a separate paper, and include some photosynthetic species unlikely to belong in Paraphysomonadida. Whether Clathromonadidae is sister (Fig. 1) or not (Fig. 2) to Paraphysomonadidae in 18S rDNA trees depends in part on taxon sampling; a recent tree omitting environmental sequences included here placed them consistently, sometimes strongly sisters by three methods (Škaloud et al. 2013). Support for Paraphysomonadida being holophyletic is generally higher on trees showing it as a clade (e.g. Škaloud et al. 2013) than the never significant support for it contradictorily being paraphyletic (e.g. Fig. 2). We were surprised that we did not isolate a lot of *Spumella*-like strains, just one when we deviated from our earlier strict criterion of targeting rigid round cells. This suggests that it is probably easier to separate paraphysomonads and *Spumella* from each other by light microscopy than was previously appreciated. However, the limitations of light microscopy, especially in the 19th century, mean that it may never be possible to to reidentify convincingly all ‘species’ originally described as ‘*Monas*’ (see Boenigk 2008) or even assign them all to *Paraphysomonas* or *Spumella*, so some old species names may remain for ever in limbo.

### Vast genetic diversity of *Paraphysomonas* sensu stricto

There are now far more species of *Paraphysomonas* sensu stricto (essentially those with only spine scales) than previously assumed (Finlay and Clarke 1999b). We increased the number from 9 to 32 and there are already >30 more environmental DNA sequences on Fig. 1 for *Paraphysomonas* sensu stricto distinct enough to be separate species, and some of genotypes labelled aff. And some treated here as only subspecies may prove to be worth making new species in future, so there are probably at least 80 genuine *Paraphysomonas* species, probably many more – perhaps several hundred as there is no reason to think that sampling is anywhere near saturation. Thus, excessive taxonomic lumping previously underestimated the number of spine-scaled *Paraphysomonas* species at least tenfold. Nearly all our new species would previously have been lumped in just two ‘species’: *Paraphysomonas vestita* and *imperforata*. No wonder those two ‘species’ were thought to be the most frequently encountered and geographically ubiquitous (Finlay and Clarke 1999b). They were not single species but swarms of separate species, some at least as genetically different from each other as the complete range of variation within the entire order Synurales. Supplementary Fig. S4 compares 15 of our 23 new species that would probably once have been lumped as either *P. vestita* (10 with a dense base-plate rim) or *P. imperforata* (five with plain base-plate rims). Yet these 15 species differ obviously in cell size and shape, scale size and proportions, and in spine tip structure; many are far distant from each other on the tree (Fig. 1). Lee (1978) even argued for treating all as one species just because one strain can live in both marine and fresh water; the common assumption of free movement between these habitats for *Paraphysomonas* generally seems false: Fig. 1 shows that marine and freshwater lineages are phylogenetically rather stable in habitat preference; only one 18S rDNA genotype of the 82 *Paraphysomonas* sensu stricto in Fig. 1 was found in both habitats.

As in many protist groups, there is no evidence whether *Paraphysomonas* is sexual or asexual, so we do not know whether the biological species concept can be applied to them or not. It is therefore most reasonable to use a similar degree of genetic differences for subdividing both sexual protists and those whose population genetics is unknown, as has recently been done in other protist groups where sexuality is unknown but might exist (e.g. Bass et al., 2009, Howe et al., 2011, Glücksman et al., 2013). As sexual eukaryotes with even a few differences in rDNA are invariably separate species, placing those with genuinely non-identical 18S rDNA in different named species is unlikely to be oversplitting. Placing those with identical 18S rDNA in the same species may often be correct, but could be undersplitting if they are really sexual. But if they are asexual the degree of splitting appropriate for making nominal species is necessarily arbitrary, so using this simple objective criterion as adopted here is not conceptually problematic. In general, we found that if 18S rDNA of closely related *Paraphysomonas* strains is unambiguously different by even just one or two nucleotides, we can also reproducibly detect slight differences in scale morphology. Conversely if two strains have identical 18S rDNA their scales are generally extremely similar and in many cases indistinguishable. This means that the rate of divergence in 18S rDNA is approximately similar to that of scale morphology in *Paraphysomonas*, so either can be a good criterion for species demarcation. However, the discrete digital nature of rDNA sequences makes them a simpler and less ambiguous criterion to apply than the continuous qualitative and more statistical variation in scales. We found a few examples where strains with identical 18S rDNA have detectably different scales or differ in habitat (marine or freshwater) and therefore presumably in physiology; we conservatively did not make these morphological or physiological variants separate species, but in a few instances felt it useful to make them subspecies. As only six genotypes were found more than once (one 10 and another four times) there must still be gross undersampling of *Paraphysomonas* and well over 100 species must exist. However, our ability to find some genotypes repeatedly, sometimes on different continents, means that variation is not limitless and the number of species globally worth naming is unlikely to run into thousands as in diatoms, the huge protist group where biological species have been best studied (Amato et al., 2007, Mann and Vanormelingen, 2013). It is possible that we have now identified most of the major lineages, so we would expect a similar future sampling effort for new strain isolation to result in proportionally fewer new species.

### Light microscopy reveals systematic differences within *Paraphysomonas*

It is often said that most *Paraphysomonas* species are indistinguishable in the light microscope from *Spumella*, a phylogenetically heterogeneous array of non-scaly heterotrophic chrysomonads abundant and diverse in freshwater. As some *Spumella* grow like weeds in culture, we were initially concerned that isolating colourless chrysomonads at random would yield cultures that would mostly turn out to be *Spumella* (irrelevant to this project), and we would only realise this after the efforts of purifying, sequencing or examining them by TEM. *Spumella* cells are elongated or irregular in shape (perhaps because not so constrained by scales) and some are very small, so we initially focused on culturing large (∼≥7 μm) completely round colourless cells with two visible cilia (one long, one short: to avoid *Oikomonas* (Cavalier-Smith et al. 1996) which lacks the short one), and with a stalked stage. To our surprise this successfully biased the results against *Spumella*, despite often not being able to see scales clearly in the light microscope. This mode of selection has almost certainly given us a biased sample of paraphysomonad diversity – against smaller, unstalked cells or irregular-shaped ones, or any (if such exist) without a short cilium, and could be one reason why we obtained so many *Paraphysomonas* and only one *Clathromonas*, which was found only later in the project when we targeted smaller cells to see if this would yield other scale types. The rarity of Clathromonadidae in our cultures might in part be because they are harder to culture under our conditions, possibly because they need algal food, but that is pure conjecture. In future it should be possible to obtain many more clathromonad cultures for combined TEM/sequencing studies similar to ours for *Paraphysomonas*, since Preisig and Hibberd, 1982a, Preisig and Hibberd, 1982b obtained several uniprotist cultures of six species that we assign to *Clathromonas*. Sequences are now desirable for a greater morphological diversity of *Clathromonas* to test the unity of Clathromonadidae.

Very few *Paraphysomonas* had previously been studied live in the light microscope (Lucas, 1967, Lucas, 1968, Leadbeater, 1972, Pennick and Clarke, 1972, Pennick and Clarke, 1973, Rees et al., 1974); most measurements of previously established *Paraphysomonas* species were on fixed material likely to have shrunk in preparation. Therefore, cellular features visible in the light microscope were not previously used to help distinguish species. Our results show that though not sufficiently detailed to be diagnostic for individual species, variations in cell size map sensibly onto the molecular tree as does long cilium length (×CL). Cell size and cilium length can be characteristic of a set of species, not individual ones. These features may help rule out certain species during initial identification.

With respect to cilium length one can recognise three *Paraphysomonas* clades with longer than average anterior cilium. First, subgenus *Acrospina* having non-dense margin on the scale base-plate with round or oblique pointed spine tips (*P. imperforata, P. lucasi, P. mikadiforma, P. acuminata acuminata, P. acuminata cuspidata*) never has an LC shorter than 2.5 × CL*.* Secondly, *P.* sp. (BZ1) and *P. sinensis* with 2.0 – 2.5 × CL. Thirdly, two species belonging to a larger dense-margin base-plate clade with completely tapering spines, namely *P. longispina, P. stylata limnetica* and *P. stylata stylata* (a subclade of subclade D of subgenus *Paraphysomonas*) have slightly longer LC than the rest in their clade, 2.0 – 2.5 × CL (Table 2). All other species described here have a shorter long cilium (≤2.0 × CL). Stokes’ *P. vestita* had LC 2 × CL like most of our isolates. However, none of our strains exactly matches his other cellular descriptions.

Cell size is indicative of groups of species; formerly misidentified *P. foraminifera* and *P. imperforata* sequences (subgenus *Acrospina*) all have cells below 5 μm, as well as the new closely related species *P. lucasi*. Other small cells studied form two distinct groups: *P. ovalis* and *P. segmenta* in subgenus *Brevispina*, and *P. hebes*, *P. hebetispina hebetispina* and *P. hebetispina limna* are a subclade of subgenus *Hebetomonas*; all these species have average cell sizes between 4.7 – 6.6 μm. Subclade B of subgenus *Paraphysomonas* has some of the largest cells found, averaging 11.3 μm. So, average cell size, as well as scale dimensions and features like presence or absence of concentric annulus and spine tip shape, can sometimes rule out certain species.

Cell behaviour can also differ among species. Some smaller, shorter stalked, species tend to grow as loose patches on the substratum, fixed by a short stalk; some of these exhibit a twitching motion, particularly *P. ovalis*, whilst others like *P.* aff*. imperforata* (EP1) and *P. imperforata* (CCAP 935/13) and *P. lucasi* readily have static LC in a kinked/curved ciliary position. Long cilium motion in the larger-celled species with very long LC is distinct from other species and motion too can be erratic. Other potentially describable behavioural characteristics, especially swimming style, were not noted in this study.

### *Paraphysomonas* spine scale conservatism

There were previously five species of *Paraphysomonas* sensu stricto with holey spine-scale base-plates, but none of our new cultures had base-plate holes or perforations, though we described a sixth such species from published data (*P. perforata*). All other new species have spine scales with entirely unperforated base-plates. Setting aside *P. vestita* (see next section), there were previously only three species of non-holey spine-scaled *Paraphysomonas*: *P. imperforata*, *P. bandaiensis*, and *P. antarctica*. Almost all our 22 new non-perforated species would have been assigned to *P. vestita* (if with dense base-plate margin) or to *P. imperforata* (if no dense margin) prior to our study. Yet we have shown that these crudely defined morphotypes occupy the vast majority of the genetically extremely diverse *Paraphysomonas* clade (Fig. 1), whose genetic depth is comparable to that of any photosynthetic order of chrysophytes (Fig. 2). They are likely to have diverged from each other in the Lower Cretaceous when chrysophyte stomatocysts first appear (∼110 My ago) in the fossil record (Siver and Wolfe 2005). *Paraphysomonas* and *Clathromonas* divergence seems somewhat later (possibly ∼90 My ago, estimated from Fig. 1 short branch taxa); a similar crude estimate places the basal radiation of *Paraphysomonas* sensu stricto at ∼70 My ago. Therefore their basic nail-scale morphology has probably been stable for ∼70 My. This remarkable morphological conservatism in nail-scale structure led to species diversity being grossly underestimated.

### Perforated-scale species diversity

It seems that the single definite perforated species on the tree, *P. perforata*, is a relatively recently derived variant within the large, predominantly unperforated subgenus *Acrospina* (Fig. 1), which would otherwise all have been called *P. imperforata* previously; yet *Acrospina* shows comparable or greater genetic depth to most genera of photosynthetic chrysophytes (Fig. S1). Further research is needed to see if the other five perforate species group with *perforata* or elsewhere; no convincing evidence indicates that perforated base-plates evolved more than once.

Unfortunately, we did not isolate a *P. foraminifera* strain. Two of the three sequences in GenBank labelled ‘*P. foraminifera*’ are so far apart that both cannot possibly be the original species. One was accompanied by a micrograph that clearly shows that it was misidentified (Rice et al. 1997); its spine is only two thirds as long as *P. foraminifera* and tapers only near its tip not along most of its length and base-plate holes are more regular and relatively larger than the intervening trabeculae in *P. foraminifera*. We therefore made it a new species, *P. perforata*. The other sequence AB022864 (strain MBI-HT3, unavailable) has no associated electron micrograph. It might be a genuine *P. foraminifera*, but if it was grown in freshwater medium DY-IV as stated (Andersen et al. 1999), it was probably not *P. foraminifera* which was marine (Lucas 1967), so we placed its name in inverted commas on Fig. 1. As all neighbouring strains to HT3 are freshwater *vestita*-like species, the true *P. foraminifera* most likely will turn out to be related to the marine *P. perforata*, not to HT3. If HT3 actually has a perforated scale base-plate, such perforations must have evolved at least twice in ancestrally unperforated *Paraphysomonas* lineages. The third sequence AF174376 is marine and consistently groups with *P. perforata* (*P. foraminifera* in GenBank; as does Fig. 1, the original paper more wisely labeled it *Paraphysomonas* sp. (Atkins et al. 2000), noting that no scales were seen, so the species could not be identified); the stated cell size (10 – 15 μm not 3.1 – 4.4 μm as in *foraminifera*) proves the GenBank name to be wrong; we assume it was miscalled *P. foraminifera* merely because it was closer to the Rice et al. ‘*P. foraminifera*’ (actually *perforata*) on their trees than to the only other *Paraphysomonas* (an unspecified ‘*vestita*’). Dalby et al. (2008) claimed that *P. foraminifera* is the dominant phagotroph in oil-polluted microcosms, based purely on 18S rDNA sequencing, its closest relative being said to be AF174376, which is not *P. foraminifera*; in fact their abundant sequence PSX4-3 (AY789782) is not closest to AF174376; we found three *P. imperforata* strains and eight environmental sequences with stronger BLAST hits, and on Fig. 1 it groups with *P. imperforata* VS1 (though *P. imperforata* C1 and D1 have the highest and second highest BLAST hits). Clearly it is *P. imperforata*-like, not *P. foraminifera*.

### Phylogenetically significant *Paraphysomonas* spine scale variation

The many more imperforate species are all genetically different, often greatly, sometimes only slightly, and also exhibit subtly different ultrastructural features of their spine scales, whose evolutionary and taxonomic significance had previously been almost entirely overlooked. Nine spine-scale features are useful for identification:

(1) Presence or absence of an inflated/bulbous base to the spine. (2) Degree of spine tapering. (3) Shape of the spine tip: tapered to a blunt tip, rounded tip, oblique sharp, blunt or pinched. (4) Shape of base-plate: oval, round or irregular. (5) Presence or absence of a dense base-plate rim, whose thickness is characteristic within each species. Hibberd (1979) showed by sectioning a *P. bandaiensis* strain, which scale structure suggests probably belongs to subgenus *Brevispina*, that the dense rim is caused by a marginal inflection of the base-plate, but whether that is also true of the somewhat less dense rim of subgenus *Paraphysomonas* is unknown. (6) Occurrence or not of a dense annulus on the base-plate; this is thought to be a slightly raised ring midway across the plate (Lucas 1968). For some strains it is not a consistent character, e.g., *P.* aff. *imperforata* strain EP1 and *P imperforata* strain CCAP 935/14. (7) Crease at the base of the spine present or not, thought to be relic of EM preparation. (8) Presence or absence of radial ribs on the base-plate, possibly also a creasing artefact. (9) Size of scale: length of spine, width of base-plate, and S/P ratio. All these characters are useful diagnostic features.

As shown on Fig. 1, one main feature is rather conservative, and constant within three subgenera: the presence (subgenus *Paraphysomonas*; and thicker still in *Brevispina*) or absence (subgenus *Acrospina*) of the base-plate dense margin. However, the dense margin varies in prominence, some species have more subtle dense edges than others. The fourth subgenus *Hebetomonas* (only *P. hebes*, and *P. hebetispina* studied ultrastructurally) has a distinct spine scale with a truncate/rounded spine tip and circular base-plate whose margin may be slightly dense or not. This somewhat intermediate nature is probably not surprising as *Hebetomonas* is sister to subgenus *Paraphysomonas*, which might have evolved its dense base-plate edge independently of subgenus *Brevispina*. (See Supplemenary Fig. S4 for direct comparison).

We cannot confidently deduce whether the base-plate rim was originally plain as in *P. imperforata* and the rest of subgenus *Acrospina* and the major subclade of subgenus *Hebetomonas* or dense as in subgenera *Paraphysomonas* and *Brevispina* plus *P. parahebes*; but if the topology of Fig. 1 is correct, assuming that a dense rim is ancestral would give only two origins of plain rims, whereas assuming plain rims were ancestral gives three independent origins of dense ones, and so is marginally less parsimonious*.* Though the difference in rim structure is relatively small, it is fairly conservative and numerous clearly related lineages share similar base-plate margins. The margin is most dense and conspicuous in *P. bandaiensis*, which led to its being separated as a species even without sequence information. From our trees it is likely that *Paraphysomonas* ancestrally had scales with imperforate base-plates; they became perforated in the small *perforata* subclade near the base of the largest marine subclade of *Acrospina*, which is sister to *P. imperforata* plus *P. lucasi*.

Within subgenera *Acrospina* and *Paraphysomonas* spine characters further define subsets (Table 2). There are dense-rim (subgenus *Paraphysomonas*) species that all have a small oblique dull spine point. No spines ending in an oblique dull tip are seen in any *Acrospina*. Conversely, there are *Acrospina* species with a sharp oblique tip not found in subgenus *Paraphysomonas*. In *P. segmenta* (subgenus *Brevispina*, to which we suspect *P. bandaiensis* may belong) the dense margin is very wide, whereas others like *P. uniformis hemiradia* have a thinner dense edge – it would be interesting to study the structural basis of these differences by TEM sections.

Variations in shape were more apparent in some species than others. Radial ribs on the base-plate, as in *P. uniformis hemiradia*, have been noted before (Takahashi, 1976, Preisig and Hibberd, 1982a, Eloranta, 1989); though it is unclear if they stem from artifactual regular wrinkling during EM preparation or are a natural rigid structure, their consistent occurrence in one subspecies only means that they must reflect an underlying structural difference from other species. It was impractical to do scanning electron microscopy for so many species, so we chose not to for any. Some studies consider that it can sometimes be less useful and even confuse identification of silica-scaled chrysophytes (Boo et al., 2010, Kynčlová et al., 2010).

Diversity of scale morphology within a species can vary. *P. variosa* is so named because of its unusually large range of scale size and thickness. *P. dimorpha* is the only *Paraphysomonas* so far known with two qualitatively different types of scale: spine and plate. Some *P. dimorpha* cells had only spineless scales or mostly spine scales, perhaps indicating a life cycle stage of the organism. Varying ratios of different scale types are also seen in *Clathromonas butcheri*; like Pennick and Clarke (1973) we noted that some cells had just plate or basket scales or a mixture of both. Both scale variation within a species and the evidence that similar scales can be seen in several parts of the tree make species identification from single scales in environmental samples, sometimes done (Finlay and Clarke, 1999a, Finlay and Clarke, 1999b), necessarily less precise than from whole cells, and sometime more ambiguous than previously realised.

Our study emphasises the importance of noting subtle but consistent differences in scale structure when identifying *Paraphysomonas* or describing new species (see Scoble and Cavalier-Smith 2013). Their neglect led to their previous excessive lumping: predominantly just ‘*vestita*’ with a dense base-plate border and *imperforata* lacking it, by far the two most frequently encountered ‘species’ (Finlay and Clarke 1999b); this difference actually characterises two huge species-rich groups that differ genetically as much as do whole families in many eukaryotes. These crudely differentiated morphotypes cannot be real species; the ‘*vestita*-like’ morphotype is a clade, the entire new subgenus *Paraphysomonas*, and the *imperforata* morphotype constitutes the majority of the new subgenus *Acrospina* apart from the structurally derived *P. perforata*. This makes all previous records of the distribution and ecological preferences of *P. vestita* and *P. imperforata* meaningless at the species level; they pertain only to the whole subgenus *Paraphysomonas* and most of subgenus *Acrospina* respectively – too crude to be useful. Species with holey base-plates like *P. foraminifera* and *perforata* appear to be strictly marine and have not been reported from freshwater or soil.

If variations in scale morphology are compared across the tree it is clear that in some regions small variations in scales correspond with small variations in sequences and can often be similarly resolving to DNA sequences, if proper note is made of these small structural differences. We found one case where scale differences seem to be more resolving than sequences (*P. vulgaris* strains PML4B and PML8 have different spine lengths – see Supplementary Information 1), but several instances where quite similar scales were present on rather distant lineages. Thus, though there is often a broad (and sometimes quite close) correlation between scale and genetic differences, this is not a precise correlation and sequences in general offer a more reliable picture of *Paraphysomonas* evolution and affinities that is less subject to convergent evolution (e.g. with respect to the appearance of the base-plate margin and spine tips). The new species described here can probably all be distinguished by scale morphology alone if whole cells are available (but not from single scales). But as undescribed species might have indistinguishable scales and the full range of variation for most rDNA genotypes is not yet known, more reliable identification needs sequences in addition to or instead of scale morphology. Electron microscopy alone would be less resolving for environmental surveys of *Paraphysomonas* sensu stricto than DNA sequencing but would advantageously have different biases.

### *Paraphysomonas vestita* identification problem

We found a very large number of genetically often extremely different species of *Paraphysomonas* having nail-like scales with non-perforated entire round base-plate with a dense margin and relatively long, more or less pointed, central spine. Previously all such species were lumped together as *Paraphysomonas vestita* despite the different dimensions and differences in scale morphology we discovered. Are any of our strains *P. vestita* as described by Stokes (1885) under the name *Physomonas vestita*?

*Physomonas vestita* was described before electron microscopy, therefore we can only use features visible in the light microscope in identification: cell size, shape, and cilium, stalk, and spine lengths. Stokes only observed sessile and stalked cells from shallow ponds and streams in New Jersey, USA; 1/1666 inch in diameter, equal to ∼15.2 μm, not 11 μm as incorrectly stated by Manton and Leedale (1961), who made the first electron microscope study of a *Paraphysomonas* in a mixed protist culture, but studied only non-sessile cells. Stokes did not say whether his measurement included or excluded the spines, but as the longer cilium was said to be twice body diameter he must have excluded spines for that to be true, if his drawing is accurate. His drawing depicts spines approximately 81% of the length of the shorter cilium, said to be ‘one-fourth’ that of the longer one*.* These numbers may be approximations to simple fractions, not precise measurements; recurring decimals in his diameter seem likely to result from calculating from a starting measurement with perhaps only one significant figure, so may only be approximate. Taking them literally makes *P. vestita*'s long cilium ∼30 μm, short cilium ∼7.6 μm, and stalk ∼61 μm. The stalk in the drawing is actually ∼3.4 – 3.6 X the body diameter (width slightly less than length) not 4X, giving an idea of likely rounding approximations in his descriptions/drawings. From spine length/cell diameters in the 1885 drawing, the spines would be ∼6.3 – 7.5 μm long, but if we compared them instead with the short cilium, assuming its length was accurately shown, we would get ∼9.4 μm. Stokes's (1885) text description is identical except for omitting detail concerning contractile vacuoles, but the figure is redrawn with proportionally shorter posterior cilium (closer to proportions in text) and a somewhat less circular cell; from it similar calculations indicate spine lengths 4.9 – 6.4 μm); because of the discrepancy between the spine-length/long cilium (LC) ratio in the two figures we base our *vestita* spine length estimates on cell diameter/spine lengths, not spine length/LC length. Given the excessive lumping as *P. vestita* of different genetically unrelated strains that our trees reveal, and the comparably excessive lumping of strains with substantially different scale morphology discussed below, the concept of what *P. vestita* is has clearly been far too vague in the past, making it desirable to establish a neotype noting precise cell and scale measurements to stabilise nomenclature.

For a neotype to be established, the type strain should be from fresh water (ideally from the USA as was Stokes's, not Europe) and should have a sessile cell size range around 13 – 17 μm with mean close to 15 μm and ∼5 – 7.5 μm spines that are very conspicuous in the light microscope. We have not made a neotype primarily because, as Table 2 shows, none of our strains has a mean diameter as great as 15 μm, and those with the largest most conspicuous spines did not have the largest cells. Our largest strain is *P. uniformis uniformis* with mean diameter 11.6 μm but its spines are only 4.5 μm, so it is probably not *P. vestita*. The freshwater strain of Manton and Leedale (1961) was 12 – 20 μm and its spines 2 – 10 μm, so might have been *P. vestita*, though its extreme variation in spine lengths makes us doubt that. Manton and Leedale's cilium measurements differ from Stokes’ in that the LC is longer (40 – 45 μm not 30 μm). A better candidate for *P. vestita* is that carefully studied by Korshikov (1929), who first showed the nail-like morphology and siliceous nature of the scales, though as his strain and that of Manton and Leedale (1961) had just one contractile vacuole, not two like Stokes’, and a shorter short cilium and longer long cilium, we cannot be sure of that. Bec et al. (2010) figure a *Paraphysomonas* identified as *vestita* that is 22 μm in diameter and with much more conspicuous spine scales under DIC than most of those described here. Thus, some other authors have found larger strains more like Stokes’ *P. vestita* than any we isolated, so there is no reason to doubt the accuracy of his observations.

Four 18S rDNA sequences are labelled *P. vestita* in GenBank, all probably from misidentified strains. AF109325 was from a strain recloned from a contaminated derivative of CCAP 935/14 isolated from the eutrophic freshwater pond Priest Pot (Caron et al. 1999); as its scale spines were approximately 3 μm long it cannot be *P. vestita*. This sequence and two ‘*Spumella*-like’ (AB616676, AY651094) sequences share a large (55 nt) insert and just differ by a few presumed sequencing errors, we have made all of these *P.* aff. *caroni* because they are so close to our new more deeply branching species. A different, closely related sequence GU220392 from marine strain J1 with much thicker spines 3.0 – 4.3 μm (Petronio and Rivera 2010), is also not *P. vestita*, so we made it new species *P. petronia*. GenBank sequence Z28335 (Rice et al. 1997) is an extremely different sequence from a marine strain with spines ∼6 μm long, but more like *P. vestita* (Manton and Leedale, 1961); however, because of the slenderness of its spines, lack of light microscope evidence of its cell and ciliary dimensions, and its marine habitat (Rice et al. 1997), we do not accept it as *P. vestita.* Therefore, we call Z28335 *P.* aff*. longispina* because its 18S rDNA is just one nucleotide different from our new species *P. longispina* with similar scale dimensions. *P. longispina* had an obvious scale-base layer but spines were not obvious. We isolated another freshwater and two marine strains with identical 18S rDNA sequence to *P. longispina*, making it the first found in both freshwater and marine environments. Even so, we do not know if the same isolate can grow in both marine and freshwater environments. In principle, even two strains with the same 18S rDNA sequence could be different species with contrasting ecology and other genetic differences.

Until genuine fresh water *vestita*-like strains are cultured clonally and shown to be genetically and scale-morphologically indistinguishable from marine strains, it is unwise to assume (as sometimes done: Finlay and Clarke 1999a) that *P. vestita* can grow in seawater. A short fragment FJ886745 (348 nt), from a strain from the Marine Biology Laboratory of Copenhagen University (Bochdansky and Huang 2010) and therefore presumably marine, is nearly identical (one T insert) to both Z28335 (*P.* aff. *longispina*) and AB022864 (‘*P. foraminifera’* of Andersen et al. 1999, whose identity we questioned above, but which differs elsewhere in the molecule from *P.* aff. *longispina*). Because the AB022864 fragment is not associated with evidence for spine length, we cannot say whether it could be *vestita*, but is probably not. No evidence is published where we are sure a ‘*vestita*’ 18S rDNA sequence is from a correctly identified strain; this strain should also not be treated as representing genuine *P. vestita*, given that both 18S rDNA and scale structures give evidence that almost every previously studied strain identified as *P. vestita* is a different species.

Scales seen in the three just cited TEM studies are clearly different from each other, and different from the (possibly correctly identified) ‘*P. vestita*’ of Manton and Leedale (1961); those of 10 further publications are all different from the aforementioned as well as from each other (Dürrschmidt and Croome, 1985, Finlay and Clarke, 1999a, Jacobsen, 1985, Kristiansen, 1989, Kristiansen, 1992, Lee and Takahashi, 1993, Preisig and Hibberd, 1982a, 1982b; Štefanová and Kalina, 1992, Takahashi, 1976). From these examples alone, spine lengths range from 1.2 to 10 μm and most would not have been visible in the light microscope because they are so small, and are therefore not examples of *P. vestita*, e.g., in Fig. 1D of Finlay and Clarke (1999b) spines are only 2 μm. Except for Manton and Leedale (1961), none of the many publications showing scales identified as *P. vestita* provides sufficient evidence, even from light microscopy, that these specimens are from a cell like the original *P. vestita*; the resemblance of these scales to Manton and Leedale's are of a greatly generalised likeness and too imprecise to be evidence that they were from the same species. Past records of *P*. ‘*vestita*’ are best regarded as of the whole subgenus *Paraphysomonas*, not any one species.

### Past lumping of *imperforata*-like spine-scale morphotypes

*P. imperforata* (Lucas 1967) was a small (4.5 μm) marine strain with very small scales with plain-rimmed circular base-plate with lightly distinguished annulus. Because its scales lack strongly distinctive characters, almost all unperforated spine scales without a thick rim have been identified as “*P. imperforata*”, a gross lumping as for *P. vestita* (Scoble and Cavalier-Smith 2013). These over-generalisations led to the incorrect belief that “*P. imperforata”* scales have an added attribute, not in the original description, of an oblique sharply pointed tip (Preisig and Hibberd 1982a), as here described for *P. acuminata acuminata*, which would have been formerly lumped in *P. imperforata* despite spines being more than ten times longer than Lucas's (1967) original description. By using genetically characterised clonal cultures we have shown how finer ultrastructural details, e.g. overall size, spine length and tip shape, can be used to help define numerous species and for identification. Without sequences and clonal cultures it would not have been possible to interpret the significance of these subtle differences in scale morphology and rectify the gross taxonomic lumping that predominated in the past.

### Monophyly of Paraphysomonadida and relationship to major environmental clades

Our Bayesian analyses suggest that major environmental clade 1 as defined here is probably related to paraphysomonads and may even branch within them (Fig. S2): in that Bayesian tree environmental clade 1 is sister to Paraphysomonadidae alone. However, the precise branching order of the 10 major chrysomonad clades revealed here (two environmental and two paraphysomonad; six of predominantly phototrophic chrysomonads) varies with algorithm and taxon sampling. Five of these clades only correspond with presently established ancestrally photosynthetic orders (Chromulinales, Hibberdiales, Ochromonadales, Synurales, Hydrurales), of which at least two have secondarily heterotrophic derivatives not directly related to either of the two purely heterotrophic paraphysomonad clades or to either major environmental clade. The two paraphysomonad families (Paraphysomonadidae, Clathromonadidae) are almost as mutually divergent as are the four ordinally ranked primarily photosynthetic clades. The lack of known phenotype for two major clades means that we can neither say whether Paraphysomonadida as currently circumscribed is monophyletic or reconstruct the ancestral chrysomonad phenotype until the organismal character of both major chrysomonad purely environmental DNA clades is determined. In particular we need to know whether EC1 consists of silica-scaled scaly heterotrophs like other paraphysomonads or of phototrophs, scaly or otherwise. If as is possible it consists of scaly heterotrophs, it might include some of the species formerly included in *Paraphysomonas*, but here excluded because of very different scale morphology, in which case it might be appropriate to consider this clade a third paraphysomonad family. A major conclusion of our analyses that was not previously apparent is that there are fewer radically distinct chrysomonad clades comprising only environmental sequences than previously thought (del Campo and Massana, 2011, Charvet et al., 2011) – just the two here called EC1 and 2. Our better heterokont sampling also makes it clearer than before that both clades are genuinely more closely related to known chrysomonads that to the closest outgroup (Picophagea). This conclusion may help future interpretations of these unknown organisms as it suggests that a broad appreciation of their significance could come from culturing and sequencing just a few. Though the nature of these clades is highly relevant to the question whether Paraphysomonadida is itself a clade or polyphyletic, we have placed a more detailed discussion of our findings concerning them in the supplementary material to save space (see: supplementary information part two). The poor basal resolution of the chrysomonad rDNA tree means that multigene analyses will probably be necessary to establish the relationships amongst the 10 major clades more securely. Our trees revealed at least seven distinct losses of photosynthesis in Chrysophyceae, so the class should not be thought of as typically algal, but as ancestrally phagophototrophic with independent multiple losses of photosynthesis or phagotrophy.

### Environmental, ecological and biogeographic questions

No environmental clones appear in the large long-branch subclade of subgenus *Paraphysomonas*, but they are found in all short-branch clades, exactly as in Heliozoa (Cavalier-Smith and von der Heyden 2007), which may similarly stem from PCR bias. PCR of environmental DNA may have missed these clades of *Paraphysomonas* if primers were not specific enough for these cells, but more likely bias arises because the long-branch clade has 18S rDNA insertions making amplicons longer (as in long-branch Heliozoa). It is most unlikely that all strains in this clade were so rare in all environments that rarity alone explains their absence from environmental DNA data, especially as they include the ‘common’ clade isolated most often. This example of how culturing reveals an entire clade of *Paraphysomonas* never picked up by general environmental DNA cloning emphasizes the necessity of using both culturing as well as environmental PCR to assess protist biodiversity, as previously found in Cercozoa (Bass and Cavalier-Smith, 2004, Howe et al., 2009) and Heliozoa (Cavalier-Smith and von der Heyden 2007). It would be valuable to create a primer specific to this clade for environmental probing to uncover its diversity, especially because culturing can be so cumbersome and limited to cells that can live in laboratory conditions; that culturing itself can be biased is well known – Lim et al. (1999) found that *Paraphysomonas imperforata* is disproportionately represented in bacteria-rich marine enrichment cultures.

Present evidence for the named 32 morphospecies is insufficient to decide whether any are cosmopolitan, but we found the same 18S rDNA genotype in multiple countries for several, suggesting that these ones may be very common and widespread and perhaps distributed world-wide at least in temperate zones. However, another source of bias in interpreting biogeographic data on protists arises because in the past few hundred years human transport of soil (e.g. with plants or on shoes or vehicles) and water (e.g. bulk ship ballast) will have sharply increased their rate of global mixing, so some cases of protist cosmopolitanism will be as anthropogenic as that of agricultural weeds or deliberately introduced species (Aguilar et al. 2014). With only geographically sparse records so far for specific *Paraphysomonas* genotypes we cannot say whether these few widely distributed samples reflect natural dispersal or are distorted by unwitting human transport. Denser genetic sampling across several continents is essential for clarifying *Paraphysomonas* biogeography, which remains an almost entirely open question.

A claim to have deduced cosmopolitanism for *Paraphysomonas* sensu lato (Finlay and Clarke, 1999a, Finlay and Clarke, 1999b) based on finding a high proportion of named species in one pond (Priest Pot, Cumbria) is invalidated by the previously excessively coarse taxonomy, as the previous gross lumping of species revealed by our study necessarily biases conclusions in favour of cosmopolitanism over endemism (Foissner, 2006, Patterson and Lee, 2000). Similar studies are needed for *Clathromonas*, which make up the majority of the paraphysomonads recorded in Priest Pot. As discussed above, *C. butcheri*, originally from salt marsh pools, has been subject to excessive lumping. We therefore doubt whether most (if any other than the original description) records for this species were correctly identified. We suspect that (as we found for *Paraphysomonas* sensu stricto) closely related but genetically distinct saline and freshwater species may exist. The same could be true of other Priest Pot *Clathromonas* originally recorded from marine habitats. We suspect that a substantial fraction of these records may be of still undescribed species. That was certainly true of the majority of the new species of Heliozoa described by Cavalier-Smith and von der Heyden (2007). Culturing/genetic/TEM studies on *Clathromonas* would test this surmise.

Fig. 1 shows that habitat preferences for marine, freshwater and soil environments are phylogenetically quite strongly conserved in *Paraphysomonas*, i.e. major subclades are habitat specific and rather few evolutionary shifts between marine and freshwater/soil are evident. Supplementary Fig. S3 shows this to be equally true of both major environmental DNA clades of chrysophytes. Strong phylogenetic conservatism with respect to occurrence in marine versus freshwater habitats exists in numerous other protist groups (Cavalier-Smith and Chao, 2012, Cavalier-Smith and von der Heyden, 2007, Glücksman et al., 2013, von der Heyden et al., 2004, von der Heyden and Cavalier-Smith, 2005), so it is unsurprising that it is also true of *Paraphysomonas*. The largest subgenus *Paraphysomonas* was clearly ancestrally freshwater (as its two deepest subclades exclusively are); later invasions of soil and marine environments were rather few. The whole genus *Paraphysomonas* displays relatively few such habitat switches, but it is harder to decide whether their ancestor was freshwater (somewhat more likely) or marine. Invasions of soil seem even rarer, perhaps only one in subgenus *Paraphysomonas* (assuming that the freshwater habitat of *P. stylata limnetica* is a secondary reversion of an ancestrally marine subgroup). The literature has often stated that *P. vestita* can inhabit marine and freshwater environments, but this pertains only to the excessively generalized morphotype that *P. vestita* had previously.

Stokes (1885) did not say what his *P. vestita* ate, but that of Manton and Leedale (1961) fed on a variety of smaller microorganisms, especially the haptophyte *Chrysochromulina*. Korshikov's strain ate the large photosynthetic bacterium *Chromatium okeni*. If *P. vestita* prefers photosynthetic prey, that might explain why we did not find it. Though seven of our cultures, all from the subgenus *Paraphysomonas* and mostly from soil, also included smaller colourless chrysomonads (whether *Oikomonas-*like contaminants or odd shrunken forms of the *Paraphysomonas* itself was sometimes unclear), none contained eukaryotic algae, and our strains subsisted solely on heterotrophic bacteria (or on these ‘contaminants’/reduced forms) (less likely as saprotrophs, as no organics were added). If smaller *Paraphysomonas* can survive on a diet of heterotrophic bacteria but larger ones require a diet including eukaryotes or photosynthetic organisms, our culturing method would have prevented our isolating them and explain why we found no *P. vestita* or any other similarly large but undescribed species. We cannot exclude the possibility that some of our strains could eat eukaryotes (a few were cannibals), or that if they did their cells would be larger, but suggest that a broader range of food organisms should be used in future in an effort to obtain *P. vestita* for sequencing and probably a different set of species from those we found. Not supplying the right food could be why we isolated only one *Clathromonas*, and none of the former *Paraphysomonas* now excluded from both *Paraphysomonas* and *Clathromonas*.

## Conclusion

We have shown that *Paraphysomonas* taxonomy formerly suffered from excessive lumping at species and generic levels. Restriction of the genus to species with nail-like scales, and demonstration that previously over-looked subtle differences and finer details in scale ultrastructure correlate with robust sequence phylogeny, provide a sounder basis for future studies of the biodiversity, ecology, and biogeography of *Paraphysomonas* sensu stricto. Similarly detailed studies are needed for *Clathromonas* and other genera segregated from *Paraphysomonas*: greater culturing efforts for them and the two major environmental clades are essential to improve understanding of their large-scale evolution. Environmental DNA studies using group-specific primers are needed to estimate the true biodiversity of former *Paraphysomonas* morphotypes, which might collectively have hundreds of species

## Figures and Tables

**Figure:**
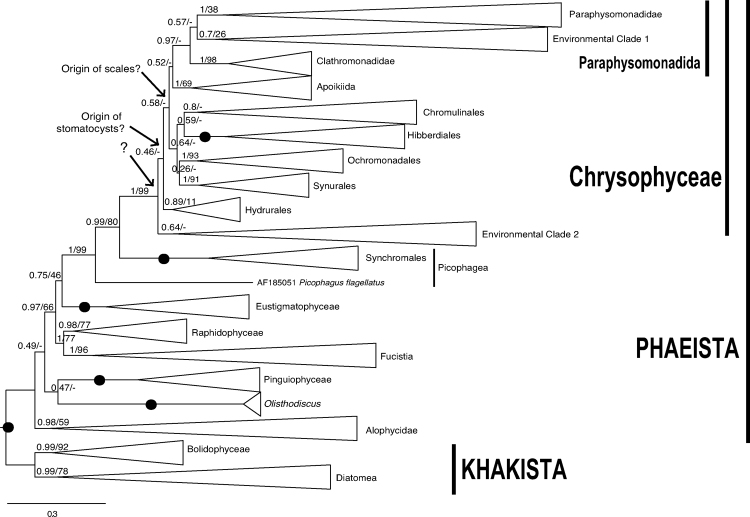


**Figure:**
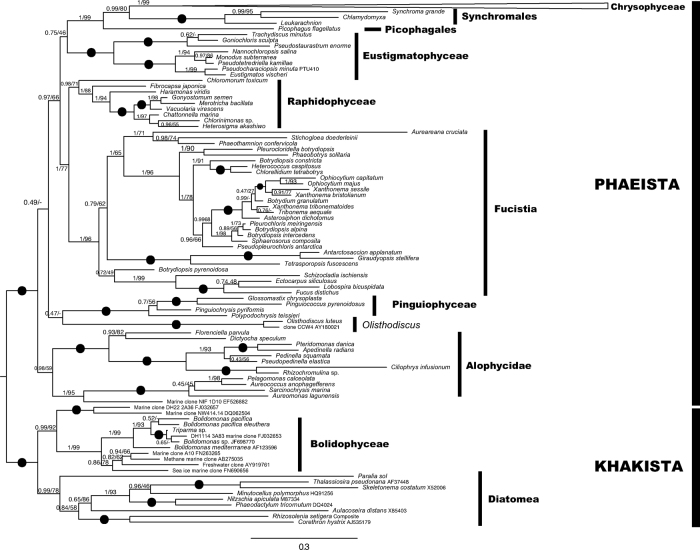


**Figure:**
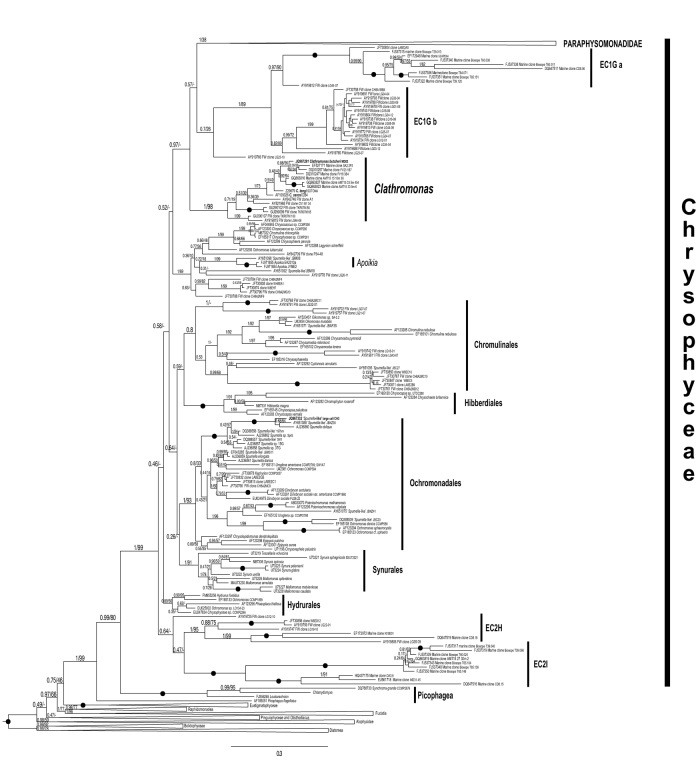


**Figure:**
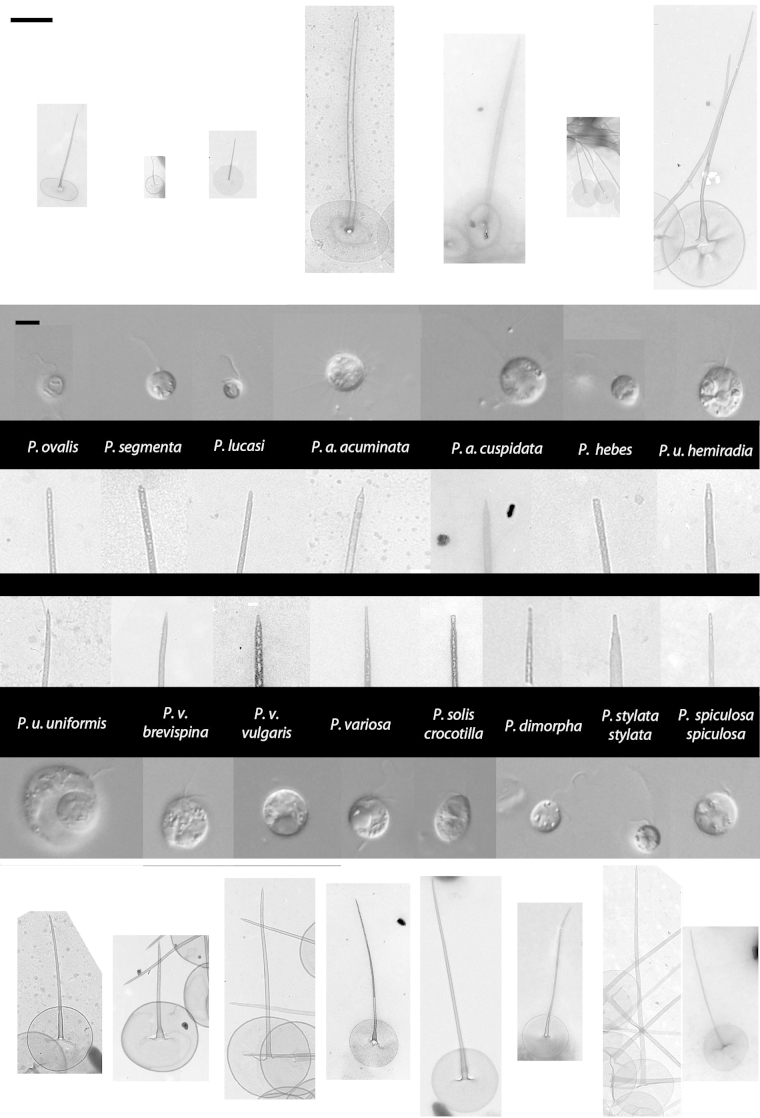

