# Supplementary material for: Scale evolution in Paraphysomonadida (Chrysophyceae): Sequence phylogeny and revised taxonomy of Paraphysomonas, new genus Clathromonas, and 25 new species
Source: Eur J Protistol. 2014 Oct;50(5):551–92. doi: 10.1016/j.ejop.2014.08.001 (PMC4238902; doi:10.1016/j.ejop.2014.08.001)
Supplement: Supplementary file 1 [file mmc1.docx]

**Supplementary Information:**

PART ONE. Species and Strain Comments

PART TWO. Discussion: Major environmental chrysophyte clades

and REFERENCES

**_______________________________________________________________________________**

**PART ONE. Species and Strain Comments**

**Subgenus *Brevispina***

***P. ovalis*** sp. n. (ArbSmall) **Comment:** Unlike in most *Paraphysomonas* species, *P. ovalis* cells attached to the bottom of the Petri dish in young sparse cultures are not randomly arranged, but occupy local patches within which cells are solitary and well separated but separated from similar patches by extensive empty areas. This local patchiness might arise from daughter cells moving only short distances after they separate before attaching, making a local patch a single recently multiplied clone. Probably species that do not show this patchiness swim for much longer before settling and so become randomly distributed spatially. We refer below to a few other strains with similar patchiness as ‘patchy settling’.

***P. segmenta*** sp. n. (KU3b2) **Comment:** Occasionally exhibit patchy settling. Cells sometimes appear indented or flat at the base of cilia. Sessile cells occasionally jerk once and are then still. A small ‘pinch’ at the base of the cell, where the stalk is attached, sometimes gives a tear-drop appearance, especially when LC beats strongly. *P. segmenta* resembles *P. bandaiensis*, *P. truncata*, and *P. porosa* sp. n. in its base plate with a thickened margin caused by an inflexion. The non-tapered distal segment of *P. segmenta* spines ends in a rounded tip, unlike the truncated end of *P. truncata*. *P. bandaiensis* non-tapered spines have rounded tips, but its scales are much smaller; the spine is nearly a third smaller than in *P. segmenta*, base plate diameter nearly half. *P. bandaiensis* cell was 1 µm smaller and its LC over twice as long, but comparison may not be meaningful as Takahashi did not state whether he measured live cells or TEM preparations where they would be smaller through shrinkage during drying onto the grid. A shorter environmental sequence **A42** (AY642748: Lefranc et al 2005) from a freshwater lake has just two nucleotide differences and a deletion, the latter probably a sequencing error because it is in a well-conserved region near the end. These two sequences share some very distinct signatures, so we call A42 *P.* aff*. segmenta*.

**Subgenus *Acrospina***

***P. acuminata acuminata*** sp. n. (PML6A) **Comment.** Oval to pyriform cells absent. Spines barely taper and the upper range of thickness of the tip reaches beyond the minimum thickness of the base; slight tapering is detectable, but hardly noticed. Isolate ‘**CCL3C**’ (Oxford, UK. JMS) is very close to *P. a. acuminata* (possibly identical, but as three nucleotides were ambiguous - ‘n’ we call it aff. *P. a. acuminata*). CL 8.2 (8.6-9.1 n=4). The cells look very similar, as do the scales; spine 5.4 µm (4.5-6.9), base plate 2.1 µm (1.7-2.2) 18S rDNA sequence GenBank JQ967328. An additional isolate, **WA20KP** (aff. *P. a. acuminata*, from Austria. JB), with just two definite nucleotide differences, is a similar size and has the same spine scale morphology with similar dimensions CL 9.5 µm (7.3-11.4: N= 5); LC x 3.5 CL, SC x 0.75 CL. Spine 5.3 µm (4.1-6.5), base plate 2 µm (1.9-2.1), S/P ratio 2.6 (2.0-3.3). Spine base width; 0.157 µm (0.14-0.18), spine tip width; 0.091 (0.07-0.13). 18S rDNA sequence GenBank JQ967327. We do not give it a separate name as its scales are indistinguishable.

***P*. *mikadiforma*** sp. n. (JBM02) **Comment.** JBM02 is a mixed culture of *Paraphysomonas* species with different spine scales. *P. mikadiforma* is identified through its close phylogenetic relationship with *P. acuminata* *cuspidata,* subsp. n. and *P. a. acuminata* sp. n., which both have similar scale morphology and cellular characteristics, particularly the roundness of the cell and LC being > 2.5 x CL. The other *Paraphysomonas* in the culture exhibits a tapering spine with a strong inflexion at the margin of the base plate, spine length 4.3 µm and base plate 1.25 µm (two scales observed), closely resembling *P. crocotilla*. (Figure 10, A-F). Other colourless cells in the culture were more ovoid in shape and had shorter LC, perhaps twice CL. A very closely related published sequence, AY651096, strain JBM06 (Boenigk et al. 2005), which has a few undetermined nucleotides, one in a variable region, different from *P. mikadiforma*, was also isolated from Lake Mondsee, and so is probably the same species because they share distinct signatures along the sequence. We regard AY651096, strain JBM06 as *P.* aff*. mikadiforma*.

***P*. *lucasi*** sp. n. (NC10-16) **Comment:** Lucas described the spine as tapering, but it is unclear what Lucas meant by ‘tapering to a point’ - his figures do not make it clear whether the tip was sharp or blunt, or rounded like *P. lucasi*, so we cannot precisely compare scale morphologies. Below we describe two marine isolates more similar than *P. lucasi* to the original *P. imperforata*, which differ from it so subtly that they must either be the same species or extremely closely related. One (CCAP 935/14) was previously identified as *P. imperforata*, probably correctly, and has 3 nt 18S rDNA differences to our new strain below:

***Paraphysomonas*** **aff. *imperforata.*** (strain EP1) **Comment:** Figure 5C-D. CL 4.7 µm (3.2-6.8 N=44); LC 2.5 x CL; SC 0.75-1 x CL. Small bright round to irregular cell commonly attached to substratum via short stalk, evenly spaced cells. Swimming cell fast and often elongate just as common as sessile. LC is often seen static, held curved and kinked. SC is conspicuous. One type of spine scale. Spine barely tapers, sometimes showing ‘shoulder’ along shaft with rounded tip. Spine protrudes from round to oval base plate. Spine 0.79 µm (0.65-0.89), spine base width; 0.042 µm (0.035-0.048), spine tip width; 0.020 µm (0.012-0.30). Base plate 0.71 µm (0.65-0.88) inflexion sometimes visible at margin mostly absent, annulus sometimes seen. S/P ratio 1.1 (0.9-1.2). Strain **EP1**. (Porlock harbour, Exmoor National Park. UK. JMS). Marine, muddy flats. Its 18S rDNA (1669 nt) was not included in Figure 1 as it is identical to four others except for a few probable sequence errors.

The four ‘*P*. *imperforata*’ sequences included on the tree 18S tree (EF432518 (C1), EF432519 (D1, AF109323 (VS1) AF109324 (SR3)) are probably all actually identical except for a few sequencing errors that misleadingly prevent their grouping on the tree. Our strain is similar to the original description of *P. imperforata*: both are marine cells of similar size and cilia lengths. Lucas did not mention a static LC, common in this isolate. The biggest difference between the scales is that this strain has a slightly smaller scale base plate that only sometimes exhibits an annulus (always present in *P. imperforata*). Lucas described the spine as tapering, but it is unclear if it was to a point or rounded or if there was a segmented element to the tapering (shoulder); his figure D suggests that the basal half may be less tapering than the distal half (as usually in ours); its apparent greater thickness is probably attributable to metal shadowing. As we cannot be totally sure it is equivalent to Lucas’ organism, and the frequent absence of annulus suggests it may not be, we call it aff. *imperforata*. The single scale of ‘*P. imperforata’* in Caron et al. (1999) is morphologically indistinguishable from those of EP1 and has a base plate diameter of 0.88 µm; but it was not stated which of the three different imperforata strains they sequenced it came from. Both those in GenBank (VS1. SR3) are in our tree.

**Strain** **CCAP 935/13 *P. imperforata.* Comment:** (coll. 1983 Fenchel) from Aarhus Bay, Denmark, resembles very closely Lucas’s original description; its scales are extremely close in size, although, as in EP1, an annulus is not always present (Figure 5, E-F). CL 4.5 µm (3.2-6.3 N=23); LC 3-3.5 x CL; SC 1 x CL. It has slightly longer cilia, but behaves similarly to EP1. The 6 sequences identified as *P. imperforata* in Figure 1 are clearly all related, but are part of a 9-sequence clade (present on both Bayesian and ML trees). Inspection of the alignment suggests that they probably represent at least 4 distinct species with *P. imperforata*-like scales; probably all are marine though for two there is no published information. The sequence of the CCAP strain is clearly distinct from the four probably indistinguishable strains mentioned in last paragraph. But the partial sequence Z29680 (Rice et al. 1997) is identical in its region of overlap with all the other five sequences labelled *imperforata* so its position on the tree is obviously arbitrary (Figure 1). Its scale spine length was similar to the CCAP strain not to EP1. As Table 2 indicates, scale spine and base plate dimensions differ between CCAP 935/13 and EP1, which are consistent with the genetic differences; both suggest that they are probably different species. Though data are too limited to be sure, for now we regard the CCAP strain as the same species as Lucas’. Probably the cluster of five species with sequences represents new species but until more data are available we call them all *P.* aff. *imperforata*. Note that marine environmental sequence from an anaerobic habitat (AY180017) that is sister to the *imperforata*-like clade on the Bayesian (but not ML) tree is wrongly annotated as a diatom in GenBank (Stoeck and Epstein 2003).

***P*. *perforata*** sp. n. (SOTON-A) **Comment**: The very short (326 nt) *P. foraminifera* sequence (GenBank Z33646; confusingly stated to come from the same strain SOTON A as their *imperforata*) (Rice et al. 1997) is in our tree (Figure 1). Furthermore, a closely related strain at the base of this small *P. perforata* clade is TPC2, isolated from a hydrothermal vent by Atkins et al (2000) who saw no scales and could not identify the species, yet submitted the sequence to GenBank in 2000 as *P. foraminifera* (AF174376) probably because of its 99.6% sequence similarity the SSU of *P. ’foraminifera*’ (Z38025), now *P. perforata*. There are 8 differences between these strains, so it would be reasonable to recognise them as different species; this strain might even be a real *P. foraminifera* Lucas (1967), but without scale data we cannot identify it or make a new species; but it should no longer be called *P. foraminifera*. It is more likely that scales of TCP2 were lost in preparation than it is really a naked *Paraphysomonas*.

**Subgenus *Hebetomonas***

***Paraphysomonas hebes*** sp. n. (IND1) **Comment:** The only published one with a truncate tip is *Paraphysomonas truncata* (Preisig and Hibberd 1982), which had a non-tapering striated spine with a prominent thickened rim to the base plate, and also an annular ridge. *P. truncata* and *P. hebes* sp. n. are likely close relatives, especially since cell size (4-6 µm and 3.2-5.9 µm, respectively), overlaps and spine scale dimensions are similar.

***Paraphysomonas hebetispina hebetispina*** sp. n. (NC10-20) **Comment:** Scale spine tips vary from rounded to flatly truncate; some spines appears slightly thicker than others. Annulus observed, but not for all scales. This species differs from *P. hebes* in SSU (17 nt differences) and presence of annular fold in the base plate of the scales and tip variation, but scale dimensions are very similar.

***Paraphysomonas hebetispina limna*** subsp. n. (PML2A-e2) **Comment:** When the culture was alive it was difficult to observe cilia at x40 unless one agitated the dish to move cells, perhaps because of their orientation: the cilia can easily be overlooked.

***Paraphysomonas parahebes*** sp. n. (HFlag) **Comment:** This species was originally misidentified as *P*. *bandaiensis* and was the only sequence with this name, therefore no 18S rDNA sequence of a genuine *P. bandaiensis* is available to see, or where its very small scales fit on the tree; they might be in the subgenus *Hebetomonas* clade like the misidentified strain, but it is also possible that *P. bandaiensis*-like scales are more like those in subgenus *Brevispina* as the cells are of similar dimensions and the scales very small with a prominent inflexed margin; however the long cilium is shorter in known *Brevispina* species than in *P. bandaiensis* Takahashi (1976)*.*

**Subgenus *Paraphysomonas*, clades A-E:**

**Clade A:**

***Paraphysomonas uniformis*** ***uniformis*** sp. n. (WA28KT) **Comment:** Cells can become large in culture, especially when preying on smaller *Paraphysomonas*, large bacterial clusters, and very small (*Oikomonas*-like) contaminant. *P. u. hemiradia* also has long spine scales from a large plastic cell, but has some scales that are obviously different: *P. u. uniformis* has a narrower spine base as well as tip and no radial ribs. Some aberrant scales of *P. u. hemiradia* resemble *P. u. uniformis*. Another strain **WA32KAG** (Lake Wallersee, Austria. JB) with smaller cells, 8.1 µm (6.4-10.5), 18S rDNA partial sequence **GenBank** JQ967319, and ‘uncultured eukaryotic picoplankton clone **P1.35**’, **GenBank** AY64271 (Lake Pavin, Massif Centrale, France) have identical 18S rDNA sequences to WA28KT except for two ambiguous nucleotides; we treat both as *P.* aff. *u. uniformis*. Partial sequences *P. u. hemiradia* (931 nt) and *P.* aff *uniformis* (WA32KAG) (1417 nt) differ by one nucleotide substitution and an ambiguity possibly sequencing errors.

***Paraphysomonas uniformis hemiradia*** subsp. n. (AU30KV) **Comment:** *P. u. hemiradia* fed upon a very small chrysophyte (*Oikomonas*-like) contaminant. We are unsure whether the radial spoke effect of *P. u. hemiradia* is partially a creasing artefact during TEM preparation or a permanent structure, but in either case its reproducibility must reflect a basic difference from *P*. *u. uniformis*. The scales may cope with the large cell being very plastic by the large base plate really being concave or convex over the cell as it changes shape; the cell makes pyriform as well as elongate shapes. The silica could be semi-plastic too, the structure accounting for the radical cell movement.

**Clade B**

The next two species (plus morphologically similar or uncharacterised lineages) form the long-branch clade B that is exclusively freshwater (Figure 1).

***Paraphysomonas cambrispina*** sp. n. (WI34KN) **Comment:** none

***Paraphysomonas vulgaris vulgaris*** sp. n. (PML2B) **Comment:** The culture contained an obvious non-*Paraphysomonas* contaminant; non-photosynthetic colony-forming flagellated cone-shaped chrysophyte, on which it is thought to have fed, as well as on bacteria and other smaller *Paraphysomonas*. The transverse crease is visible on the base-plate when the spine has fallen to one side during specimen preparation; as this crease is typically absent when the spine has broken off it is probably an artefactual, albeit reproducible, distortion of a basically symmetric baseplate. Two other freshwater strains, **SW02**, (Zurich, CH. River Stahl near meeting with River Limnat, Switzerland. JMS), and **PML2Af2** (non-flowing stream, Oxford, UK. JMS) have just two nt differences in 18S rDNA from *P. v. vulgaris* and identical sequences to each other and may be another closely related species, but we designate them both simply as *P.* aff*. v. vulgaris*; as our data are incomplete for each we cannot be sure that they are morphologically identical. SW02 was dead before cell measurements could be taken, but TEM (Figure 7A-B,) shows very similar scales: spine 3.3 µm (1.2-5.4) gently tapering from commonly wide slightly bulbous base to oblique dull pointed tip; spine often crooked or slightly bent. Base plate 1.8 µm (1.3-2.3), round to oval with thickened inflexed rim with transverse crease across centre. S/P ratio 1.8 (0.9-2.7). Strain PML2Af2: CL 10.6 µm (7.7-18.2), conspicuous scale base layer on a plastic cell, has no TEM data. All these strains exhibit an obvious scale base layer. An isolate not from our study, ‘Monas’ sp TON-O (AB168053), is sister to SW02 in this clade.

***Paraphysomonas*** aff. ***vulgaris vulgaris*** (strain W03) Figure 8E-F. M**easurements:** CL 11.3 µm (8.2-12.7: N= 17); LC x 2 CL, SC x 0.5 CL. Large plastic oval to round cell often bright. Smooth meandering swimmers, oval to pyriform. Scale base layer visible in LM. Trailing stalk and detritus, common. Scale morphology unknown although expected to be similar to other members of Clade B. Strain **W03**. (Stream up from beach, Nolton Haven, Wales, UK. JMS). Freshwater. 18S rDNA sequence **GenBank** JQ967316. **Comment:** Plasticity of this cell can be impressive, reaching very large sizes; one can often see other smaller *Paraphysomonas* cells inside a larger cell. W03 branches near the base of Clade B (Figure 1) that has the most isolates from this study (nine other isolates had identical SSU to others in the clade), from which this species has up to a dozen nt differences.

***Paraphysomonas vulgaris brevispina*** subsp. n. (PML4B) **Comment:** Eight other strains have identical 18S rDNA: **PML8** (Port Meadow, Oxford, UK. JMS) **SW03** (Lake Konstanz, Switzerland. JMS), **Ind7** (Stream, tropical India. JMS), **CCL3A** (Stream, Oxford, UK. JMS), **PML5B** (River, Oxford, UK. JMS), **GMDL3-g4** (Seasonal pond, Shropshire, UK. JMS) **W04** (Weir, Pembrokeshire, UK. JMS), **W07** (Stream leading to sea, Pembrokeshire, UK. JMS), all now identified as *P. vulgaris* but most not assignable to subspecies without scale pictures. **Ind7** has TEM data matching more closely subspecies *P. v*. *vulgaris*, spine 2.7 µm (2-4.1), base plate 1.8 µm (1.3-2.1) S/P ratio 1.5 (1.1-2.2), suggesting that this subspecies is found in both temperate and tropical habitats; the other isolates with the same SSU will probably have scales more or less like the species or subspecies. Two sequences, one from our study BZ5b (JQ967312) with just two nucleotide differences from *P. v. vulgaris*, and a freshwater lake isolate ‘Monas’ TON-0 (AB168053), published only in GenBank, differ by just one nucleotide substitution and deletion, so we deem both *P.* aff. *v*. *vulgaris*.

**Clade C**

The species and less well-characterised lineages form the second major long-branch freshwater clade, Clade C (Fig 1):

***Paraphysomonas variosa*** sp. n. (IND5) **Comment:** The species name refers to the variable spine lengths and breadths, not the very uniform width of the base plate. The extremely varied spine length and its basal width give scales a very non-uniform appearance and highly variable S/P ratio. Our strain **EP4** (Freshwater, Somerset, UK; GenBank JQ967297; no microscope data) had one nucleotide difference from *P. variosa*: we call it *P.* aff. *variosa*.

***Paraphysomonas caroni*** sp. n. (CH2) **Comment:** This was a mixed culture: *P. caroni* and notably smaller chrysophyte flagellate on which it fed, shown in Figure 10K. Three additional strains are regarded as *P.* aff. *caroni*; **PV10** (AF109325 - second isolation from CCAP 935/14 ‘*P. vestita*’. Freshwater); and strain **TGS6** (AB616676 - Miyagi, Sendai-shi, Japan Hirose River. Freshwater) have identical 18S rDNA and differ in sequence from CH2 by just two nts; strain **JBAS37** (AY651094 Freshwater, Kathmandu, Nepal) has three different nts.

***Paraphysomonas mantoni*** sp. n. (BZ5a) **Comment.** The culture had unusual diversity of cell sizes. TEM was done twice, over a year apart, and yielded the same variation of spine scales, so the two spine tip morphologies seems to be stable. We are unsure whether the two forms of spine tip are different views of the same thing, true intrastrain variation, or a sign that the culture is mixed. We did not have time to re-clone the cells and repeat the electron microscopy a third time or re-sequence fresh single-cell isolates, which could, in principle, be done to test the latter possibility. The fact that the next species, which we isolated twice and which is genetically so close to *P. mantoni* that they cannot be distinguished on the tree, but whose 18S rDNA sequence differs in 11 respects, is also more variable in the light microscope than most strains (but less so than BZ5a) makes it possible that both species are intrinsically unusually variable and that BZ5a is not mixed.

***Paraphysomonas*** aff. *mantoni*. (Bassen) Figure 10O**. Measurements**: CL 10.1 µm (8.2 - 13.6: N = 22); LC 1.5-2.0 x CL; SC 0.5-0.75 x CL. Large plastic oval to round cell, sometimes bright, sometimes becoming pyriform or very thin and elongate when swimming. Scale base layer visible. No TEM data. Strain **Bassen**. (Freshwater, Bassenthwaite, Cumbria, UK. JS). 18S rDNA sequence, GenBank JQ967294. **Comment:** This culture shows a range of size and shapes of cells. Its sequence places in a large freshwater clade next to *P. mantoni*. The tree shows slight genetic distance between *P. mantoni* and this strain; however there are eight indels between them (which would not affect trees), and 3 distinct nt differences, so they are probably separate species, even so, we a conservative in our decision to make it ‘aff’. Strain **SW04** (Lake of the four cantons (Vierwald Stattersee), Luzern, Switzerland. JMS) CL 10.6 µm (8.6 - 14.6; N=23), here called *P.* aff. *mantoni*, has just one indel and nt difference from ‘Bassen’ strain.

***Paraphysomonas petronia*** sp. n. (J1) **Comment.** none

**Clade D**

The next set of species form a long-branch subclade with a species from soil, freshwater, and marine habitats (Fig. 1) with relatively long spines in Clade D:

***Paraphysomonas solis solis*** sp. n. (GMCCL6) **Comment:** Culture difficult to interpret because of ‘granule’-like bodies ~1 µm in culture; the TEM showed small very round scaleless chrysophytes up to 5 µm in diameter: these smaller cells were not included in cell length measurements, even though the smaller cells, the granules might possibly be a life stage of this *Paraphysomonas.* However, we think *P. solis* ate them.

***Paraphysomonas solis crocotilla*** subsp. n. (UPL1B) **Comment:** Stomatocysts observed in live culture, but culture mixed with another non-*Paraphysomonas* swimming flagellate. *P. crocotilla* is most similar to *P. dimorpha* (in the same clade) because of the likely occasional presence of a spineless scale in addition to spine scales. *P. crocotilla* is on average larger than *P. dimorpha*, though. However, their cell length ranges partially overlap. They also share a similar S/P ratio (~3.0) and scale bases have similar sizes, but *P. crocotilla* averages a longer spine by 0.5 µm. The spineless scale measured for *P. crocotilla* is larger than the average size of its spine-scale bases, as noted below for *P. dimorpha*. As the spine of *Paraphysomonas* scales probably grows by evaginating from a primary disc (Hibberd 1979; Lee 1978), developmental failure of such evagination could produce a larger disc given the same amount of starting material. *P. spiculosa* has similar spine scales to *P. crocotilla*. However, spines are shorter and tips have a smaller point than *P. spiculosa* with a subtly rounded tip; with the same tapering gradient *P. spiculosa* with a longer spine would appear thinner. Morphological differences between these soil species are relatively slight compared with their very large 18S rDNA divergence. *P. crocotilla* ate very small chrysophyte (*Oikomonas*-like) contaminants.

***Paraphysomonas dimorpha*** sp. n. (CA01) **Comment:** A smaller cell body, thought to be a contaminant was also present in the culture (Figure 11H), but perhaps dormant cells preparing to encyst although cysts not readily observed. Spine length varies greatly but base plate is consistently ~1.1 µm. Spineless scales are more varied in size and can be more oval as well as exhibiting an obvious short line in the centre (not from a broken off spine, because that would leave a hole whence the wide base of the spine had protruded). The regularity of the spine scale base plate measurements, plus larger size of spineless scales, also make confusion with base plates of broken spine scales unlikely. For fuller comparison with similar species, see *P. crocotilla* above. Strain **Mike** (lawn mud/grass, Staffordshire, UK; very mixed, so not processed for TEM or DIC measurements. JMS) had the same 18S rDNA sequence showing it to be a geographically widespread soil species.

***Paraphysomonas stylata*** ***stylata*** sp. n. (W02) **Comment:** none

***Paraphysomonas stylata limnetica*** subsp. n. (PML5D) **Comment:** Base plate sizes vary a lot as does spine length (giving large S/P ratio range), as in *P. longispina* sp. n. (below). However, its 18S rDNA is much closer to *P. s. stylata*; only 4 nts differ. These two closely related species have 4nt differences, two significant, when compared with other species variation across the whole alignment. Despite genetic closeness, subtle differences in morphology exist. It is difficult to interpret the *P. s. limnetica* tip because TEMs are not clear enough.

***Paraphysomonas longispina*** sp. n. (MEX3) **Comment:** The base of spines is sometimes inflated or cone-shaped and compressed in TEM preparation, we often saw base plates with detached spines showing ‘pinched’ remnant of where the spine started. Aberrant scale forms had deformed or incomplete spine. Three other of our strains have identical 18S rDNA, the **only** example of 18S rRNA identity from both marine and freshwater strains: **CA03**, from a fresh water outlet to sea (stream, not observed by TEM), **MEX1** (marine, Coatzacoalcos, Veracruz, Gulf of Mexico. JMS), and **VA02** (brackish water, treated as marine, Lee Mont, Virginia, Chesapeake Bay, USA; not observed by TEM. JMS). These four samples being marine and ‘fresh’ water close to the sea (all are from coastal America) may indicate tolerance of salinity changes or brackish water specialists; but as we did not study ITS rDNA sequences we cannot rule out the possibility that those from marine or freshwater are separate cryptic species with identical 18S rDNA. The only TEM picture of a MEX1 scale resembles *P. longispina* (type strain: MEX3), but had a shorter spine; the cell is also slightly smaller; CL, 7.7 µm (6.4-8.6: N=29), but for now we treat MEX1, CA03, and VA02 all as *P. longispina*. The *P. longispina* rDNA has 1 nt different from marine strain ‘*vestita*’ SOTON1 (Z28335) from Southampton Water, UK, for which a scale had dimensions within the *P. longispina* range, spine ~ 6.2 µm, base plate ~1.2 µm, and closely similar morphology, but without an inflated spine base (Rice et al. 1997 fig. 1A). We regard it as *P.* aff. *longispina*, misidentified as freshwater *P. vestita,* with longer and thicker spines easily seen by LM.

**Clade E**

The next set of species form a robust clade (Fig. 1), Clade E, which is exclusively from soil and has a tendency to form shrunken smaller cells in culture. The last three readily encyst as stomatocysts of distinctive morphologies:

***Paraphysomonas sinensis*** sp. n. (CH9) **Comment:** Difficult to maintain the culture; strange smaller (~5 µm) dormant-looking cells observed, perhaps a life stage. Scales unusually non-uniform; the larger spineless plate scales could be malformed spine scales, but unlike in *P. crocotilla* (above) has intermediates with shorter, stubbier spines than typical spine-scales.

***Paraphysomonas* sp.** (BZ1) Figure 14F. **Measurements:** CL 7.6 µm (6.8-8.2 N=19); LC 2.5 x CL; SC 0.75 x CL. Oval to round cells with scale base layer visible in LM. Swimming cell oval to elongate. Cells >2 µm smaller than *P. sinensis*, with slightly longer LC. 18S rDNA of *P. braziliensis* and *P. sinensis* (sisters on Fig. 1) differ by >12 nts. No TEM or stomatocyst data. strain: **BZ1**. (Soil, Brazil. JMS). Freshwater. 18S rDNA sequence GenBank JQ967304.

***Paraphysomonas spiculosa spiculosa*** sp. n. (BZ8) **Comment:** none

***Paraphysomonas spiculosa terricola*** subsp. n. (GMBGL1) **Comment:** Strain **GMSTL1,** from soil in Sandon Staffordshire, UK had identical 18S rDNA (in parenthesis Fig. 1). The golf course soil **GMAL1** (Audlem, Cheshire, UK; GenBank JQ967300) 18S rDNA differs by one nucleotide (and one undetermined nt) from *P. terricola*; both exhibit very distinct insertions, more than 6, all along, so we call GMAL1 *P.* aff*. terricola*; plugged stomatocysts with a long collar and pyriform and oval swimming cells noted in culture but not measured.

***Paraphysomonas spiculosa edaphica*** subsp. n. (CH6) **Comment:** Difficult to interpret culture because of ‘granule’-like bodies, possibly dormant or starved cells, as in *P. solis solis*, another soil strain in a fairly distant sister clade. Only a few cells observed and measured in LM, and no TEM scale data; 18S rDNA is distinct in three areas, from its closest relatives, with two sets of two deletions (between positions 119-147) and two other nt differences at positions 59 and 1361.

***Paraphysomonas* sp.** (IND4) Distinguished from all other species by 18S rDNA sequence with at least 5 nts different from closest relatives *P. spiculosa and P. edaphica*. Sequence GenBank JQ967299. Large cells; strain **IND4**. (Soil, India. JMS). **Comment:** No data for cell dimensions or scales as the strain died before measurements or TEM: the culture was difficult to maintain, like many soil strains, and became overgrown by another smaller ~5.5 µm chrysomonad, possibly a *Paraphysomonas*. *P. edaphica* 18S rDNA differs by 5 nucleotides in highly diverse regions not included in the mask for trees. *Paraphysomonas s*train IND4 and *P. spiculosa* are arbitrarily together on the tree because they share two similarities that are included in the mask.

***Clathromonas***

***Clathromonas butcheri*** (Strain MD03, CCAP 936/1) **Comment:** Images from the original description (Pennick and Clarke 1972) strongly show LC hairs because of shadowing with gold-palladium whereas our sample was not metal-shadowed so the hairs were less conspicuous. Otherwise our new strain of *Clathromonas* *butcheri* matches nearly exactly the original description down to the brackish habitat. However, the original *C. butcheri* were noted as having smaller cells with proportionally longer cilia (via multiples of cell diameter) than our strain. That apparent difference is probably an artefact because Pennick and Clarke measured dead, fixed cells and there must have been shrinkage in their preparation compared with living cells, as there was in ours which showed the exact dimensions as Pennick and Clarke’s when fixed. The description of scales and number of holes in the outer ring of the plate scales are the same in both strains; a wider range of number of holes in the inner ring (9-13, compared to 10-12), a negligible difference probably arising from a difference in the number of scales measured. The basket scales look are extremely similar, though the strut dimensions are hard to compare as we not know at what points their measurements started and ended, but the range of sizes of our struts are happily close to theirs (0.24-0.31 µm) compared to their one measurement (0.24 µm).

***Clathromonas tongi*** sp. n. (SOTON-A) **Comment:** Even though the sequence is partial, just 328 nucleotides, this region unambiguously places it within the ‘*butcheri*’ clade (along with the TEM data) and distinguishes it from close relatives.

***Clathromonas caroni*** sp. n. (DB4) **Comment:** none

***___________________________________________________________________________________________________________________***

**PART TWO. Discussion: Major environmental chrysophyte clades**

Previously del Campo and Massana (2011) found three major environmental DNA clades of putative chrysophytes of unknown phenotype: clades G (corresponding to our EC1) H (corresponding to our EC2H) and I (corresponding to our EC2I). Their RAxML analysis grouped clades G and H as sisters and clade G/H as sister to *Paraphysomonas* sensu stricto (their clade F1), with *Clathromonas* (their clade F2) sister to that joint clade, all without BS support. Our analyses of both datasets found the same three well-supported environmental clades, but neither grouped EC2H with EC1 and they were differently grouped in each, and both differed from the topology of del Campo and Massana (2011), whose tree (their fig. 3) included some chrysophyte sequences not in ours (but also excluded many more on our tree) but was rooted on only a single outgroup species (the eustigmatophyte *Nannochloropsis*), making its rooting even more problematic and leaving the chrysophyte nature of clades G-I open to some doubt. Our extensive outgroup analysis unambiguously confirms that all are chrysophytes, though if EC2 is the deepest branch, we cannot be sure whether stomatocysts evolved before or after its divergence (Supplementary Fig. S1). As noted above, the position of subclade EC2H is uncertain and sensitive to taxon composition of outgroups – it may be sister to EC2I (Supplementary Fig. S1 with well sampled outgroups) or to EC1 (Fig. 2 with only sparsely sampled but more closely related outgroups) or to Hydrurales (clade D in del Campo and Massana 2011). Both EC1 and EC2H include freshwater and marine clones (typically clustering separately) and some longer branching divergent sequences, which could account for the unstable position and splitting of EC2. Interestingly marine subclades have substantially longer branches and thus faster rDNA evolution than freshwater ones; clade EC2I is entirely marine and thus includes no short-branch representatives, which may help explain why its position is unstable – grouping with EC2H on Supplementary Fig. S1 and with EC1 in del Campo and Massana (2011).

Charvet et al. (2011) found two clusters of chrysophyte environmental DNA sequences from High Arctic lakes that they could not assign to an existing order, even suggesting that cluster I might be outside and sister to Chrysophyceae. Our more comprehensive sampling (Supplementary Fig. S2) shows that this is incorrect and that both clusters branch reliably within specific chrysophyte orders. Cluster I is a deep branch within Chromulinales and II belongs in the clade here designated Apoikiida (as it includes *Apoikia*). Their misinterpretation arose from the tree being rooted on a too distant single (dinoflagellate) outgroup and not having any non-chrysophyte ochrophyte sequences. Their ‘novel clade’ Environ1 is not sister to *Paraphysomonas* as on their sparsely sampled tree but part of EC2H (Supplementary Fig. S3). Their clade Environ2 is part of one of the Chromulinales novel clades. Their clade Environ3 is part of EC1Ga and Environ4 part of EC1. We therefore conclude that only the three major environmental clades of del Campo and Massana (2011) cannot confidently be assigned to a specific order. If the topology of either our Bayesian tree in Supplementary Fig. S1 or of del Campo and Massana (2011) figure 3 were correct, then there would really only be two major phenotypically undetermined chrysophyte clades.

We hypothesise that Supplementary Fig. S1 with diverse balanced outgroups is less likely to be subject to idiosyncratic misrooting than Supplementary Fig. S3, and that EC1 may therefore be relatively closely related to Paraphysomonadidae and Clathromonadidae (also found by del Campo and Massana 2011), and so may also consist of scaly heterotrophic biflagellate phagotrophs. As Supplementary Fig. S3 indicates, there are three very distinct multispecies environmental subclades within Chromulinales – one sister to *Chrysophaerella* so quite likely to have scales also. The deep-branching position and instability of clade EC2 makes it harder to predict its phenotype; though we cannot exclude the possibility that it includes species with scales, the most parsimonious interpretation assuming the Bayesian tree of Supplementary Fig. S1 to be correct is that scales evolved after EC2 and Hydrurales diverged, so a reasonable guess is that EC2 cells are naked phagotrophs. Our trees agree with earlier ones (Andersen 2007; Cavalier-Smith and Chao 2006) in three respects: (1) resolution at the base of Chrysophyceae is very low; (2) *Paraphysomonas* is a very distinct clade from both the scaly Synurales and non-scaly Hibberdiales; and (3) Ochromonadales as previously constituted consists of two major clades, one including all *Spumella* and most *Ochromonas* (Ochromonadales on Fig. 2), and one that includes *Lagynion*, *Chrysosphaera* and *Chrysosaccus*. We have now shown that the latter (Apoikiida on Fig. 2) also includes the purely heterotrophic phagotroph *Apoikia* (Kim et al. 2010). Only for Paraphysomonadida is there some doubt about its monophyly; if EC1 is phagotrophic and has scales, and thus belongs to Paraphysomonadida, it may be holophyletic. Paraphysomonadida including EC1 is strongly holophyletic on trees with an extensive balanced set of outgroups (Supplementary Fig S1), but appears not to be on trees rooted just on Picophagea (Fig. 2), which we argued above may be a rooting artifact caused by a restricted unrepresentative outgroup combined with some long-branch taxa being attracted towards it. Carefully rooted multigene trees are needed to test its monophyly further. Thus seven or eight phylogenetically distinct clades on the chrysophyte sequence tree have phenotypically identified representatives.

Our trees also show fewer radically distinct clades comprising only environmental sequences than previously thought (del Campo and Massana 2011; Charvet et al. 2011). On the MrBayes tree of Supplementary Fig. S1 there are two such clades; environmental clade 1 and 2 (EC1, EC2), but on the ML tree of Supplementary Fig. S1 and by both methods on Figure 2 EC2 is split, one half grouping with EC1 in Figure 2. It is not known whether EC1 and 2 are photosynthetic or not, but as they are so speciose establishing their phenotype is very important for understanding deep phylogeny of Chrysophyceae. In the absence of such knowledge, and if Paraphysomonadida is holophyletic, it is the only purely heterotrophic and phagotrophic order within Chrysophyceae and photosynthesis was lost once in the last common ancestor of Paraphysomonadidae and Clathromonadidae. Hydrurales, Synurales, and Hibberdiales on present knowledge are purely photosynthetic, non-phagotrophic algae, whereas Chromulinales, Apoikiida, and Ochromonadales all have a mixture of often phagotrophic algae and of purely phagotrophic non-algal protists (*Oikomonas*, *Apoikia* and *Spumella* respectively). As there are four distinct *Spumella* clades Ochromonadales lost photosynthesis at least four times. Thus, there were at least seven distinct losses of photosynthesis in Chrysophyceae and the class should not be thought of as typically algal. Clearly, it was ancestrally phagotrophic and phagotrophy and photosynthesis were each lost more than once. The absence of heterotrophy in the probably non-phagotrophic orders suggests that retention of phagotrophy facilitated the multiple independent losses of photosynthesis and the reversion to a more protozoan way of feeding. It is also evident from our trees that there were several independent losses of the posterior cilium and at least two independent losses of both cilia in chrysophytes.

**REFERENCES**

Andersen, R.A., 2007. Molecular systematics of Chrysophyceae and Synurophyceae. In: Brodie J, Lewis J (eds) Unravelling the algae: the past, present and future of algal systematics. Boca Raton, CRC - Taylor and Francis, pp 285-353.

Atkins, M.S., Teske, A.P., Anderson, O.R., 2000. A survey of flagellate diversity at four deep-sea hydrothermal vents in the Eastern Pacific ocean using structural and molecular approaches. J. Euk Microbiol 47, 400-411.

Boenigk, J., Pfandl, K., Stadler, P.F., Chatzinotas, A., 2005. High diversity of the '*Spumella*-like' flagellates: an investigation based on the SSU rRNA gene sequences of isolates from habitats located in six different geographic regions. Env Microbiol 7, 685-697.

Caron, D.A., Lim, E.L., Dennett, M.R., Gast, R.J., Kosman, C., DeLong, E.F., 1999. Molecular phylogenetic analysis of the heterotrophic chrysophyte genus *Paraphysomonas* (Chrysophyceae), and the design of rRNA-targeted oligonucleotide probes for two species. J Phycol 35, 824-837.

Cavalier-Smith, T., Chao, E.E., 2006. Phylogeny and megasystematics of phagotrophic heterokonts (kingdom Chromista). J. Mol. Evol. 62, 388-420.

Charvet, S., Vincent, W., Lovejoy, C., 2011. Chrysophytes and other protists in High Arctic lakes: molecular gene surveys, pigment signatures and microscopy. Polar Biol. 35, 1-16.

del Campo, J., Massana, R., 2011. Emerging diversity within chrysophytes, choanoflagellates and bicosoecids based on molecular surveys. Protist 162, 435-448.

Hibberd, D.J., 1979. Notes on the ultrastructure of the genus *Paraphysomonas* (Chrysophyceae) with special reference to *P. bandaiensis* Takahashi. Arch. für Protistenk. 12, 146-154.

Kim, E., Yubuki, N., Leander, B.S., Graham, L.E., 2010. Ultrastructure and 18S rDNA phylogeny of *Apoikia lindahlii* comb. nov. (Chrysophyceae) and its epibiontic protists, *Filos agilis* gen. et sp. nov. (Bicosoecida) and *Nanos amicus* gen. et sp. nov. (Bicosoecida). Protist 16, 177-196.

Lee, K.S., Takahashi, E., 1993. Studies on Paraphysomonadaceae (Chrysophyceae) from Andong Lake in Korea. Nord. J. Bot. 13, 211-220.

Lefranc, M., Thénot, A., Lepère, Debroas, D., 2005. Genetic diversity of small eukaryotes in lakes differing by their trophic status. Ap Env Mircobiol 71, 5935-5942.

Lucas, I.A.N., 1967. Two new marine species of *Paraphysomonas.* J Mar Biol Ass UK 47, 329-334.

Preisig, H.R., Hibberd, D.J., 1982. Ultrastructure and taxonomy of *Paraphysomonas* (Chrysophyceae) and related genera 1. Nord J Bot 2, 397-420.

Rice, J., O'Connor, C.D., Sleigh, M.A., Burkill, P.H., Giles, I.G., Zubkov, M.V., 1997. Fluorescent oligonucleotide rDNA probes that specifically bind to a common nanoflagellate, *Paraphysomonas vestita*. Microbiol **143**, 1717-1727.

Takahashi, E., 1976. Studies on genera *Mallomonas* and *Synura*, and other plankton in freshwater with the electron microscope X. The genus *Paraphysomonas* (Chrysophyceae) in Japan. Br Phycol J 11, 39-48.
