# Supplementary material for: Scale evolution in Paraphysomonadida (Chrysophyceae): Sequence phylogeny and revised taxonomy of Paraphysomonas, new genus Clathromonas, and 25 new species
Source: Eur J Protistol. 2014 Oct;50(5):551–92. doi: 10.1016/j.ejop.2014.08.001 (PMC4238902; doi:10.1016/j.ejop.2014.08.001)
Supplement: Supplementary file 2 [file mmc6.pdf]

Supplementary Fig. S1 Bayesian tree for 329 ochrophyte 18S rDNA sequences (1672 nucleotide positions). To emphasize the tree's main features and fit it onto one page internal branches of all major clades are collapsed, with the number of species included in each shown on the right (their internal topology is shown in other figures). Support values are MrBayes posterior probabilities (left) and RAxML bootstrap percentages for 1000 pseudoreplicates to the right. Black dots mean maximal support for both, i.e. 1/100. Arrows suggest possible timings of the origins of scales and stomatocysts within the Chrysophyceae.

Supplementary Fig. S2 Bayesian tree for 329 ochrophyte 18S rDNA sequences (1672 nucleotide positions) showing the branching order of Chrysophyceae other than *Paraphysomonas* sensu stricto and the immediate outgroup Picophagea only in detail. Support values are MrBayes posterior probabilities (left) and RAxML bootstrap percentages for 1000 pseudoreplicates to the right. Black dots mean maximal support for both, i.e. 1/100. The only new sequence (for *Clathromonas butcheri*) is in bold type.

Supplementary Fig. S3 Bayesian tree for 329 ochrophyte 18S rDNA sequences (1672 nucleotide positions) showing uncollapsed nodes of the rest of the Chrysophyceae: clades of named Chrysophyeae and Environmental Clades, 1 and 2. MrBayes and RaXML values shown, respectively, at each node.

Supplementary Fig. S4 Comparison of the cells and scales new species of *Paraphysomonas*. For each there are DIC light micrographs of live cells and an example of a spine scale all at the same relative magnifications. The diversity of size and proportions of the scales is just as notable as the size of the cells. The close-ups of spine tips are not to the same scale, each being magnified sufficiently to show its characteristic shape; the differences in tips of the spines are quite subtle but stable within a strain and rather diverse; closely related species generally have very similar spine tips. Scale bar: scales 1 μm. DIC live cells, 5 μm.
